# Bayesian Methods for Meta-Analyses of Binary Outcomes: Implementations, Examples, and Impact of Priors

**DOI:** 10.3390/ijerph18073492

**Published:** 2021-03-27

**Authors:** Fahad M. Al Amer, Christopher G. Thompson, Lifeng Lin

**Affiliations:** 1 Department of Mathematics, College of Science and Arts, Najran University, Najran 55461, Saudi Arabia; fma16@my.fsu.edu; 2 Department of Statistics, Florida State University, Tallahassee, FL 32306, USA; 3 Department of Educational Psychology, Texas A&M University, College Station, TX 77843, USA; cgthompson@tamu.edu

**Keywords:** Bayesian analysis, Markov chain Monte Carlo, meta-analysis, odds ratio, prior distribution

## Abstract

Bayesian methods are an important set of tools for performing meta-analyses. They avoid some potentially unrealistic assumptions that are required by conventional frequentist methods. More importantly, meta-analysts can incorporate prior information from many sources, including experts’ opinions and prior meta-analyses. Nevertheless, Bayesian methods are used less frequently than conventional frequentist methods, primarily because of the need for nontrivial statistical coding, while frequentist approaches can be implemented via many user-friendly software packages. This article aims at providing a practical review of implementations for Bayesian meta-analyses with various prior distributions. We present Bayesian methods for meta-analyses with the focus on odds ratio for binary outcomes. We summarize various commonly used prior distribution choices for the between-studies heterogeneity variance, a critical parameter in meta-analyses. They include the inverse-gamma, uniform, and half-normal distributions, as well as evidence-based informative log-normal priors. Five real-world examples are presented to illustrate their performance. We provide all of the statistical code for future use by practitioners. Under certain circumstances, Bayesian methods can produce markedly different results from those by frequentist methods, including a change in decision on statistical significance. When data information is limited, the choice of priors may have a large impact on meta-analytic results, in which case sensitivity analyses are recommended. Moreover, the algorithm for implementing Bayesian analyses may not converge for extremely sparse data; caution is needed in interpreting respective results. As such, convergence should be routinely examined. When select statistical assumptions that are made by conventional frequentist methods are violated, Bayesian methods provide a reliable alternative to perform a meta-analysis.

## 1. Introduction

Systematic reviews and meta-analyses have been a popular tool for synthesizing evidence in many fields, including evidence-based medicine, public health, and environmental research [1,2,3]. Through systematic reviews and meta-analyses, researchers can combine findings from independent studies on common research topics, with one goal being to produce overall results that may be more precise than individual studies.

Coming along with the mass production of meta-analyses in recent years, the quality of many meta-analyses may be low, being partly related to certain methodological limitations [4]. For example, the method that was proposed by DerSimonian and Laird [5] is the most widely-used meta-analysis approach, with over 30,000 Google Scholar citations as of March 21, 2021. However, this approach has been shown to be outperformed by several alternative estimation methods, such as the restricted maximum-likelihood (REML) [6,7]. In addition, conventional meta-analysis methods often assume that within-study sample variances are known, fixed values. Thus, sampling errors from within-study variances are ignored, which may be unrealistic and they can lead to substantial biases in some situations (e.g., small sample sizes) [8,9]. For example, the sample variance of the log odds ratio (OR) is typically calculated as 1/a+1/b+1/c+1/d, where a, b, c, and d represent the four data cells in a 2 × 2 table for a binary outcome. This sample variance is conventionally treated as a fixed variable in a meta-analysis, although the four data cells are actually random variables. More advanced frequentist approaches, such as generalized mixed-effects models, can avoid this unrealistic assumption, particularly for meta-analyses of binary outcomes [10,11,12,13]. Many simulation studies have compared various frequentist methods for meta-analyzing binary outcomes and they have offered useful recommendations under different scenarios [14,15,16,17]. However, these approaches may require maximizing certain likelihood functions that involve complicated integrals and are generally less familiar among meta-analysis practitioners.

When comparing frequentist approaches to meta-analysis, Bayesian methods are widely used in medical research among other fields, and they provide a flexible way for implementing complicated models [18,19,20,21,22,23,24]. Another major benefit of Bayesian methods is that prior information can be explicitly incorporated in meta-analytic models and, thus, have an impact on the results [25]. In the literature of multivariate meta-analysis of multiple outcomes and/or multiple treatments, Bayesian methods are a standard approach for effectively modeling complicated variance-covariance structures [26,27]. However, frequentist methods still dominate conventional univariate meta-analyses that compare each pair of treatments for each outcome separately [28]. The widespread use of frequentist methods in univariate meta-analyses is partly due to the relatively easy implementation in several popular software packages [29]. Bayesian methods, on the other hand, typically require nontrivial statistical coding, which may limit their promotion in applications. Fortunately, with the advance of meta-analysis methodology, more software programs are being developed to provide user-friendly functions for implementing Bayesian meta-analyses. For example, the R package “bayesmeta” contains a collection of functions for deriving and evaluating posterior distributions of parameters in a random-effects meta-analysis [30]. Another R package “brms” fits Bayesian multilevel models and supports a wide range of distributions and link functions [31]. The R packages “gemtc” and “pcnetmeta” can be used to implement Bayesian network meta-analysis of multiple treatments [32,33]. The relatively new bayesmh command in Stata and BGLIMM procedure in SAS can be also used to fit Bayesian meta-analysis models.

The objectives of this article are three-fold, with a focus on univariate meta-analyses of ORs for binary outcomes. First, we review Bayesian methods, as well as frequentist methods, for conducting meta-analyses. As the heterogeneity of effect sizes plays a critical role when combining primary study results, we introduce three commonly used estimators of the between-studies variance under the frequentist framework. Under a Bayesian framework, we present four prior distributions with different sets of hyper-parameters. It is of note that this manuscript does not aim to provide recommendations for prior selection, because preferable priors may differ case-by-case, particularly when experts’ opinions are available to derive informative priors. Second, we present detailed steps to implement a Bayesian meta-analysis via the Markov chain Monte Carlo (MCMC), with an emphasis on the importance of validating the posterior samples (i.e., MCMC convergence). Third, we apply the various methods to data from five meta-analyses that were published in *The BMJ*. Based on these examples, we empirically examine the impact of different priors on the overall effect estimates, including point and interval estimates, and explore potential problems that are caused by sparse data.

## 2. Materials and Methods

### 2.1. Bayesian Meta-Analysis of Odds Ratios

The Supplementary Materials (Data A) review the frequentist meta-analysis methods. The frequentist framework assumes unknown parameters of interest to be fixed; by contrast, the Bayesian framework treats unknown parameters as random variables with assigned prior distributions. Bayesian methods have serval advantages over frequentist ones. For example, they can be easily applied to complicated models [34,35], improving convergence issues, and improving estimates using informative priors [36,37,38]. Nevertheless, the required computation is usually more extensive than frequentist methods [39]. Under the Bayesian framework, the estimation and inference of parameters are based on their respective posterior distributions. In some instances, such posterior distributions may not have explicit forms. To overcome this difficulty, MCMC algorithms are widely used to numerically draw samples from posterior distributions [40].

In order to implement Bayesian random-effects meta-analysis of ORs, we consider a meta-analysis containing k studies with binary outcomes; study i has $r_{Ti}$ and $r_{Ci}$ events in the treatment and control groups, respectively. The event counts are assumed to independently follow binomial distributions with sample sizes $n_{Ti}$ and $n_{Ci}$ within each study. The Bayesian hierarchical model is [41,42,43,44]:

$$r_{Ci}\;\sim Bin\left(n_{Ci},\pi_{Ci}\right),\;r_{Ti}\;\sim Bin\left(n_{Ti},\pi_{Ti}\right);\;\mathrm{logit}\left(\pi_{Ci}\right)=\mu_{i},\;\mathrm{logit}\left(\pi_{T_{i}}\right)=\mu_{i}+\delta_{i};\;\delta_{i}\;\sim N\left(\theta ,\tau^{2}\right);\;\mu_{i},\;\theta ,\;\mathrm{and}\;\tau \;\sim \;\mathrm{priors},$$

where $\pi_{Ti}$ and $\pi_{Ci}$ are the underlying true event rates in study i’s treatment and control groups. Additionally, $\mu_{i}$ denotes the baseline risk in each study. Because baseline characteristics may differ greatly across studies, researchers often treat it as a nuisance. Of note, one may also treat these baseline risks as random effects; we do not make this assumption in this article because there are ongoing debates about this assumption for randomized controlled trials [10,45,46,47].

Using the logit link function logit(t)=log[t/(1−t)] for the true event rates, $\delta_{i}$’s represent the underlying true log ORs within studies. To account for potential heterogeneity, they are assumed to be random effects, following a normal distribution with mean θ and variance $\tau^{2}$, where θ is interpreted as the overall log OR, and $\tau^{2}$ is the between-studies variance.

The vague normal prior $N\left(0,100^{2}\right)$ is frequently assigned for $\mu_{i}$ and θ; meta-analysis results are usually robust to the choice of priors for these parameters, as long as the priors are sufficiently vague. If available, more precise priors may be used for them based on experts’ opinions. Similar to the frequentist framework, where multiple estimators are available for $\tau^{2}$ [7], choosing the prior for the heterogeneity parameter is critical in Bayesian meta-analyses, especially when k is small or events are rare, as this prior may greatly impact the credible interval (CrI) length [48].

### 2.2. Priors for Heterogeneity

We consider four types of priors for the heterogeneity parameter: inverse-gamma, uniform, half-normal, and log-normal priors. Different hyper-parameters are used for each prior distribution. We choose these priors, because they have been used in many Bayesian meta-analyses. One may also tailor a specific prior using experts’ opinions on a case-by-case basis; such practice is out of the scope of this article.

The inverse-gamma prior, which is denoted by IG(α, β), is widely used for $\tau^{2}$. It is conjugate (producing a posterior within the same distribution family and, thus, yielding a closed-form expression) and facilitates computation [40]. Small parameter values are often assigned to the hyper-parameters α and β, which determine the distribution shape and scale, respectively. As both hyper-parameters approach zero, this prior corresponds to a flat prior for $\log\left(\tau^{2}\right)$. We consider setting both hyper-parameters as equivalent and to values 0.001, 0.01, or 0.1; these are common choices in practice [49]. The inverse-gamma prior may be advantageous for dealing with sparse data, as it may improve stability and convergence [50]. Nevertheless, because the inverse-gamma prior is not truly “non-informative”, the choice of hyper-parameters may have a substantial impact on meta-analytic results.

The uniform prior, denoted by U(0, c), is also widely used for the heterogeneity standard deviation τ [51], where c determines the prior’s upper bound. The lower bound is fixed to zero, as τ must be non-negative. We consider c = 2, 10, and 100 [52]. The justification for choosing an appropriate hyper-parameter c should be based on specific cases. For example, it might be reasonable to set c = 2 for log ORs, as they usually range between −2 and 2. However, when the effect measure is the mean difference for continuous outcomes, the choice of c should be based on the outcomes’ scales.

The half-normal prior, which is denoted by $HN\left(0,\sigma^{2}\right)$, is another candidate prior for τ [38,53]. This prior is a special case of the folded normal distribution, i.e., the absolute value of a random variable following $N\left(0,\sigma^{2}\right)$, where $\sigma^{2}$ controls the extent of heterogeneity. We consider $\sigma^{2\;}$= 0.1, 1, or 2 [38].

In addition, the evidence-based informative log-normal prior has been suggested for $\tau^{2}$ [54]. We denote this prior by $LN\left(\mu \;,\sigma^{2}\right)$; equivalently, $\log\left(\tau^{2}\right)$ has the normal prior $N\left(\mu ,\sigma^{2}\right)$. The hyper-parameters μ and $\sigma^{2}$ are derived from over 10,000 meta-analyses from the Cochrane Library, which may accurately predict the extent of heterogeneity of an external meta-analysis. As a result, the prior is viewed as “informative”, and it may be useful for meta-analyses with a few studies. The values of hyper-parameters depend on the type of treatment comparison and outcome in a meta-analysis, as detailed in Table 1. Treatment comparisons are classified as pharmacological treatment vs. placebo/control, pharmacological treatment vs. pharmacological treatment, or comparisons involved with non-pharmacological treatments (e.g., medical deviance, surgery), according to Turner et al. [54]. Outcomes are classified as all-cause mortality, semi-objective outcomes (e.g., cause-specific mortality), and subjective outcomes (e.g., mental health condition).

### 2.3. Implementation

**Figure 1:**
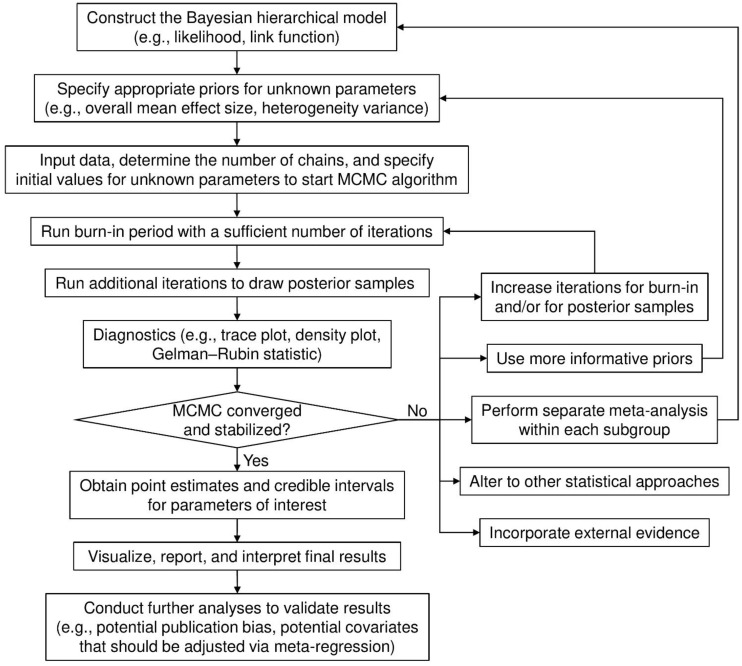
Flow chart for implementing a Bayesian meta-analysis.

Figure 1 shows the flowchart of a general process for implementing a Bayesian meta-analysis via MCMC algorithms. The burn-in period draws posterior samples early in the iteration process to achieve the convergence and stabilization of the Markov chains. Posterior draws during the burn-in period are discarded before making statistical inference. The diagnostic procedures are often overlooked in applications of Bayesian meta-analyses. If the Markov chains have not converged and stabilized, estimates that are based on the posterior samples may be invalid and/or misleading. Several factors may contribute to this problem. For example, if the number of iterations is insufficient, drawing more posterior samples may solve the problem. The convergence issue may also arise from a multi-modal posterior distribution, possibly because of subgroup effects. In this case, separate meta-analyses may be performed for each subgroup.

Various approaches, including trace and density plots, are available for diagnosing MCMC samples and their possible convergence. A trace plot presents the posterior samples after the burn-in period in each Markov chain iteration for each parameter. If it shows well-mixed samples, this provides evidence that the Markov chains have stabilized and converged. A density plot depicts the posterior sample density for each parameter. If the density shows multiple modes (e.g., “peaks”), then the Markov chains may not have converged. When Markov chains’ convergence and stabilization have been justified, point estimates (usually posterior medians), and their 95% CrIs, often formed by 2.5% and 97.5% posterior quantiles, can be computed for the Bayesian meta-analysis.

### 2.4. Application to Real Data

We used five real-world datasets with different sizes (number of studies and within-study sample sizes), event rates, and heterogeneity extents to illustrate the implementation of Bayesian meta-analyses, the impact of prior distributions on meta-analytic results, and the potential problems of MCMC convergence. All of the datasets had binary outcomes; regardless of original analyses, (log) ORs were used as effect measures in our re-analyses.

Example 1. The meta-analysis by Lamont et al. [55] investigated the risk of recurrent stillbirth from 13 cohort studies, all with large sample sizes. The comparison type was non-pharmacological (previous stillbirth vs. previous live birth), and the outcome (recurrent stillbirth) might be classified as semi-objective.

Example 2. The meta-analysis by Crocker et al. [56] combined eight studies to investigate the impact of patient and public involvement (PPI) on patient enrollment in clinical trials. The intervention PPI was non-pharmacological, and the outcome (enrollment in clinical trials) was likely semi-objective.

Example 3. The meta-analysis, as reported by Baxi et al. [57], included 13 studies to synthesize the association between anti-programmed cell death 1 (anti-PD-1) drugs and immune-related adverse events. This meta-analysis compared pharmacological treatments with control. In this example, the outcome was the adverse event colitis, which may be considered subjective according to Turner et al. [54] The event rate in each primary study was fairly low; many zero event counts existed.

Example 4. This meta-analysis is also from Baxi et al. [57]; it combined 15 studies with the adverse event hepatitis, which was also considered as a subjective outcome. The treatment comparison was also pharmacological treatments (anti-PD-1 drugs) vs. control. All of the studies had zero events in the control group.

Example 5. The fifth meta-analysis by Martineau et al. [58] collected 25 randomized controlled trials to assess the effect of vitamin D supplementation on the risk of acute respiratory tract infection (ARTI). The comparison was the pharmacological treatment (vitamin D supplementation) vs. control. The outcome was the experience with at least one ARTI, which might be considered to be subjective.

### 2.5. Statistical Analyses

For each dataset, we implemented the random-effects meta-analysis using the DerSimonian–Laird (DL) [5], maximum-likelihood (ML), and REML estimators [59] for $\tau^{2}$ under the frequentist framework and using the Bayesian models with 12 different priors for $\tau^{2}$ or τ. Specifically, we considered all four prior distributions that are reviewed in Section 2.2. For each of the inverse-gamma, uniform, and half-normal priors, we used three sets of hyper-parameters, as shown in Table 1. For the informative log-normal priors proposed by Turner et al. [54], we identified the treatment comparison type of each meta-analysis. Recall that the outcomes were classified as all-cause mortality, semi-objective outcomes, and subjective outcomes (Section 2.2). Such classifications might be ambiguous in some cases, particularly when distinguishing semi-objective and subjective outcomes. Therefore, according to the treatment comparison type of each meta-analysis, we used all three possible sets of hyper-parameters for three outcome types.

In the presence of zero event counts, when implementing the conventional frequentist methods, studies with zero counts in both the treatment and control groups had to be excluded (see Data A.3 in the Supplementary Materials for details). The continuity correction of 0.5 was applied to studies with zero counts in only a single treatment arm. Such ad hoc corrections were not needed in Bayesian analyses.

We used the R package “rjags” (version 4–7) to implement the Bayesian models via the MCMC algorithm. For each Bayesian model, we used three Markov chains, each having a burn-in period of 50,000 iterations, followed by 200,000 iterations for drawing posterior samples with thinning rate 2. Trace plots were used to assess the chains’ convergence. We obtained the posterior medians of the overall OR, the heterogeneity standard deviation $\hat{\tau }$, and 95% CrIs for both parameter estimates. The frequentist methods were implemented via the R package “metafor” [60]. We used the Q-profile method to obtain 95% confidence intervals (CIs) of $\hat{\tau }$ [61]. The Supplementary Materials (Data B) provide all of the statistical code.

This study did not require ethical approval and patient consent, because it focused on statistical methods for meta-analyses, and all the analyses were performed using publicly available data.

## 3. Results

**Figure 2:**
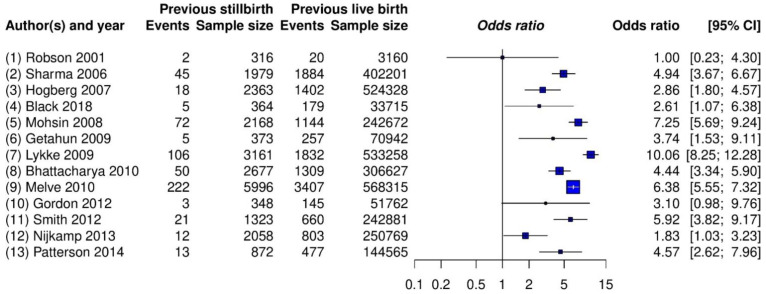
Forest plot of the meta-analysis on stillbirth (Example 1).

Table 2 provides the estimated ORs and heterogeneity standard deviations of the five real-world meta-analyses using all priors under the Bayesian framework, as well as three different estimators under the frequentist framework. All of the ORs were computed for comparing treatment against control. Figure 2 shows the forest plot for the meta-analysis on stillbirth; Figures S1–S4 in the Supplementary Materials (Data C) present those for the remaining four meta-analyses. In addition, Figures S5–S64 in the Supplementary Materials (Data D) provide trace plots related to MCMC processes, and Figures S65–S69 in the Supplementary Materials (Data E) present posterior density plots.

### 3.1. Example 1: Meta-Analysis on Stillbirth

The estimated overall ORs by all methods ranged from 4.30 to 4.59, and their CIs/CrIs were all above 1, which suggested a significantly increased risk of recurrent stillbirth among women with a previous stillbirth compared with those with a previous live birth. The results were relatively robust to the different meta-analytic methods, likely because this meta-analysis contained a moderate number of studies (k  = 13), with each study having many patients. These two combined qualities likely provided enough information for similar estimation results. The trace plots (Figures S5–S16) and posterior density plot (Figure S65) indicate that the Markov chains converged and stabilized well.

Nevertheless, different methods indicated some noticeable dissimilar point and interval estimates. For example, the frequentist DL, ML, and REML methods estimated the overall OR as 4.59, 4.52, and 4.47, respectively, and $\hat{\tau }$ was, accordingly, 0.38, 0.43, and 0.46. Among the Bayesian methods, the inverse-gamma and uniform priors produced fairly similar results. Those by the half-normal and log-normal priors led to some small differences. Specifically, as the hyper-parameter of the half-normal prior increased from 0.1 to 2, the estimated overall OR changed from 4.35 to 4.26, with $\hat{\tau }$ changing from 0.45 to 0.53 and yielding wider CrIs. The estimated overall ORs ranged from 4.31 to 4.39 for the log-normal priors.

In addition, the 95% CrIs that were produced by the Bayesian methods were generally wider than the 95% CIs produced by the frequentist methods. This was possibly because the frequentist methods failed to account for sampling errors within sample variances and the uncertainty in $\hat{\tau }$.

### 3.2. Example 2: Meta-Analysis on Patient Enrollment in Clinical Trials

All three frequentist methods produced $\hat{\tau }\;$ = 0, which then corresponded to the same estimated overall OR 1.16 with 95% CI [1.03, 1.30]. This suggested a statistically significant effect of PPI on patient enrollment in clinical trials. However, all of the Bayesian methods produced 95% CrIs containing 1, which indicated that the effect of PPI was not significant. The trace plots (Figures S17–S28) and the posterior density plot (Figure S66) indicate that the Markov chains converged and stabilized well.

The number of studies (k  = 8) was relatively small. The uniform and half-normal priors with different hyper-parameters led to fairly similar results. Nevertheless, the inverse-gamma prior had an influence on the results. As its hyper-parameters changed from 0.001 to 0.1, although the OR estimates remained nearly unchanged, the associated 95% CrI became much wider, changing from [0.99, 1.39] to [0.87, 1.57], with $\hat{\tau }$ changing from 0.09 to 0.29. The informative log-normal prior also had a noticeable impact on the 95% CrIs, and $\hat{\tau }$ was 0.10, 0.12, and 0.16 while using different hyper-parameters.

### 3.3. Example 3: Meta-Analysis on Colitis

This meta-analysis contained k = 13 studies; four studies had zero event counts in both arms, thus their ORs were not estimable, and they were subsequently removed when using the frequentist methods. All three frequentist estimators gave $\hat{\tau }\;$ = 0 and estimated the overall OR as 3.39 with 95% CI [1.45, 7.95], indicating that patients that were treated with anti-PD-1 drugs were at significantly higher risk of developing colitis as compared with those in the control group.

The trace plots (Figures S29–S40) and the posterior density plot (Figure S67) indicate that some Markov chains might have convergence issues when using some priors. The posterior samples of the overall OR might take extreme values in a few MCMC iterations. Caution was advised when interpreting the results to assess their reliability. If more informative priors were available for colitis than the 12 priors considered here, one may further examine whether such priors could improve the MCMC convergence and, thus, the validity of the results.

The Bayesian OR estimates, ranging from 5.89 to 11.37, were much larger than those that were obtained from the frequentist methods. The large difference was likely because the Bayesian methods effectively accounted for the double-zero-event studies, while the conventional frequentist methods could not use such studies.

Besides the four double-zero-event studies, six studies contained zero counts in the control group. Because of the sparse data, the results from the Bayesian methods were fairly sensitive to the choice of hyper-parameters. The 95% CrIs were also much wider than the 95% CIs produced by the frequentist methods, although they still suggested a significant effect of anti-PD-1 drugs on colitis. The frequentist 95% CIs were narrow, likely yielding low coverage probabilities, because the frequentist methods used large-sample properties to derive within-study sample variances and treated them as known, fixed values.

### 3.4. Example 4: Meta-Analysis on Hepatitis

This meta-analysis was from the same systematic review as the previous meta-analysis on colitis [57]; it investigated another adverse event, hepatitis. The data were even sparser in this meta-analysis, which may serve as an excellent example to illustrate the importance of checking MCMC convergence in Bayesian meta-analysis. All k  = 15 studies had zero counts in the control group, and only five studies had non-zero counts in the treatment group. In total, this meta-analysis only had six events among all 7156 patients.

Again, all three frequentist methods yielded $\hat{\tau }\;$ = 0 and produced the overall OR estimate 3.14 with 95% CI [0.76, 12.98], indicating no significant effect of anti-PD-1 drugs on hepatitis. The results that were produced by the Bayesian methods with the considered 12 priors were not reliable, because the trace plots (Figures S41–S52) showed that nearly all Markov chains did not converge. The posterior density plot (Figure S68) indicated multiple modes of the posterior distributions. These issues were likely caused by the extremely sparse data with few events. The estimated heterogeneity standard deviation $\hat{\tau }$ was very sensitive to the choice of priors; it was primarily influenced by the priors, because the sparse data contained little information about heterogeneity. If the researchers had better prior beliefs than the priors used in this article, they may try such alternatives to examine if the inference for this extremely sparse dataset could be improved.

Of note, although the frequentist methods successfully produced the results, as reported in Table 2, they may also be unreliable. For example, the likelihood function for the sparse data may be fairly flat, making the ML algorithm used for deriving the estimates highly unstable [62,63]. The event rate was close/equal to the boundary value 0, and technical issues existed when making inference on the ML estimates.

### 3.5. Example 5: Meta-Analysis on Acute Respiratory Tract Infection

The number of studies (k  = 25) was relatively large in this meta-analysis, and no zero-count appeared in any arm. All three frequentist methods produced nearly the same point and interval estimates for the overall OR, showing that vitamin D supplementation significantly reduced the risk of ARTI. Because of sufficient data information, the results were fairly robust to prior distribution specifications. Across all 12 sets of priors, the overall OR estimate was about 0.82, and its 95% CrI lower and upper bounds were about 0.70 and 0.95, respectively. The trace plots (Figures S53–S64) and posterior density plot (Figure S69) indicate that the Markov chains converged and stabilized well.

## 4. Conclusions

This article has provided a practical review of Bayesian meta-analyses of binary outcomes and summarized several commonly used priors for the heterogeneity variance. We have shown five worked examples to highlight the implementations of incorporating different prior distributions and the importance of checking MCMC convergence. The Bayesian methods are advantageous over the conventional frequentist methods, primarily because they make more practical assumptions and they can incorporate informative priors.

Generally, when the data provided by a meta-analysis are limited (e.g., few studies, small sample sizes, or low event rates), the choice of prior may have a noteworthy impact on meta-analysis results. Ideally, meta-analyses may use priors that represent experts’ opinions. If such priors are unavailable or disputed, sensitivity analyses that use different candidate priors are recommended. In addition, meta-analysts should routinely examine the convergence and stabilization of MCMC processes when performing Bayesian meta-analyses; this is often overlooked in current practice, as illustrated in Examples 3 and 4. When event rates are low and zero event counts appear in many arms in a meta-analysis, the MCMC algorithm may not converge; as such, the inference that is based on posterior samples is unreliable. We have summarized several possible solutions to handle this problem in Figure 1. If the Bayesian meta-analysis still fails to produce valid results after considering these solutions, researchers may refer to several papers that especially focused on the cases of few studies [38,64,65,66,67] and rare events [68,69,70,71,72,73,74,75] for alternative approaches. Moreover, if individual participant data are available, incorporating them into the meta-analysis might help to improve the estimation of treatment effects [13,76,77,78].

Bayesian methods for meta-analysis are not problem-free, and their results must be carefully validated before being applied to decision-making, as discussed above. This article did not intend to promote Bayesian meta-analyses to all situations and involve in the debates on frequentist vs. Bayesian inference. Instead, we aimed at providing some practical guidelines for researchers who are interested in implementing Bayesian meta-analyses. One may refer to other papers that especially aimed at comparing these two types of methods to better understand the pros and cons of frequentist and Bayesian meta-analyses [74,79,80,81,82,83,84].

This article limited the review of Bayesian methods to the case of ORs. Bayesian methods are broadly available for meta-analyses of other effect measures (e.g., relative risks) with binary outcomes and for meta-analyses with other outcomes (e.g., continuous or count data) [42,85]. Similar implementations of Bayesian analyses may be applied to these cases with proper adjustments (e.g., different specifications of likelihood, link functions, and priors).

## Figures and Tables

**Table 1:** Summary of prior distributions for the heterogeneity component (variance $\tau^{2}$ or standard deviation τ ) in a meta-analysis of odds ratios.

Prior Distribution	Used for	Hyper-Parameter
Inverse-gamma, IG(α,β)	$\tau^{2}$	α = β = 0.1; or
		α = β = 0.01; or
		α = β = 0.001.
Uniform, U(0,c)	τ	c = 2; or
		c = 10; or
		c = 100.
Half-normal, $HN\left(0,\sigma^{2}\right)$	τ	$\sigma^{2}$ = 0.1; or
		$\sigma^{2}$ = 1; or
		$\sigma^{2}$ = 2.
Log-normal, $LN\left(\mu ,\sigma^{2}\right)$	$\tau^{2}$	Pharmacological vs. placebo/control comparison: μ = −4.06, σ = 1.45 (all-cause mortality); μ = −3.02, σ = 1.85 (semi-objective outcome); μ = −2.13, σ = 1.58 (subjective outcome). Pharmacological vs. pharmacological comparison: μ = −4.27, σ = 1.48 (all-cause mortality); μ = −3.23, σ = 1.88 (semi-objective outcome); μ = −2.34, σ = 1.62 (subjective outcome). Non-pharmacological comparison: μ = −3.93, σ = 1.51 (all-cause mortality); μ = −2.89, σ = 1.91 (semi-objective outcome); μ = −2.01, σ = 1.64 (subjective outcome).

**Table 2:** The estimated odds ratios (OR) and heterogeneity standard deviations (Tau) with their 95% credible/confidence intervals using different methods in the five examples.

	Bayesian Method												Frequentist Method ^e^		
Estimate	Inverse-Gamma Prior ^a^			Uniform Prior ^b^			Half-Normal Prior ^c^			Log-Normal Prior ^d^			DL	ML	REML
	IG1	IG2	IG3	U1	U2	U3	HN1	HN2	HN3	LN1	LN2	LN3			
Example 1: meta-analysis on stillbirth															
OR	4.30 (2.95, 5.86)	4.30 (2.95, 5.87)	4.26 (2.90, 5.90)	4.26 (2.85, 5.95)	4.25 (2.85, 5.94)	4.25 (2.85, 5.95)	4.35 (3.13, 5.78)	4.26 (2.89, 5.91)	4.26 (2.86, 5.92)	4.39 (3.16, 5.76)	*4.33* *(3.04, 5.82)*	4.31 (3.01, 5.83)	4.59 (3.56, 5.93)	4.52 (3.42, 5.96)	4.47 (3.34, 5.99)
Tau	0.49 (0.29, 0.89)	0.50 (0.30, 0.89)	0.52 (0.32, 0.91)	0.53 (0.31, 0.98)	0.54 (0.31, 0.98)	0.54 (0.31, 0.98)	0.45 (0.28, 0.71)	0.52 (0.31, 0.93)	0.53 (0.31, 0.95)	0.43 (0.26, 0.72)	*0.47* *(0.28, 0.80)*	0.48 (0.29, 0.82)	0.38 (0.28, 0.91)	0.43 (0.28, 0.91)	0.46 (0.28, 0.91)
Example 2: meta-analysis on patient enrollment in clinical trials															
OR	1.17 (0.99, 1.39)	1.17 (0.95, 1.44)	1.17 (0.87, 1.57)	1.17 (0.96, 1.43)	1.17 (0.96, 1.43)	1.17 (0.96, 1.43)	1.17 (0.98, 1.41)	1.17 (0.96, 1.42)	1.17 (0.98, 1.41)	1.17 (0.99, 1.39)	*1.17* *(0.97, 1.41)*	1.17 (0.95, 1.45)	1.16 (1.03, 1.30)	1.16 (1.03, 1.30)	1.16 (1.03, 1.30)
Tau	0.09 (0.02, 0.34)	0.15 (0.06, 0.42)	0.29 (0.16, 0.63)	0.11 (0.01, 0.46)	0.12 (0.01, 0.47)	0.11 (0.01, 0.47)	0.10 (0.01, 0.36)	0.10 (0.01, 0.44)	0.11 (0.00, 0.45)	0.10 (0.03, 0.28)	*0.12* *(0.03, 0.36)*	0.16 (0.05, 0.43)	0.00 (0.00, 0.46)	0.00 (0.00, 0.46)	0.00 (0.00, 0.46)
Example 3: meta-analysis on colitis^f^															
OR	6.90 (2.28, 102)	7.15 (2.25, 84.30)	7.99 (2.25, 141)	7.59 (2.24, 47.85)	9.83 (2.24, 2008)	11.37 (2.25, 10^5^)	6.09 (2.23, 22.46)	6.88 (2.23, 35.32)	7.62 (2.28, 56.53)	5.89 (2.25, 21.04)	5.95 (2.18, 23.08)	*6.34* *(2.20, 26.20)*	3.39 (1.45, 7.95)	3.39 (1.45, 7.95)	3.39 (1.45, 7.95)
Tau	0.25 (0.03, 3.89)	0.44 (0.08, 3.37)	0.73 (0.22, 3.98)	0.74 (0.03, 1.90)	1.13 (0.05, 7.32)	1.33 (0.05, 12.84)	0.20 (0.01, 0.68)	0.51 (0.02, 1.78)	0.66 (0.03, 2.45)	0.13 (0.03, 0.49)	0.21 (0.04, 1.02)	*0.31* *(0.07, 1.26)*	0.00 (0.00, 0.00)	0.00 (0.00, 0.00)	0.00 (0.00, 0.00)
Example 4: meta-analysis on hepatitis^g^															
OR	NA	NA	NA	NA	NA	NA	NA	NA	NA	NA	NA	NA	3.14 (0.76, 12.98)	3.14 (0.76, 12.98)	3.14 (0.76, 12.98)
Tau	NA	NA	NA	NA	NA	NA	NA	NA	NA	NA	NA	NA	0.00 (0.00, 0.00)	0.00 (0.00, 0.00)	0.00 (0.00, 0.00)
Example 5: meta-analysis on acute respiratory tract infection															
OR	0.83 (0.70, 0.95)	0.82 (0.69, 0.95)	0.81 (0.67, 0.95)	0.82 (0.68, 0.95)	0.82 (0.68, 0.95)	0.82 (0.68, 0.95)	0.82 (0.69, 0.95)	0.82 (0.69, 0.95)	0.82 (0.69, 0.95)	0.83 (0.71, 0.95)	0.83 (0.70, 0.95)	*0.82* *(0.70, 0.95)*	0.83 (0.72, 0.95)	0.83 (0.72, 0.95)	0.83 (0.71, 0.95)
Tau	0.21 (0.05, 0.41)	0.23 (0.10, 0.43)	0.29 (0.18, 0.48)	0.25 (0.08, 0.46)	0.25 (0.08, 0.45)	0.25 (0.08, 0.46)	0.23 (0.07, 0.41)	0.25 (0.08, 0.45)	0.25 (0.08, 0.45)	0.19 (0.06, 0.36)	0.22 (0.08, 0.40)	*0.24* *(0.10, 0.42)*	0.21 (0.08, 0.47)	0.21 (0.08, 0.47)	0.23 (0.08, 0.47)

^a^ Inverse-gamma priors for $\tau^{2}$ with three sets of hyper-parameters: IG1, IG(0.001, 0.001); IG2, IG(0.01, 0.01); IG3, IG(0.1, 0.1). ^b^ Uniform priors for τ with three sets of hyper-parameters: U1, U(0, 2); U2, U(0, 10); U3, U(0, 100). ^c^ Half-normal priors for τ with three sets of hyper-parameters: HN1, HN(0, 0.1); HN2, HN(0, 1); HN3, HN(0, 2). ^d^ Log-normal priors for $\tau^{2}$ with three sets of hyper-parameters. For the meta-analyses on stillbirth and on patient enrollment in clinical trials (non-pharmacological treatment comparisons), they are: LN1, $LN\left(-3.93,\;1.51^{2}\right)$; LN2, $LN\left(-2.89,\;1.91^{2}\right)$; LN3, $LN\left(-2.01,\;1.64^{2}\right)$. For the meta-analyses on colitis, on hepatitis, and on acute respiratory tract infection (pharmacological treatments vs. placebo/control), they are: LN1, $LN\left(-4.06,\;1.45^{2}\right)$; LN2, $LN\left(-3.02,\;1.85^{2}\right)$; LN3, $LN\left(-2.13,\;1.58^{2}\right)$. The results in italic are based on the set of hyper-parameters that best matches the outcome categorization (all-cause mortality, semi-objective, and subjective outcomes) according to Turner et al. [54] ^e^ The frequentist methods include the DerSimonian–Laird (DL), maximum likelihood (ML), and restricted maximum likelihood (REML) estimators. ^f^Markov chains might have convergence issues when using some priors, so the results may be interpreted with cautions. ^g^Markov chains had poor convergence when using all priors, so the results by the Bayesian methods may not be reliable and are reported as not available (NA). The results by the frequentist methods should also be interpreted with great caution.

## Data Availability

The forest plots in Figure 2 and Figures S1–S4 (Supplementary Materials data C) include the data used in this article.

## Supplementary Materials

The following are available online at https://www.mdpi.com/article/10.3390/ijerph18073492/s1, In the Supplementary Materials, Supplementary Materials data A presents conventional frequentist meta-analysis of odds ratios, Supplementary Materials data B gives R code for analyzing the five examples, Supplementary Materials data C presents forest plots, Supplementary Materials data D presents trace plots, and Supplementary Materials data E presents posterior density plots.
