# Supplementary material for: Bayesian Methods for Meta-Analyses of Binary Outcomes: Implementations, Examples, and Impact of Priors
Source: Int J Environ Res Public Health. 2021 Mar 27;18(7):3492. doi: 10.3390/ijerph18073492 (PMC8036799; doi:10.3390/ijerph18073492)
Supplement: Supplementary file 1 [file ijerph-18-03492-s001.pdf]

Supplementary Materials for
"Bayesian methods for meta-analyses of binary outcomes: implementations, examples, and impact of priors"

List of contents

Appendix A. Conventional frequentist meta-analysis of odds ratios

Appendix B. R code for analyzing the five examples

Appendix C. Forest plots

Appendix D. Trace plots

Appendix E. Posterior density plots

Appendix F. References

A. Conventional frequentist meta-analysis of odds ratios

A.1. Conventional meta-analysis models

The fixed-effect (FE) and random-effects (RE) models are two conventional approaches for meta-analyses. Each model employs different assumptions to synthesize multiple studies. Suppose that a meta-analysis contains k statistically independent studies; each has an underlying true effect size, θ_i ($i = 1, \ldots, k$). The FE model assumes that all studies have a common true effect size; that is, $\theta_i = \theta$. The only source of variation among effects is attributed to sampling errors within studies. Let y_i be the observed effect size of study i, whose within-study variance σ_i^2 is often treated as a known, fixed value. The FE model is specified as:

$y_i = \theta + \varepsilon_i$, $\varepsilon_i \sim N(0, \sigma_i^2)$,

where ε_i represents the sampling error.

In practice, the FE model assumptions may not be valid, as different studies are often expected to be heterogeneous due to the difference in their patients' basic characteristics, study methods, research teams, and other potential confounders.[1] In such cases, the RE model is more appropriate as it assumes that the studies have different underlying true effect sizes θ_i. Consequently, the observed effect sizes are subject to two sources of variation: variances due to sampling error σ_i^2 within studies and the between-studies variance τ^2. Specifically, the RE model is

$y_i = \theta_i + \varepsilon_i$, $\varepsilon_i \sim N(0, \sigma_i^2)$;

$\theta_i \sim N(\theta, \tau^2)$.

The θ_i's are assumed to be random effects, following a normal distribution with mean θ and variance τ^2. Thus, θ now represents the overall effect size and is of primary interest. When $\tau^2 = 0$, the RE model reduces to the FE model. The RE model accounts for the additional source of variation between studies, thus being viewed as more conservative than the FE model; it often produces wider confidence intervals (CIs).

A.2. Frequentist approach

Currently, most meta-analyses are implemented using frequentist methods. The FE estimate of θ is the inverse-of-variance weighted least squares average; specifically,

$\hat{\theta} = \sum_{i=1}^{k} w_i y_i / \sum_{i=1}^{k} w_i$,

with standard error

$\mathrm{SE}(\hat{\theta}) = \left(\sum_{i=1}^{k} w_i\right)^{-1/2}$,

where $w_i = 1/\sigma_i^2$ is the weight for study i. Using the RE model approach, study-specific weights are revised to incorporate an estimate of the between-studies variance, $\hat{\tau}^2$. Thus, the RE estimate of θ and its variance are obtained by replacing the FE weights w_i with $w_i^* = 1/(\sigma_i^2 + \hat{\tau}^2)$.

Various methods are available to estimate the between-studies variance.[2-4] If $\hat{\tau}^2$ reduces to 0, the RE estimate becomes the FE estimate. Conversely, if $\hat{\tau}^2 \to \infty$, the RE estimate becomes the naïve arithmetic mean. This article primarily considers three commonly-used estimators of τ^2: DerSimonian–Laird (DL) estimator, $\hat{\tau}_{\mathrm{DL}}^2$, based on the method of moments,[5] maximum-likelihood (ML) estimator, $\hat{\tau}_{\mathrm{ML}}^2$, and restricted maximum-likelihood (REML) estimator, $\hat{\tau}_{\mathrm{REML}}^2$.[6]

The DL estimator ($\hat{\tau}^2_{\mathrm{DL}}$) is a non-iterative method and can be easily calculated. However, it can produce zero estimates and yields considerable bias when the meta-analysis contains a small number of studies.[3] Specifically, this estimator is calculated as $\hat{\tau}^2_{\mathrm{DL}} = \max\{0, [Q-(k-1)]/\left(\sum_{i=1}^k w_i - \sum_{i=1}^k w_i^2/\sum_{i=1}^k w_i\right)\}$.
Here, $Q = \sum_{i=1}^k w_i\left(y_i - \hat{\theta}\right)^2$ is the conventional test statistic for heterogeneity, where $\hat{\theta}$ and w_i are the estimated overall effect size and study-specific weights under the FE setting, respectively.

The ML estimator ($\hat{\tau}^2_{\mathrm{ML}}$) is a classic method that has many desired statistical properties. The estimator is obtained by maximizing the log-likelihood of the RE model:
$$\log L \propto -\frac{1}{2}\sum_{i=1}^k \left[\log(\sigma_i^2+\tau^2) + \frac{(y_i-\theta)^2}{\sigma_i^2+\tau^2}\right],$$
with respect to both θ and τ^2. Nevertheless, it is well known that ML estimates of variance components are often biased.[7] As an alternative, the REML method is able to produce unbiased variance estimates. More specifically, the REML approach maximizes the following modified log-likelihood:[6]
$$\log L \propto -\frac{1}{2}\sum_{i=1}^k \left[\log(\sigma_i^2+\tau^2) + \frac{\left(y_i-\hat{\theta}\right)^2}{\sigma_i^2+\tau^2}\right] - \frac{1}{2}\log\sum_{i=1}^k \frac{1}{\sigma_i^2+\tau^2},$$
with respect to τ^2, where $\hat{\theta} = \frac{\sum_{i=1}^k y_i/(\sigma_i^2+\tau^2)}{\sum_{i=1}^k 1/(\sigma_i^2+\tau^2)}$.

Both the ML and REML methods require iterative computations, which may not converge in some cases (e.g., k is small).[8] Based on previous simulation studies, the REML estimator is generally recommended.[2,3,9] The various estimators can be obtained using many software packages, such as the R packages “metafor” and “meta”.[8,10]

A.3. Meta-analysis of odds ratio with a worked example

Many meta-analyses include studies with binary outcomes (e.g., disease incidence, mortality, pass/fail), and the odds ratio (OR) is commonly used as the effect measure to quantify treatment effects.[11] We will focus on such meta-analyses of ORs.

Table S1 illustrates a 2×2 table of an individual study with a binary outcome, which is typically reported in published articles. Within Table S1, we provide counts of both events and no events in both the treatment and control groups. Let r represent event counts and n be sample sizes. Furthermore, let subscripts T and C denote the treatment and control groups, respectively. The OR is estimated as $\widehat{\mathrm{OR}} = \frac{r_\mathrm{T}/(n_\mathrm{T}-r_\mathrm{T})}{r_\mathrm{C}/(n_\mathrm{C}-r_\mathrm{C})}$. In practice, the OR is often analyzed on a logarithmic scale. The rationale for this decision is the log OR, $y = \log\left(\widehat{\mathrm{OR}}\right)$, has an asymptotic normal distribution when the counts in the 2×2 table (e.g., Table S1) are sufficiently large. The sample variance of the log OR is approximated as $\sigma^2 = r_\mathrm{T}^{-1} + (n_\mathrm{T}-r_\mathrm{T})^{-1} + r_\mathrm{C}^{-1} + (n_\mathrm{C}-r_\mathrm{C})^{-1}$. The y and σ^2 correspond to the individual study summary data. In the context of meta-analysis, as shown above, we would include the subscript i to index a specific study from k studies.

Table S1. Illustration of a 2×2 table.

	Treatment Group	Control Group	Total
Event	r_T	r_C	$r_\mathrm{T}+r_\mathrm{C}$
No Event	$n_\mathrm{T}-r_\mathrm{T}$	$n_\mathrm{C}-r_\mathrm{C}$	$n_\mathrm{T}+n_\mathrm{C}-r_\mathrm{T}-r_\mathrm{C}$
Total	n_T	n_C	$n_\mathrm{T}+n_\mathrm{C}$

For example, Lamont et al.[12] collected 13 cohort studies that compared the risk of stillbirth recurrence in women who had experienced a previous stillbirth with those who had a previous live birth. Figure 1 in the main content shows the associated forest plot. The I^2 index was 84%, suggesting considerable heterogeneity beyond that of sampling error.[13] Compared with women with a previous live birth, women with a previous stillbirth had a significantly increased risk of stillbirth recurrence with an overall OR=4.59 and 95% CI [3.56, 5.93]. These results used the DL estimator of between-studies variance.

A potential problem in meta-analyses of binary outcomes is the presence of zero counts. Using conventional meta-analysis methods, ORs are not estimable in studies with zero event counts in both arms; such studies are usually excluded from meta-analyses.[14] However, an exclusion of this sort may lose important information and lead to research waste.[15-17] When only one arm in a study contains zero count, a continuity correction (typically 0.5) can be applied to the 2×2 table, so that the OR and its CI can be calculated.

The problem of zero counts may be avoided by using more advanced meta-analysis methods, such as generalized linear mixed models, which are considered one-stage approaches completed without calculating ORs for individual studies.[18] Nevertheless, such methods may require comparatively complicated computation algorithms, with the iterative estimation procedure being unstable in certain instances. Alternatively, Bayesian methods offer a flexible approach with the aim at the same purpose. Within a Bayesian framework for meta-analysis, researchers are able incorporate informative priors to improve estimated treatment effects.

B. R code for analyzing the five examples

```
## load libraries
library("rjags")
library("coda")

################################################################
## functions

ma <- function(prior.distribution){
  if (prior.distribution == "IG") {
    out <- "model{
    for(i in 1:n.studies){
      delta[i,1] <- 0
      mu[i] ~ dnorm(0, 0.0001)  # vague priors for trial baselines
      for(k in 1:2){
        r[i,k] ~ dbin(p[i,k], n[i,k])  # binomial likelihood
        logit(p[i,k]) <- mu[i] + delta[i,k]
      }
      delta[i,2] ~ dnorm(lor, prec)  # trial-specific LOR distributions
    }
    lor ~ dnorm(0, 0.0001)  #  vague priors for log odds ratio
    # inverse gamma prior
    tau2 <- 1/prec
    tau <- sqrt(1/prec)
    prec ~ dgamma(alpha, beta)
    OR <- exp(lor)
  }" }
  else if (prior.distribution == "U") {
    out <- "model{
    for(i in 1:n.studies){
      delta[i,1] <- 0
      mu[i] ~ dnorm(0, 0.0001)  # vague priors for trial baselines
      for(k in 1:2){
        r[i,k] ~ dbin(p[i,k], n[i,k])  # binomial likelihood
        logit(p[i,k]) <- mu[i] + delta[i,k]
      }
      delta[i,2] ~ dnorm(lor, prec)  # trial-specific LOR distributions
    }
    lor ~ dnorm(0, 0.0001)  #  vague priors for log odds ratio
    # uniform prior
    tau2 <- tau*tau
    prec <- 1/tau2
    tau ~ dunif(alpha, beta)
    OR <- exp(lor)
  }" }
  else if (prior.distribution == "HN") {
    out <- "model{
    for(i in 1:n.studies){
      delta[i,1] <- 0
      mu[i] ~ dnorm(0, 0.0001)  # vague priors for trial baselines
      for(k in 1:2){
        r[i,k] ~ dbin(p[i,k], n[i,k])  # binomial likelihood
        logit(p[i,k]) <- mu[i] + delta[i,k]
      }
      delta[i,2] ~ dnorm(lor, prec)  # trial-specific LOR distributions
    }
    lor ~ dnorm(0, 0.0001)  #  vague priors for log odds ratio
    # half-mormal prior
    tau2 <- tau*tau
    prec <- 1/tau2
    tau ~ dnorm(alpha, 1/beta) T(0,)
    OR <- exp(lor)
```

```
  }" }
  else if (prior.distribution == "LN") {
    out <- "model{
    for(i in 1:n.studies){
      delta[i,1] <- 0
      mu[i] ~ dnorm(0, 0.0001)  # vague priors for trial baselines
      for(k in 1:2){
        r[i,k] ~ dbin(p[i,k], n[i,k])  # binomial likelihood
        logit(p[i,k]) <- mu[i] + delta[i,k]
      }
      delta[i,2] ~ dnorm(lor, prec)  # trial-specific LOR distributions
    }
    lor ~ dnorm(0, 0.0001)  #  vague priors for log odds ratio
    # log-normal prior
    prec <- 1/tau2
    tau <- sqrt(1/prec)
    tau2 ~ dlnorm(alpha, 1/(beta)^2)
    OR <- exp(lor)
  }" }
  else {
    print("Check prior distribution")
  }
  return(out)
}


prior_results <- function(data, prior, alpha, beta,
                          n.burnin = 50000, n.iter = 200000, n.chains = 3,
                          n.adapt = 1000, thin = 2, seed = 1234){
  dat.jags <- list(n.studies = length(data$r1), alpha = alpha, beta = beta,
    r = cbind(data$r1,data$r2), n = cbind(data$n1, data$n2))
  inits <- list(
    list(lor = 0, mu = rep(0, length(data$r1)),
         .RNG.name = "base::Wichmann-Hill", .RNG.seed = 1234),
    list(lor = -1, mu = rep(1, length(data$r1)),
         .RNG.name = "base::Wichmann-Hill", .RNG.seed = 12345),
    list(lor = 1, mu = rep(-1, length(data$r1)),
         .RNG.name = "base::Wichmann-Hill", .RNG.seed = 123456))

  params <- c("OR", "lor", "prec", "tau", "tau2")
  set.seed(seed)
  jags.ma <- jags.model(file = textConnection(ma(prior)), data = dat.jags,
    n.chains = n.chains, n.adapt = n.adapt, inits = inits)
  update(jags.ma, n.iter = n.burnin)
  coda.ma <- coda.samples(model = jags.ma, variable.names = params,
    n.iter = n.iter, thin = thin)
  smry.ma <- summary(coda.ma)
  print(smry.ma$quantiles[c("OR", "tau") , c("2.5%", "50%", "97.5%")])
  out <- smry.ma$quantiles[ , c("2.5%", "50%", "97.5%")]
  colname <- c("lb", "median", "ub")
  write.table(matrix(out, byrow = FALSE, nrow = 5), file = "summary1_MA.txt",
              row.names = c("OR", "lor", "prec", "tau", "tau2"),
              col.names = colname)

  # trace plot of log OR
  png(paste0(paste("traceplot_", prior), "_lor", ".png"),
      res = 600, height = 8.5, width = 11, units = "in")
  par(mfcol = c(length(coda.ma), 1))
  for(k in 1:length(coda.ma)){
    temp <- as.vector(coda.ma[[k]][,"lor"])
    plot(temp, type = "l", col = "red", cex.lab = 1.5, cex.main = 1.5,
         xlab = "Iteration", ylab = "Log odds ratio",
         main = paste("Chain", k))
```

```
  }
  dev.off()

  # density plot of log OR
  png(paste0("densityplot", "lor", ".png"),
      res = 600, height = 8.5, width = 11, units = "in")
  post.coda <- NULL
  for(k in 1:length(coda.ma)){
     post.coda <- rbind(post.coda, coda.ma[[k]][,"lor"])
  }
  postden <- density(post.coda)
  plot(postden, main = "Posterior density", xlab = "Log odds ratio")
  polygon(postden, col = "lightblue", border = "darkblue")
  dev.off()
}


################################################################
## data of five real-world meta-analyses

Stillbirth <- list(r1 = c(20, 1884, 1402, 179, 1144, 257, 1832, 1309,
                          3407, 145, 660, 803, 477),
                   r2 = c(2, 45, 18, 5, 72, 5, 106, 50, 222, 3, 21, 12, 13),
                   n1 = c(3160, 402201, 524328, 33715, 242672, 70942, 533258,
                          306627, 568315, 51762, 242881, 250769, 144565),
                   n2 = c(316, 1979, 2363, 364, 2168, 373, 3161, 2677, 5996,
                          348, 1323, 2058, 872))

PPI <- list(r1 = c(10, 66, 20, 27, 522, 31, 31, 95),
            r2 = c(16, 62, 14, 43, 570, 63, 63, 116),
            n1 = c(63, 87, 194, 682, 1381, 1149, 1149, 3297),
            n2 = c(63, 86, 146, 682, 1412, 2301, 2301, 2949))

Colitis <- list(r1 = c(2, 0, 0, 0, 0, 0, 0, 0, 1, 1, 0, 0, 0),
                r2 = c(6, 2, 0, 2, 0, 3, 0, 3, 2, 2, 2, 1, 0),
                n1 = c(255, 268, 129, 135, 309, 309, 62, 151, 171, 171, 578,
                       205, 102),
                n2 = c(266, 287, 131, 142, 343, 339, 59, 154, 179, 178, 609,
                       206, 268))

Hepatitis <-  list(r1 = c(0, 0, 0, 0, 0, 0, 0, 0, 0, 0, 0, 0, 0, 0, 0),
                  r2 = c(0, 0, 0, 1, 0, 1, 1, 0, 0, 0, 1, 0, 2, 0, 0),
                  n1 = c(255, 268, 129, 135, 111, 309, 309, 62, 397, 151,
                         171, 171, 578, 205, 102),
                  n2 = c(266, 287, 131, 142, 236, 343, 339, 59, 406, 154,
                         179, 178, 609, 206, 268))

ARTI <- list(r1 = c(33, 69, 126, 54, 11, 458, 29, 245, 53, 155, 39, 38, 276,
                    96, 80, 17, 53, 75, 93, 58, 10, 93, 5, 24, 14),
             r2 = c(32, 68, 97, 39, 4, 438, 30, 260, 44, 154, 26, 26, 303,
                    185, 70, 32, 94, 76, 85, 83, 10, 110, 4, 17, 16),
             n1 = c(76, 167, 229, 84, 24, 1030, 89, 1505, 103, 161, 62, 58,
                    360, 197, 234, 99, 80, 118, 125, 103, 11, 207, 35, 52, 16),
             n2 = c(81, 167, 224, 80, 24, 1034, 86, 1506, 141, 161, 62, 58,
                    399, 397, 258, 148, 156, 122, 125, 137, 14, 201, 54, 55, 18))


################################################################
## Bayesian meta-analysis for each dataset

data <- Stillbirth # PPI, Colitis, Hepatitis, or ARTI

prior_results(data = data, prior = "IG", alpha = 0.001, beta = 0.001)
```

```
prior_results(data = data, prior = "IG", alpha = 0.01, beta = 0.01)
prior_results(data = data, prior = "IG", alpha = 0.1, beta = 0.1)

prior_results(data = data, prior = "U", alpha = 0, beta = 2)
prior_results(data = data, prior = "U", alpha = 0, beta = 10)
prior_results(data = data, prior = "U", alpha = 0, beta = 100)

prior_results(data = data, prior = "HN", alpha = 0, beta = 0.1)
prior_results(data = data, prior = "HN", alpha = 0, beta = 1)
prior_results(data = data, prior = "HN", alpha = 0, beta = 2)

# for Stillbirth and PPI
prior_results(data = data, prior = "LN", alpha = -3.93, beta = 1.51)
prior_results(data = data, prior = "LN", alpha = -2.89, beta = 1.91)
prior_results(data = data, prior = "LN", alpha = -2.01, beta = 1.64)

# for Colitis, Hepatitis, and ARTI
prior_results(data = data, prior = "LN", alpha = -4.06, beta = 1.45)
prior_results(data = data, prior = "LN", alpha = -3.02, beta = 1.85)
prior_results(data = data, prior = "LN", alpha = -2.13, beta = 1.58)
```

C. Forest plots

This section presents the forest plots of the meta-analyses on patient enrollment in clinical trials (Example 2), on colitis (Example 3), on hepatitis (Example 4), and on acute respiratory tract infection (Example 5). In each forest plot, the ORs and their 95% CIs were calculated using the frequentist methods described in Appendix A.3.

	PPI		No PPI			
Author(s) and year	Events	Sample size	Events	Sample size	OR	[95% CI]
(1) Du 2008	16	63	10	63	1.80	[0.75; 4.36]
(2) Hutchison 2007	62	86	66	87	0.82	[0.42; 1.62]
(3) Dear 2012	14	146	20	194	0.92	[0.45; 1.89]
(4) Man 2015	43	682	27	682	1.63	[1.00; 2.67]
(5) Guarino 2006	570	1412	522	1381	1.11	[0.96; 1.30]
(6) Cockayne 2017a	63	2301	31	1149	1.02	[0.66; 1.57]
(7) Cockayne 2017b	63	2301	31	1149	1.02	[0.66; 1.57]
(8) Ford 2004	116	2949	95	3297	1.38	[1.05; 1.82]

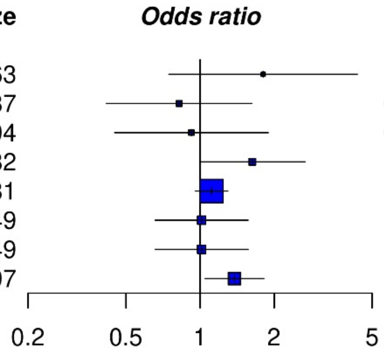

Figure S1. Forest plot of the meta-analysis on patient enrollment in clinical trials (Example 2).

Author(s)	Intervention Events	Intervention Sample size	Control Events	Control Sample size	Odds ratio	[95% CI]
(1) Bellmunt	6	266	2	255	2.92	[0.58; 14.60]
(2) Borghaei	2	287	0	268	4.70	[0.22; 98.39]
(3) Brahmer	0	131	0	129		
(4) Fehrenbacher	2	142	0	135	4.82	[0.23; 101.36]
(5) Herbst 10 mg/kg	0	343	0	309		
(6) Herbst 2 mg/kg	3	339	0	309	6.44	[0.33; 125.15]
(7) Langer	0	59	0	62		
(8) Reck	3	154	0	151	7.00	[0.36; 136.68]
(9) Ribas 10 mg/kg	2	179	1	171	1.92	[0.17; 21.38]
(10) Ribas 2 mg/kg	2	178	1	171	1.93	[0.17; 21.50]
(11) Rittmeyer	2	609	0	578	4.76	[0.23; 99.39]
(12) Robert	1	206	0	205	3.00	[0.12; 74.07]
(13) Weber	0	268	0	102		

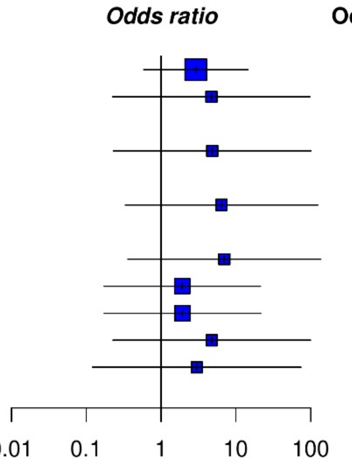

Figure S2. Forest plot of the meta-analysis on colitis (Example 3).

Author(s)	Intervention Events	Intervention Sample size	Control Events	Control Sample size	Odds ratio	[95% CI]
(1) Bellmunt	0	266	0	255		
(2) Borghaei	0	287	0	268		
(3) Brahmer	0	131	0	129		
(4) Fehrenbacher	1	142	0	135	2.87	[0.12; 71.13]
(5) Ferris	0	236	0	111		
(6) Herbst 10 mg/kg	1	343	0	309	2.71	[0.11; 66.79]
(7) Herbst 2 mg/kg	1	339	0	309	2.74	[0.11; 67.58]
(8) Langer	0	59	0	62		
(9) Motzer	0	406	0	397		
(10) Reck	0	154	0	151		
(11) Ribas 10 mg/kg	1	179	0	171	2.88	[0.12; 71.24]
(12) Ribas 2 mg/kg	0	178	0	171		
(13) Rittmeyer	2	609	0	578	4.76	[0.23; 99.39]
(14) Robert	0	206	0	205		
(15) Weber	0	268	0	102		

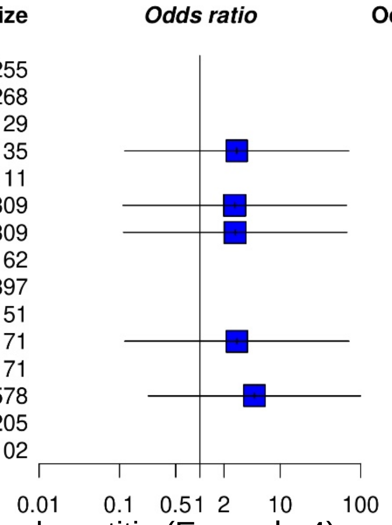

Figure S3. Forest plot of the meta-analysis on hepatitis (Example 4).

Author(s) and year	Intervention Events	Intervention Sample size	Control Events	Control Sample size	Odds ratio [95% CI]
(1) Li−Ng 2009	32	81	33	76	0.85 [0.45; 1.61]
(2) Urashima 2010	68	167	69	167	0.98 [0.63; 1.51]
(3) Manaseki−Holland 2010	97	224	126	229	0.62 [0.43; 0.90]
(4) Laaksi 2010	39	80	54	84	0.53 [0.28; 0.99]
(5) Majak 2010	4	24	11	24	0.24 [0.06; 0.90]
(6) Trilok−Kumar 2011	438	1034	458	1030	0.92 [0.77; 1.09]
(7) Lehouch 2012	30	86	29	89	1.11 [0.59; 2.07]
(8) Manaseki−Holland 2012	260	1506	245	1505	1.07 [0.89; 1.30]
(9) Camargo 2012	44	141	53	103	0.43 [0.25; 0.72]
(10) Murdoch 2012	154	161	155	161	0.85 [0.28; 2.59]
(11) Bergman 2012	26	62	39	62	0.43 [0.21; 0.88]
(12) Marchisio 2013	26	58	38	58	0.43 [0.20; 0.90]
(13) Rees 2013	303	399	276	360	0.96 [0.69; 1.34]
(14) Tran 2014	185	397	96	197	0.92 [0.65; 1.29]
(15) Goodall 2014	70	258	80	234	0.72 [0.49; 1.05]
(16) Urashima 2014	32	148	17	99	1.33 [0.69; 2.56]
(17) Grant 2014	94	156	53	80	0.77 [0.44; 1.36]
(18) Martineau 2015 (ViDiCO)	76	122	75	118	0.95 [0.56; 1.60]
(19) Martineau 2015 (ViDiAs)	85	125	93	125	0.73 [0.42; 1.27]
(20) Martineau 2015 (ViDiFlu)	83	137	58	103	1.19 [0.71; 2.00]
(21) Dubnov−Raz 2015	10	14	10	11	0.25 [0.02; 2.65]
(22) Denlinger 2016	110	201	93	207	1.48 [1.00; 2.19]
(23) Tachimoto 2016	4	54	5	35	0.48 [0.12; 1.93]
(24) Ginde 2016	17	55	24	52	0.52 [0.24; 1.15]
(25) Simpson 2015	16	18	14	16	1.14 [0.14; 9.21]

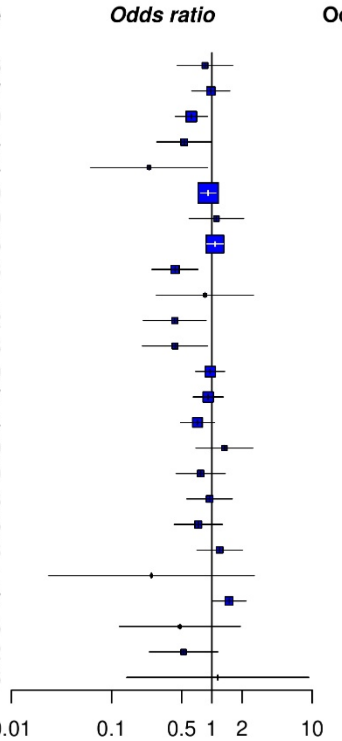

Figure S4. Forest plot of the meta-analysis on acute respiratory tract infection (Example 5).

D. Trace plots

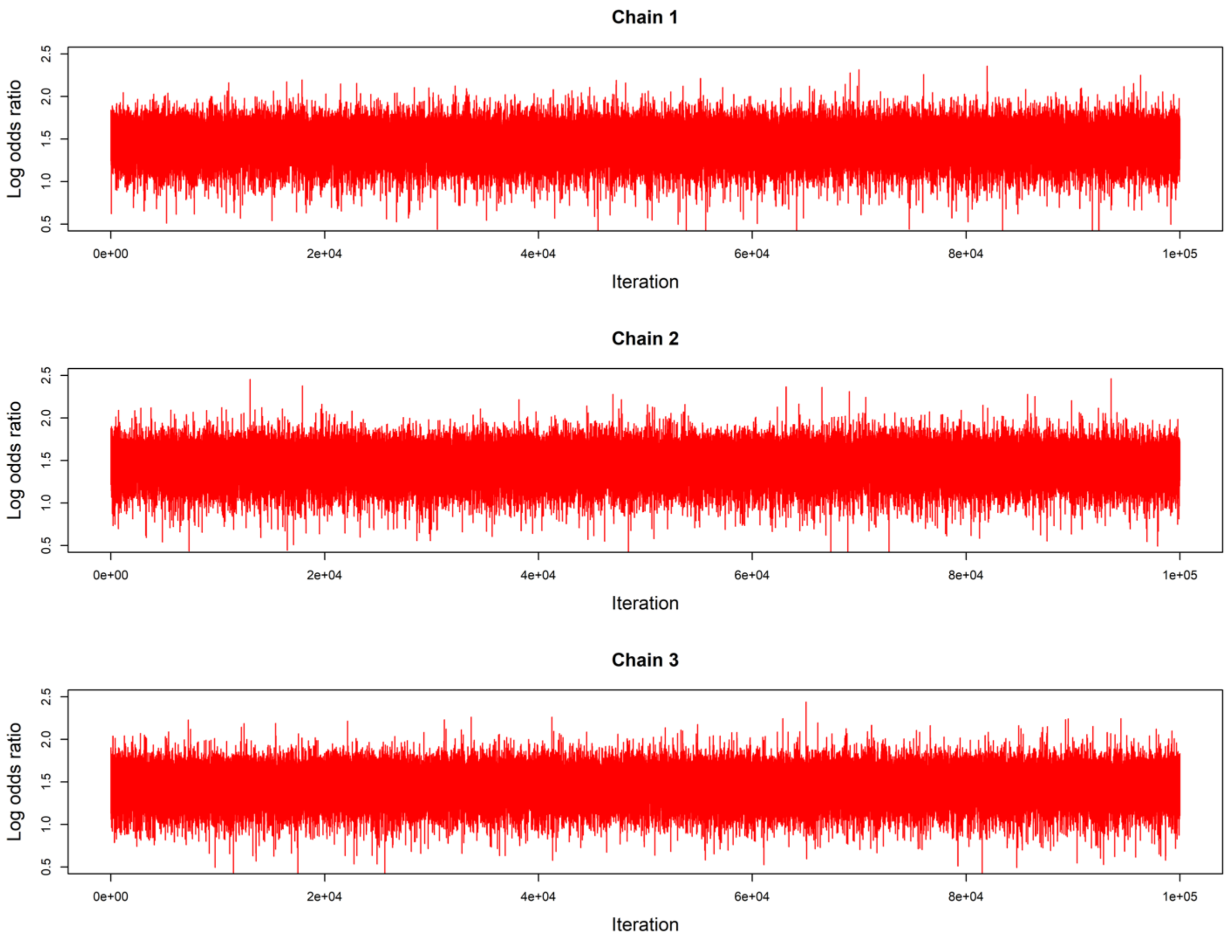

Figure S5. Trace plots of the overall log odds ratio based on the prior $IG(0.001, 0.001)$ for τ^2 in the meta-analysis on stillbirth (Example 1).

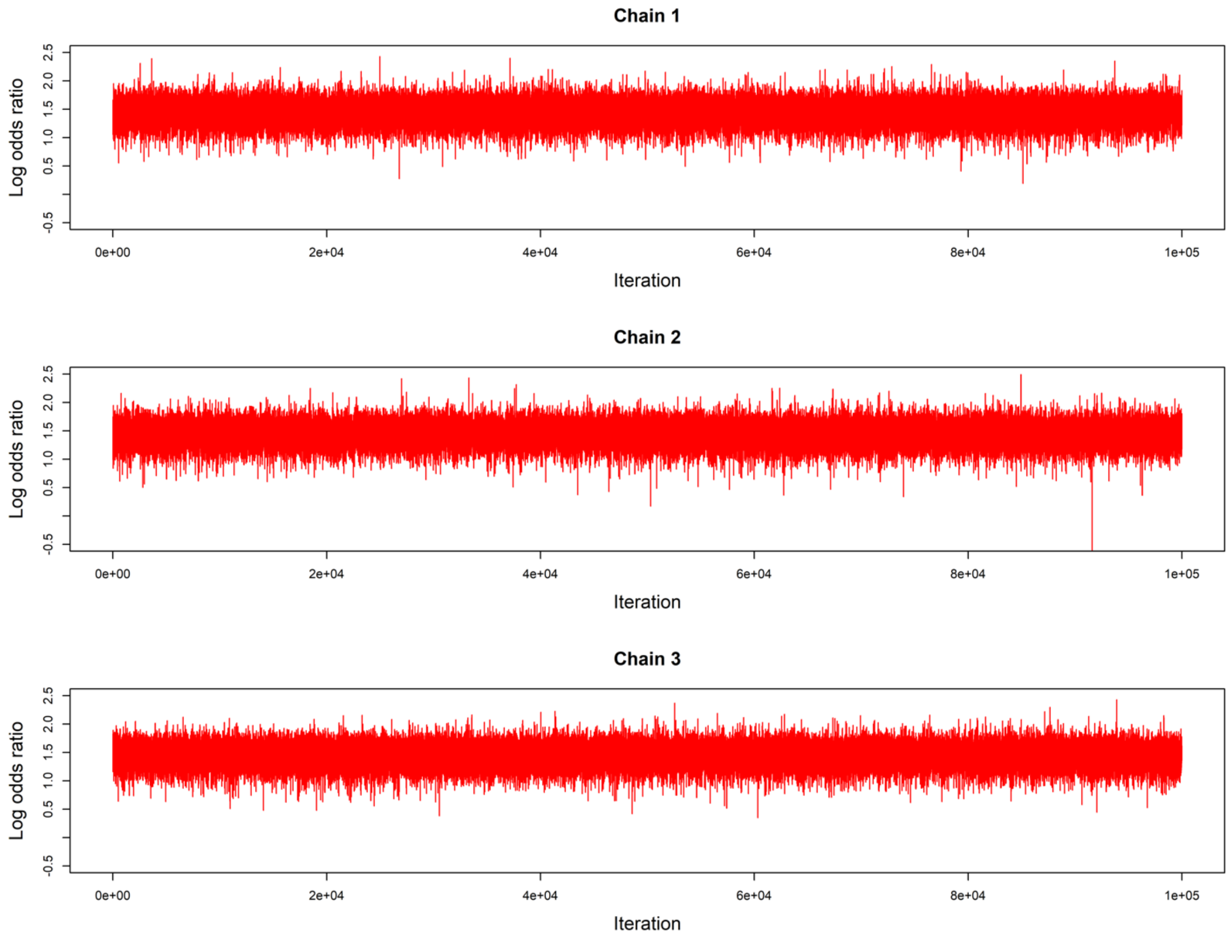

Figure S6. Trace plots of the overall log odds ratio based on the prior $IG(0.01, 0.01)$ for τ^2 in the meta-analysis on stillbirth (Example 1).

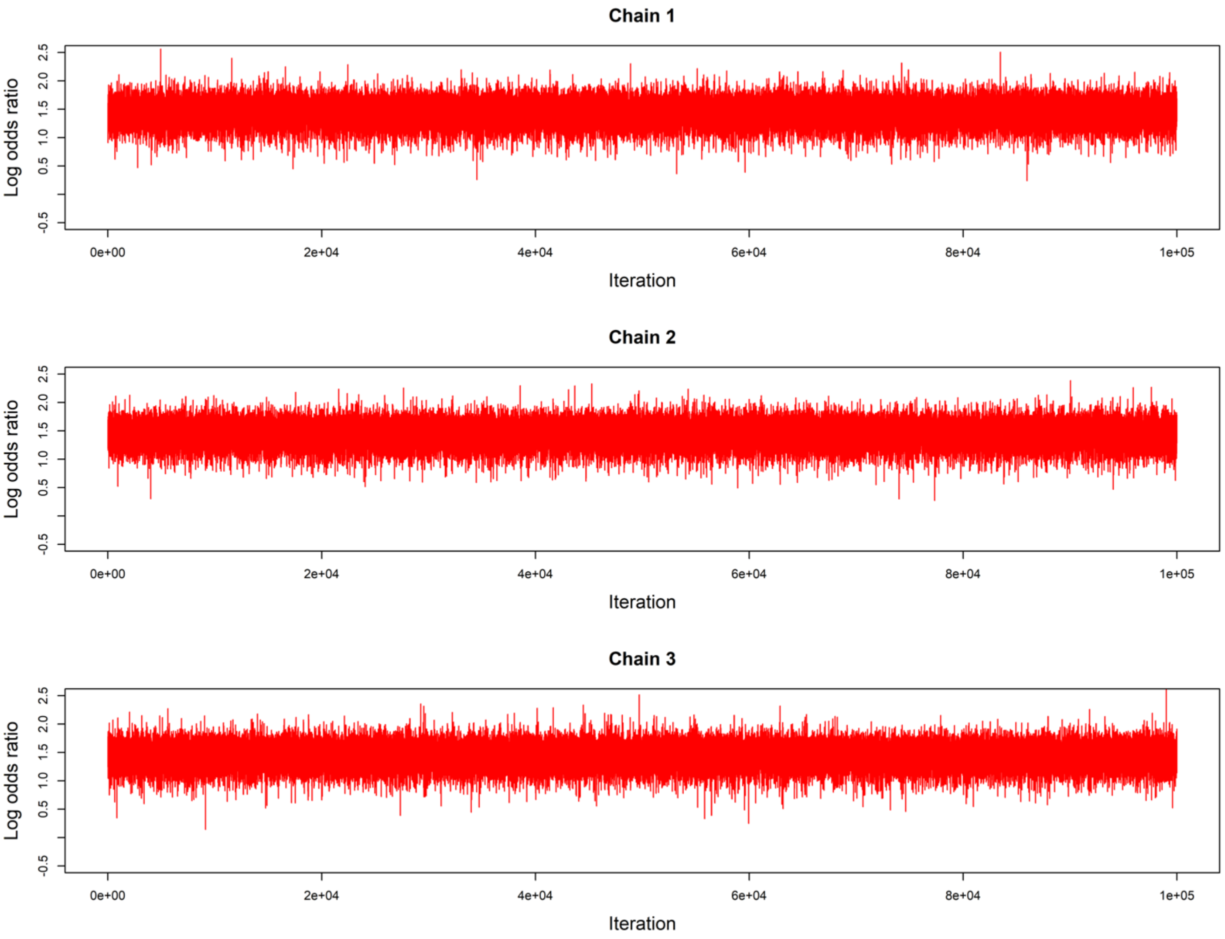

Figure S7. Trace plots of the overall log odds ratio based on the prior $IG(0.1, 0.1)$ for τ^2 in the meta-analysis on stillbirth (Example 1).

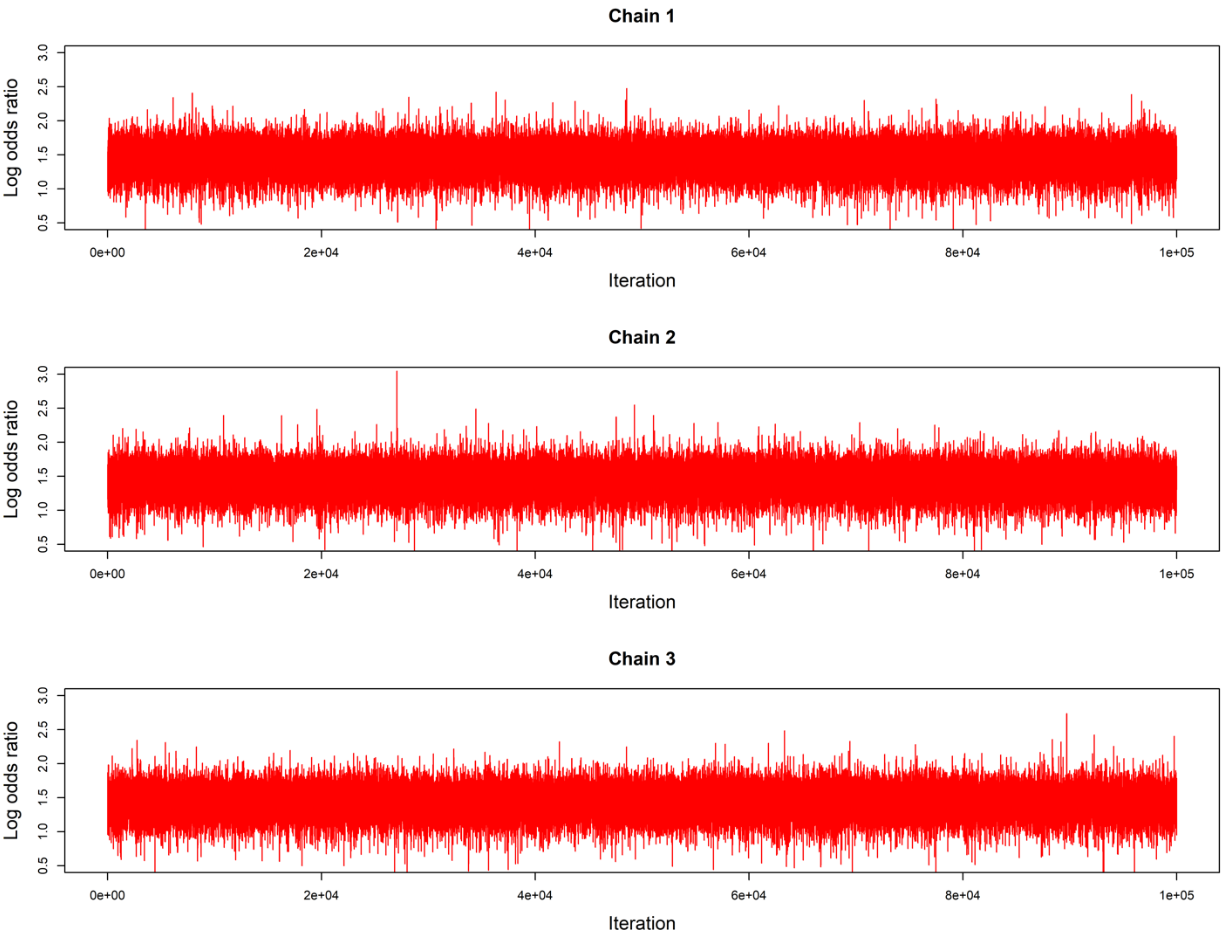

Figure S8. Trace plots of the overall log odds ratio based on the prior $U(0, 2)$ for τ in the meta-analysis on stillbirth (Example 1).

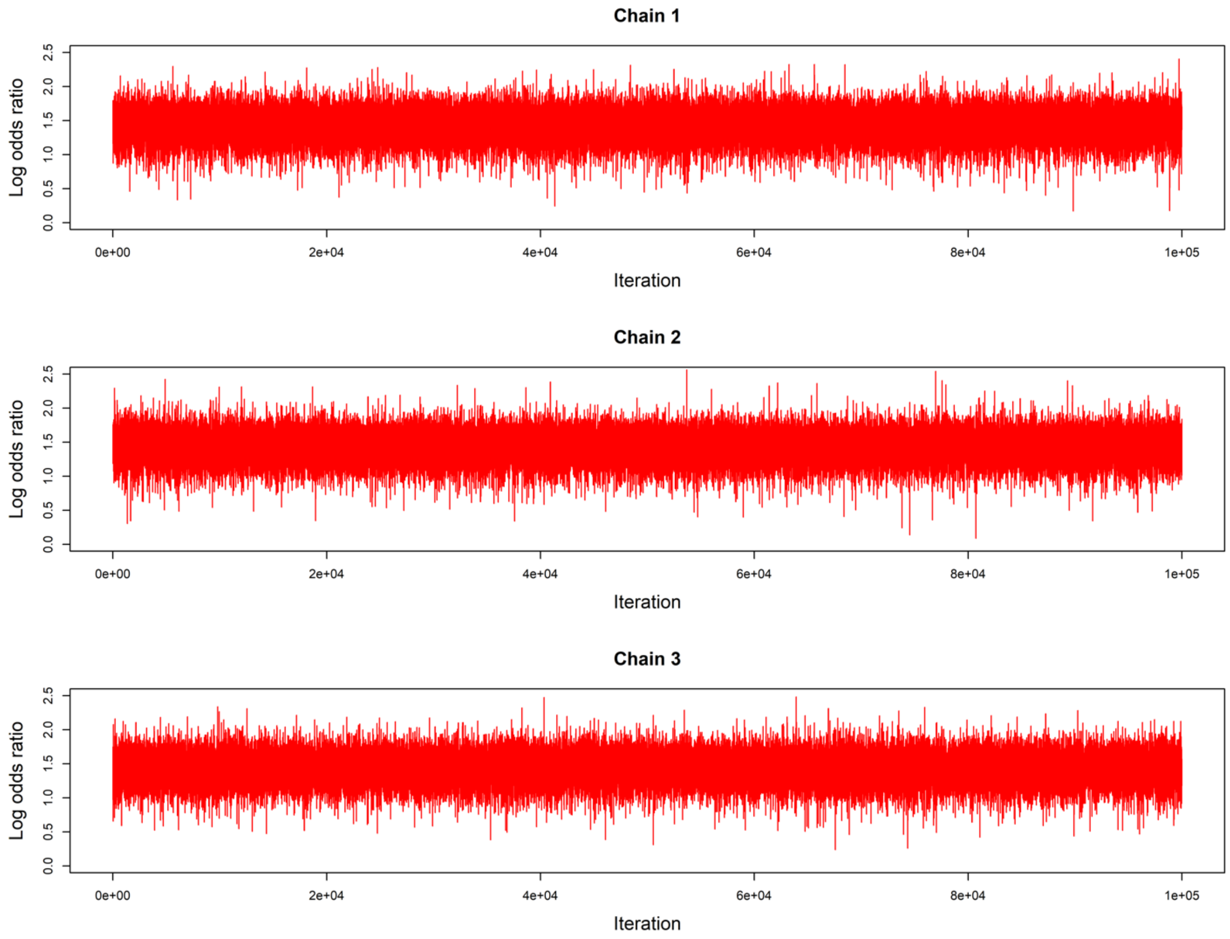

Figure S9. Trace plots of the overall log odds ratio based on the prior $U(0, 10)$ for τ in the meta-analysis on stillbirth (Example 1).

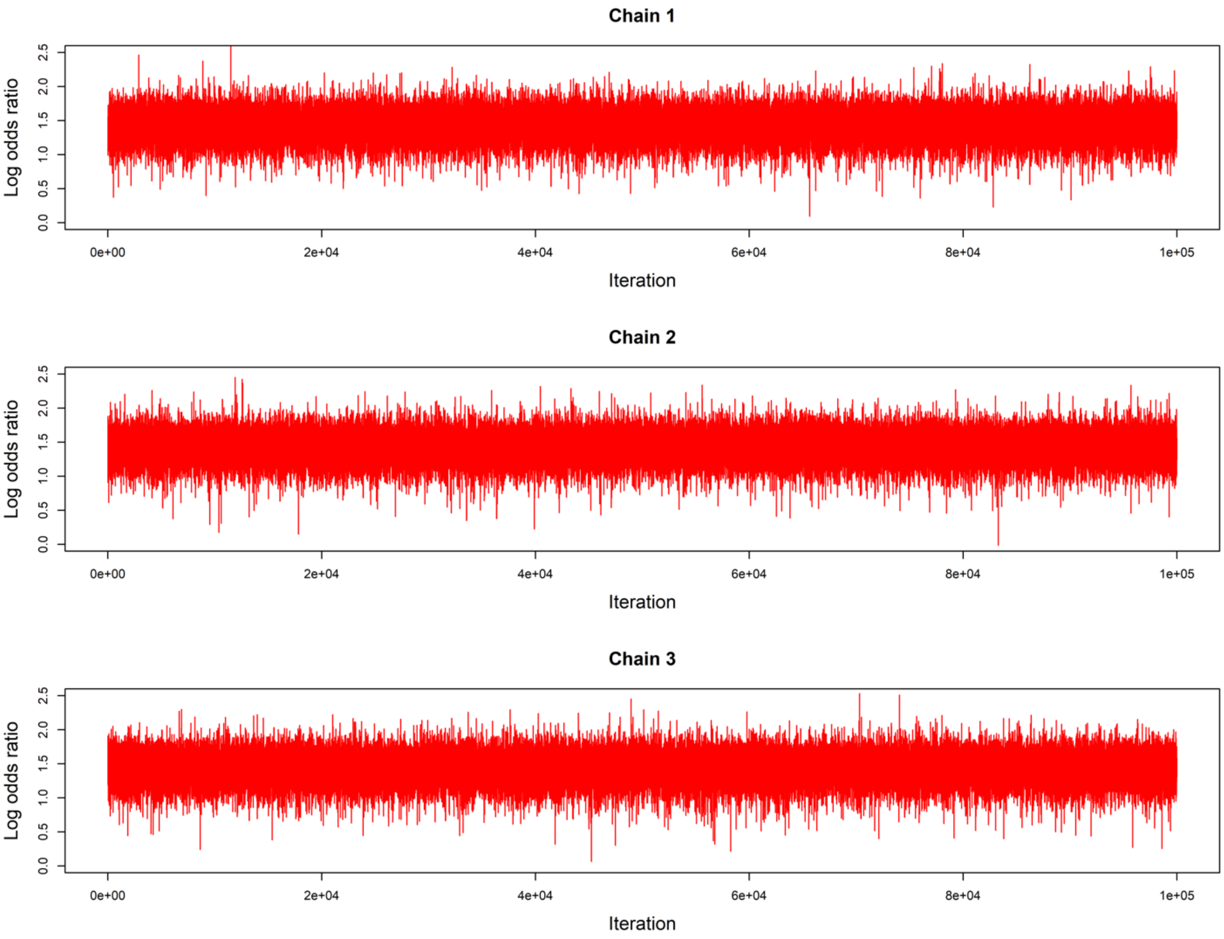

Figure S10. Trace plots of the overall log odds ratio based on the prior $U(0, 100)$ for τ in the meta-analysis on stillbirth (Example 1).

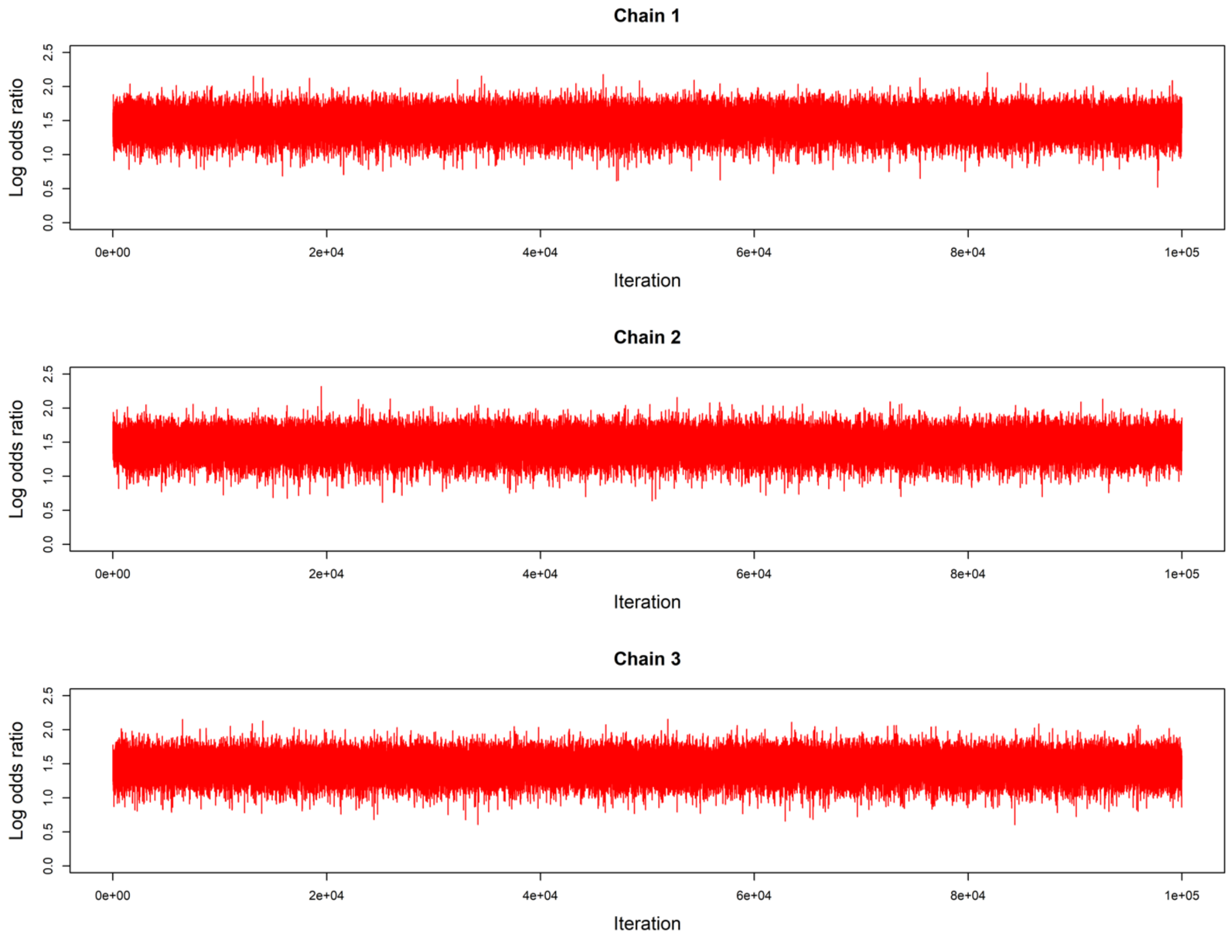

Figure S11. Trace plots of the overall log odds ratio based on the prior $HN(0, 0.1)$ for τ in the meta-analysis on stillbirth (Example 1).

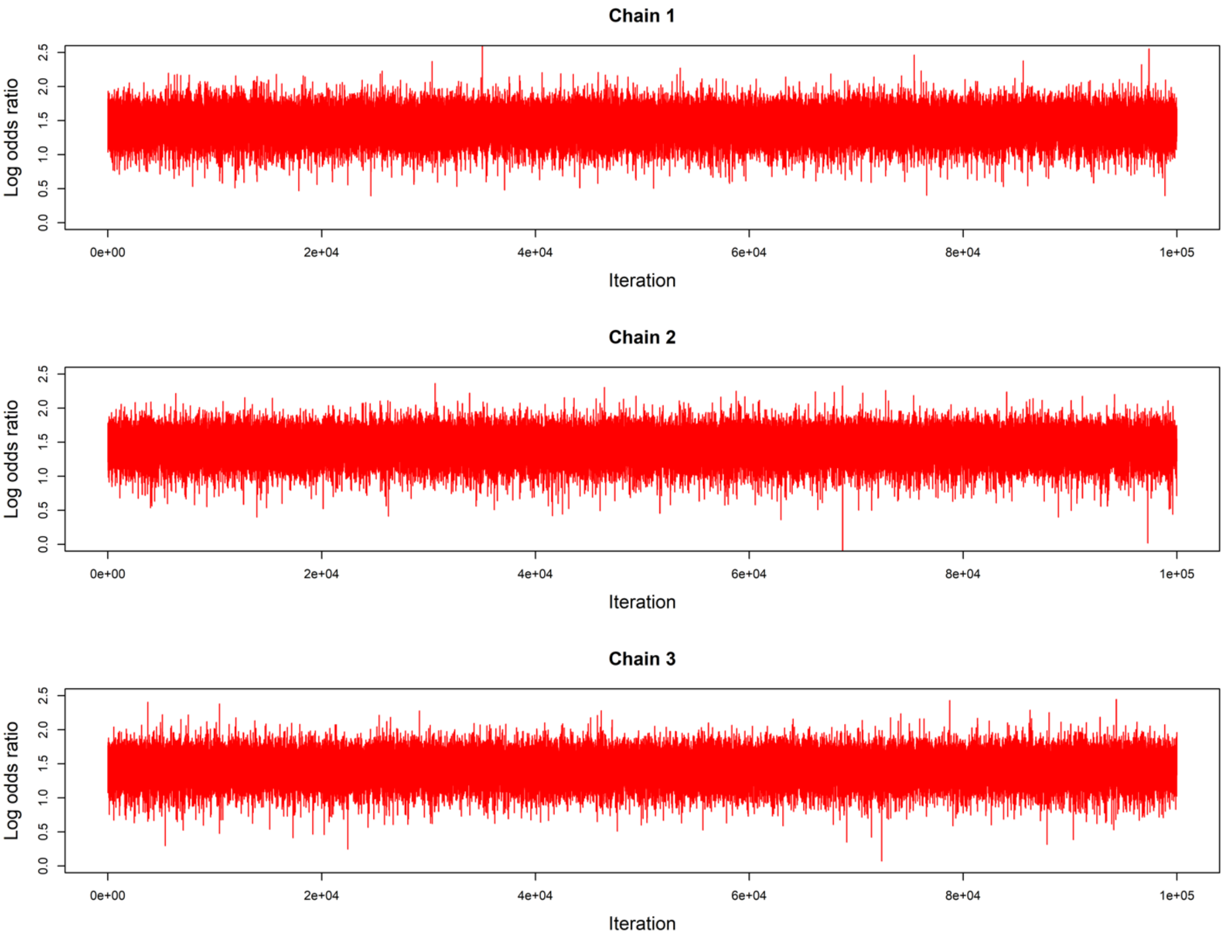

Figure S12. Trace plots of the overall log odds ratio based on the prior $HN(0, 1)$ for τ in the meta-analysis on stillbirth (Example 1).

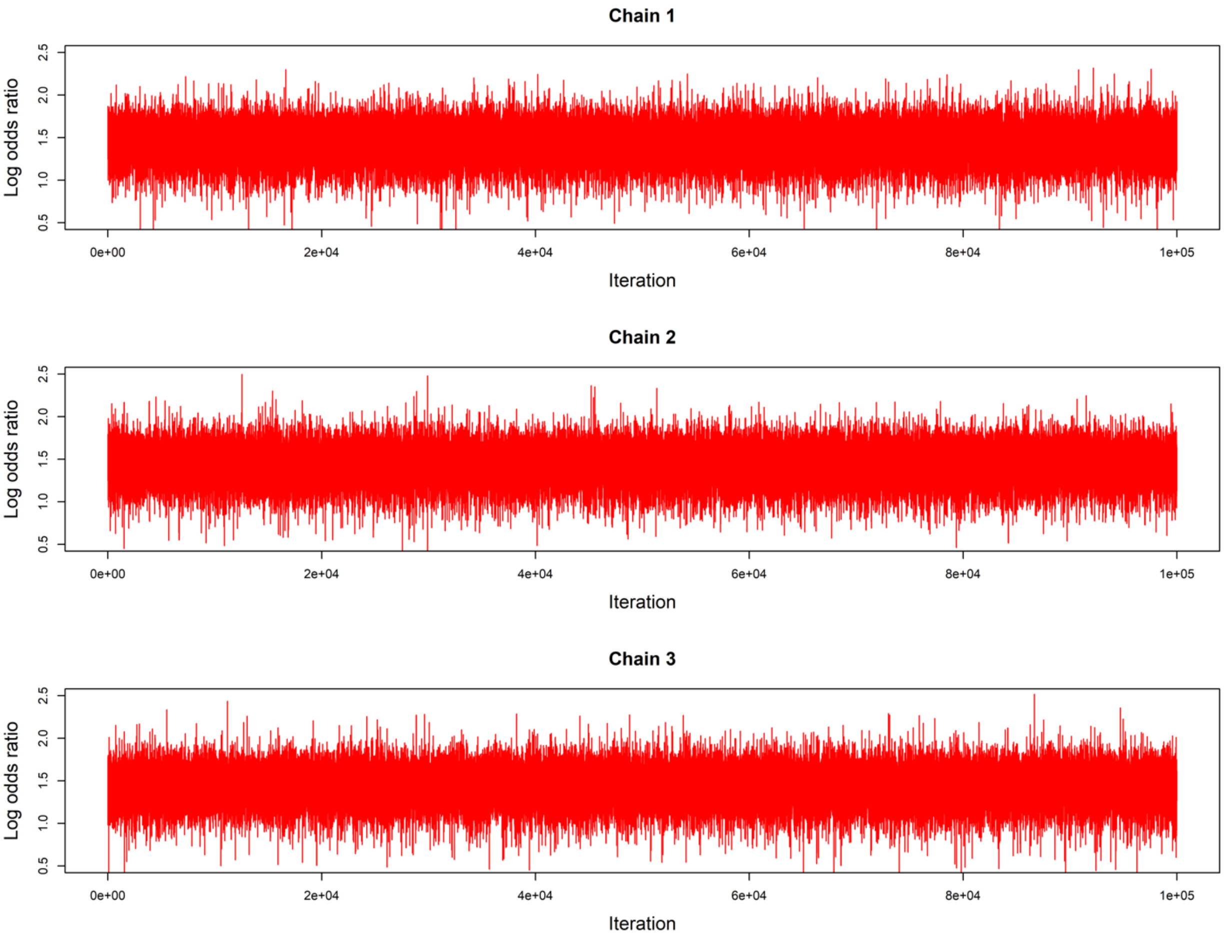

Figure S13. Trace plots of the overall log odds ratio based on the prior $HN(0, 2)$ for τ in the meta-analysis on stillbirth (Example 1).

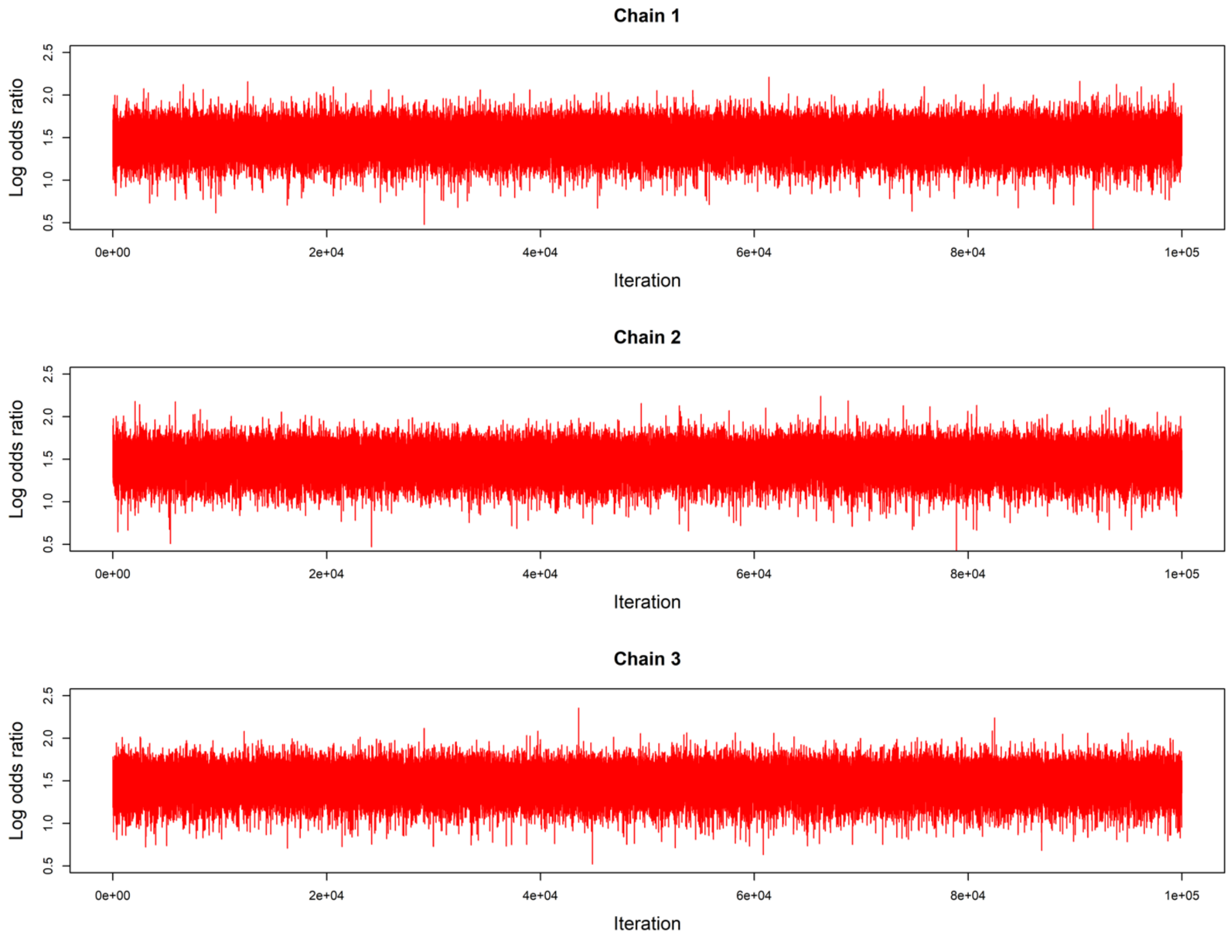

Figure S14. Trace plots of the overall log odds ratio based on the prior $LN(-3.93, 1.51^2)$ for τ^2 in the meta-analysis on stillbirth (Example 1).

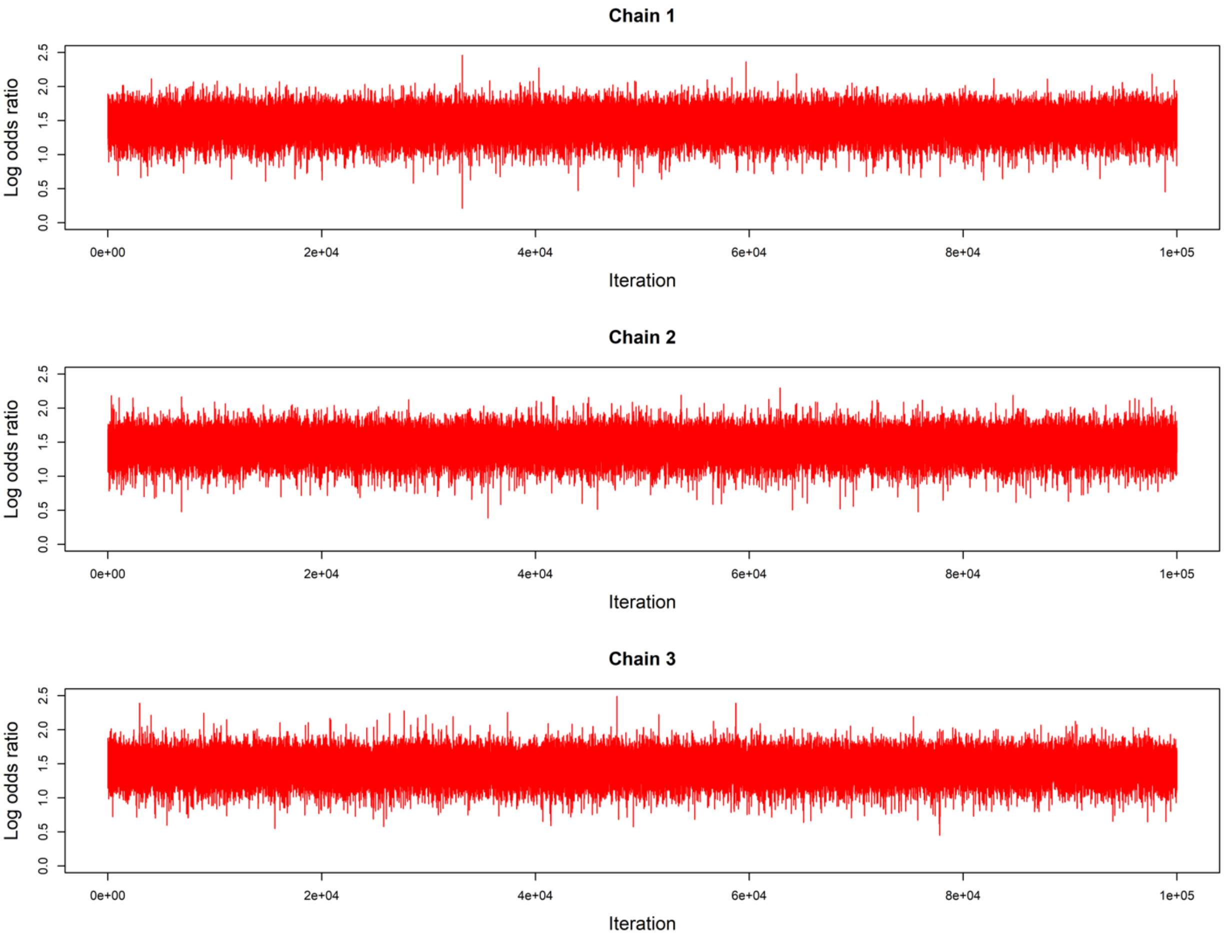

Figure S15. Trace plots of the overall log odds ratio based on the prior $LN(-2.89, 1.91^2)$ for τ^2 in the meta-analysis on stillbirth (Example 1).

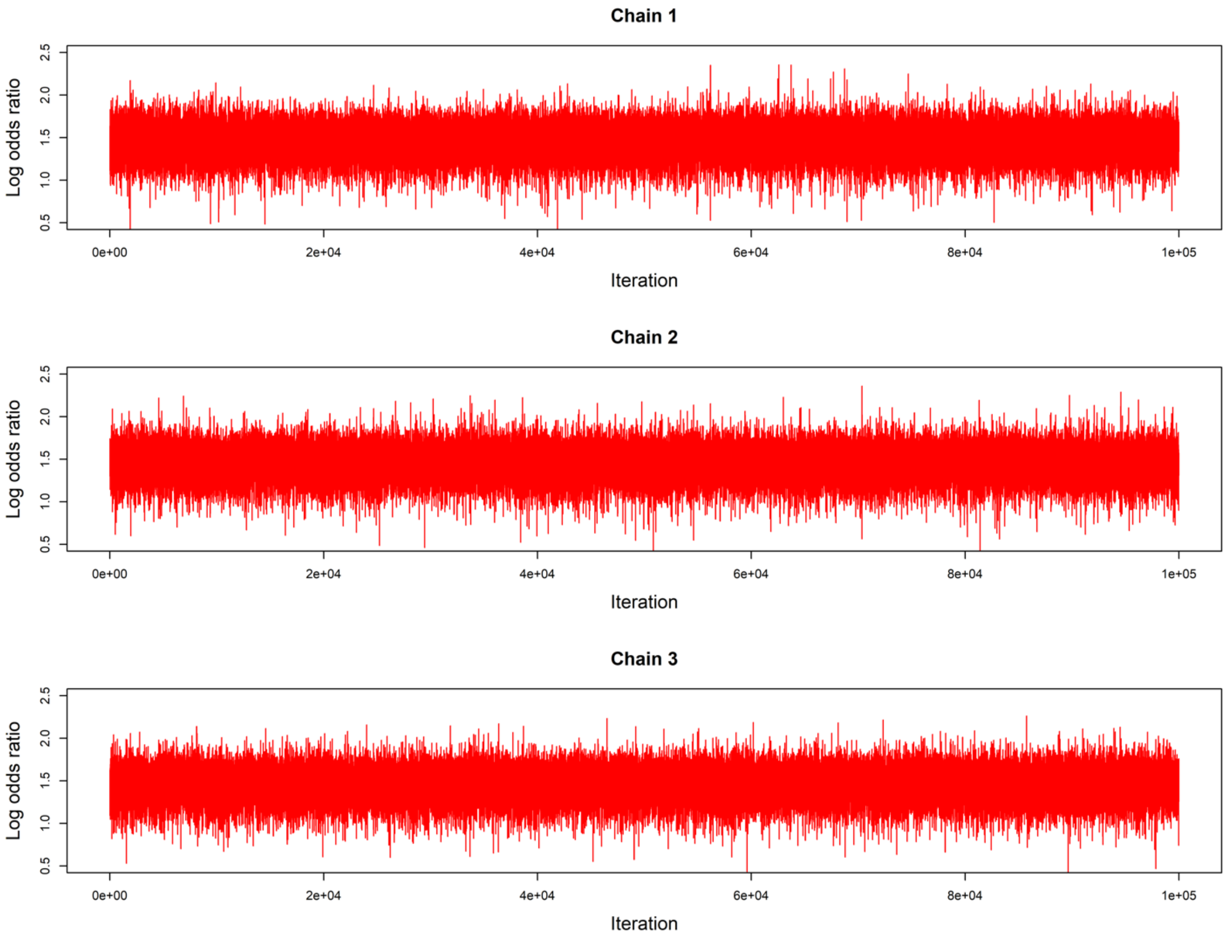

Figure S16. Trace plots of the overall log odds ratio based on the prior $LN(-2.01, 1.64^2)$ for τ^2 in the meta-analysis on stillbirth (Example 1).

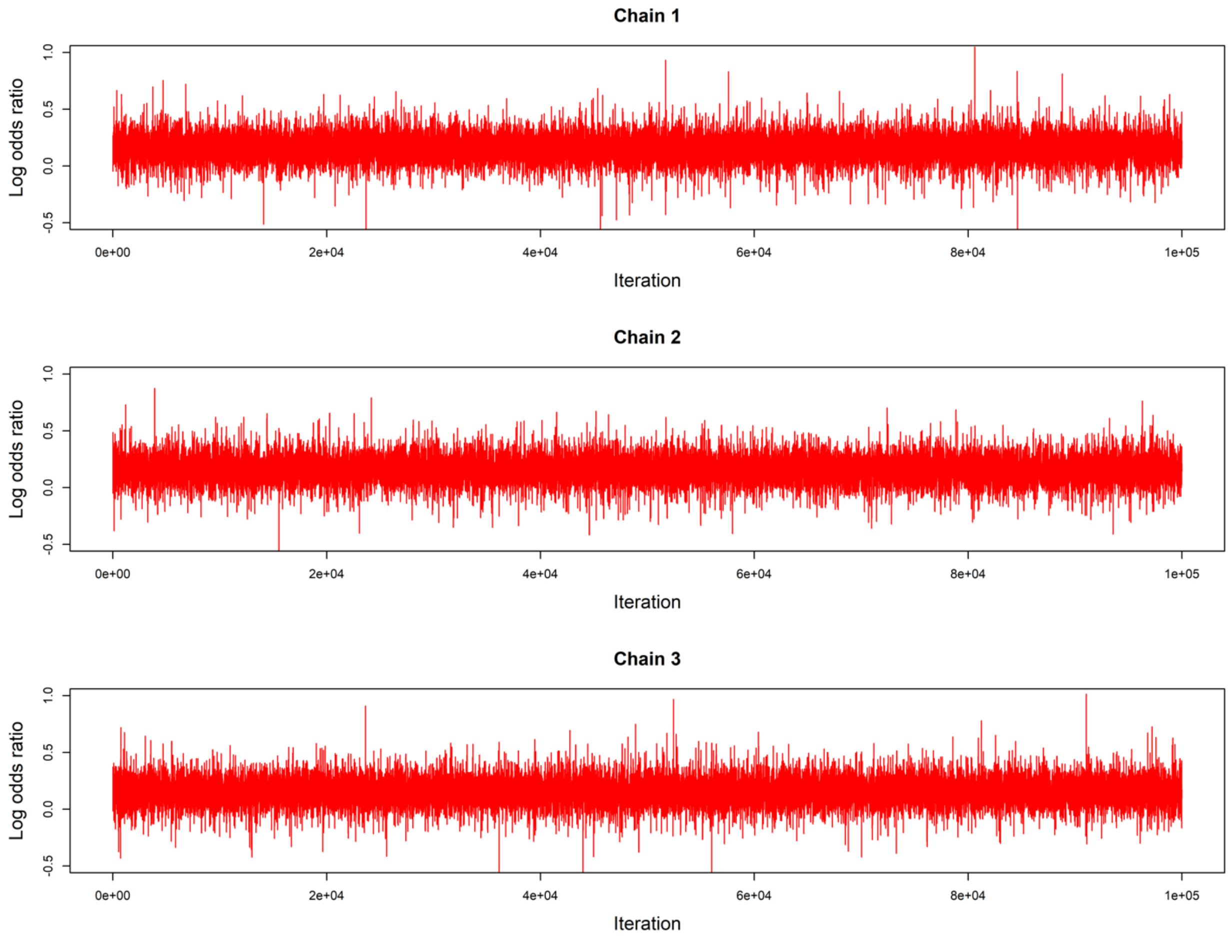

Figure S17. Trace plots of the overall log odds ratio based on the prior $IG(0.001, 0.001)$ for τ^2 in the meta-analysis on patient enrollment in clinical trials (Example 2).

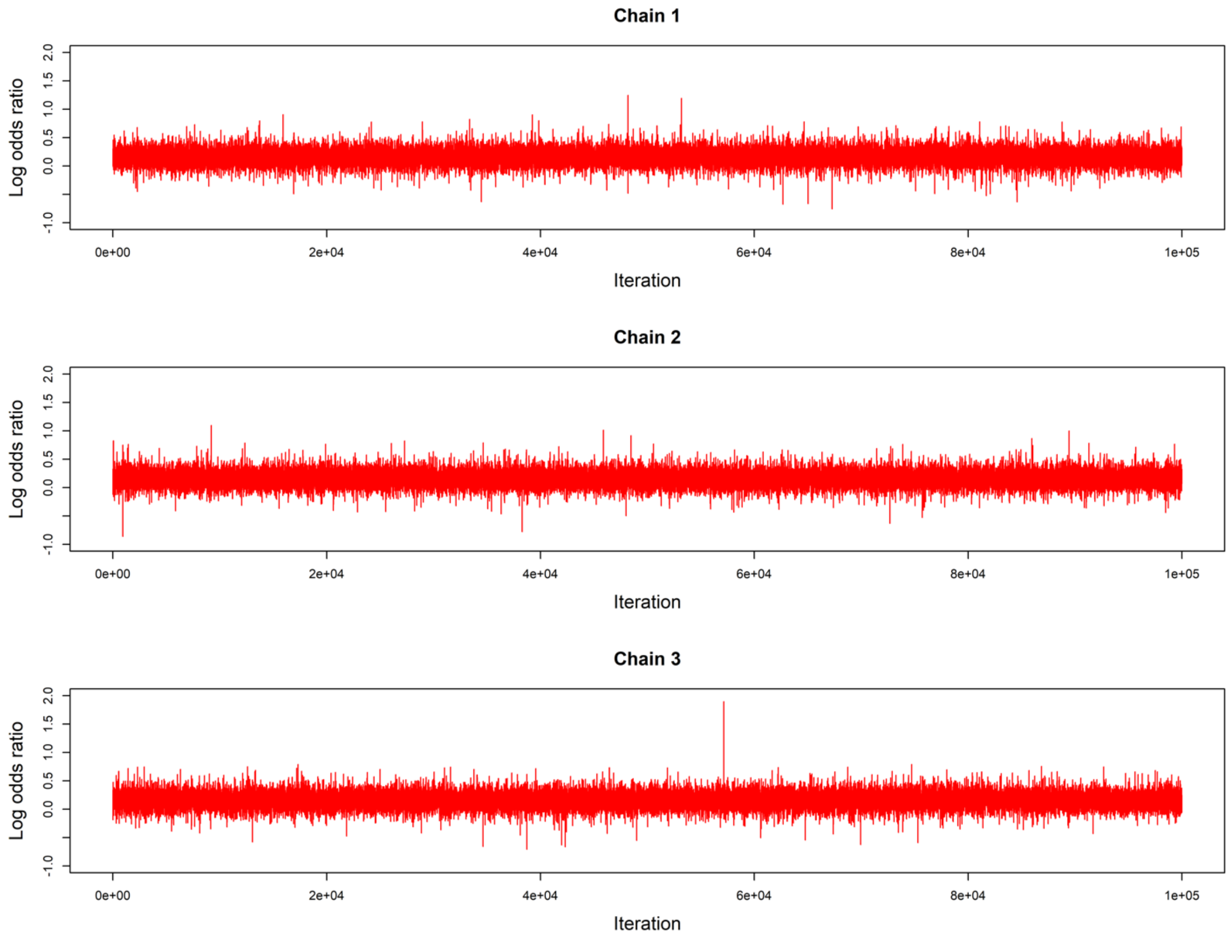

Figure S18. Trace plots of the overall log odds ratio based on the prior $IG(0.01, 0.01)$ for τ^2 in the meta-analysis on patient enrollment in clinical trials (Example 2).

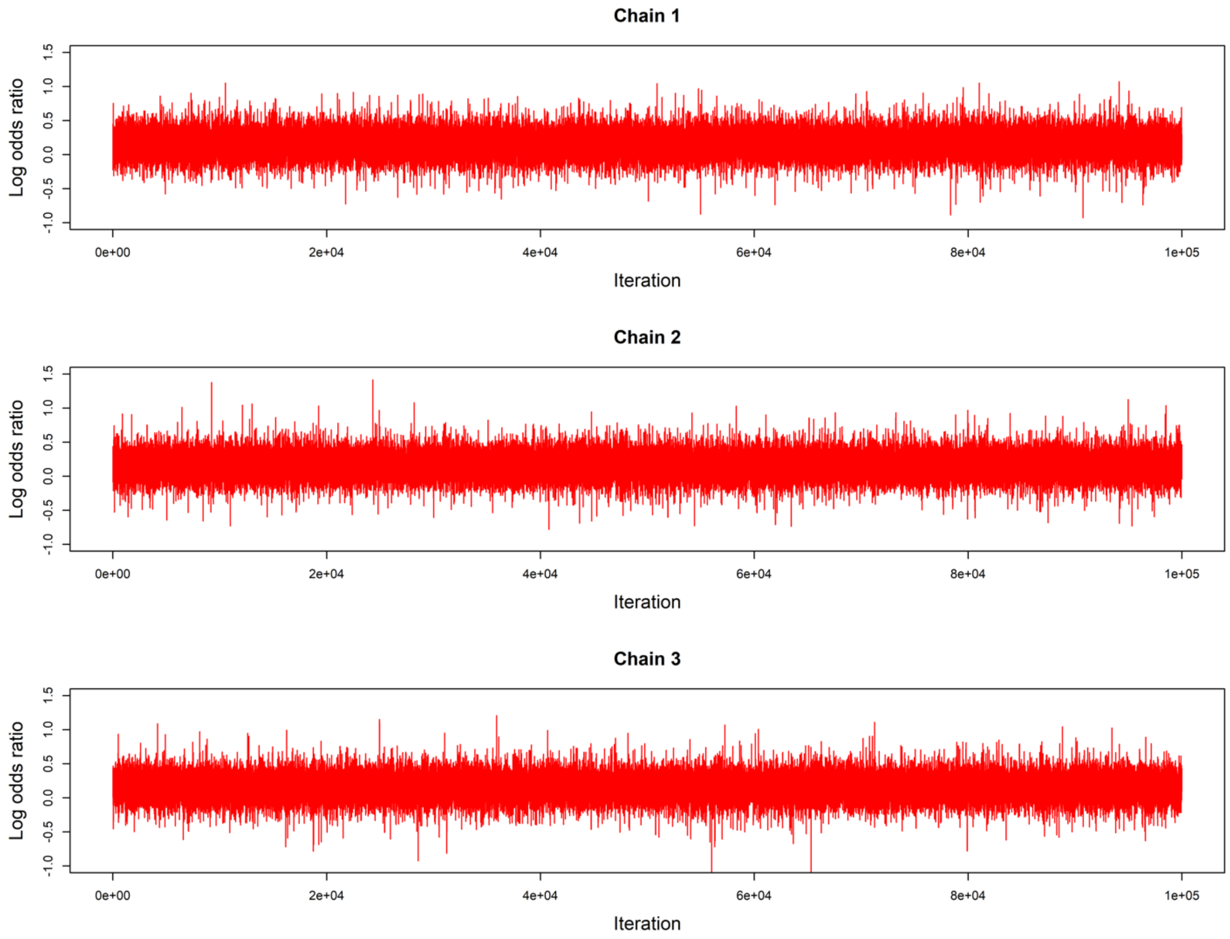

Figure S19. Trace plots of the overall log odds ratio based on the prior $IG(0.1, 0.1)$ for τ^2 in the meta-analysis on patient enrollment in clinical trials (Example 2).

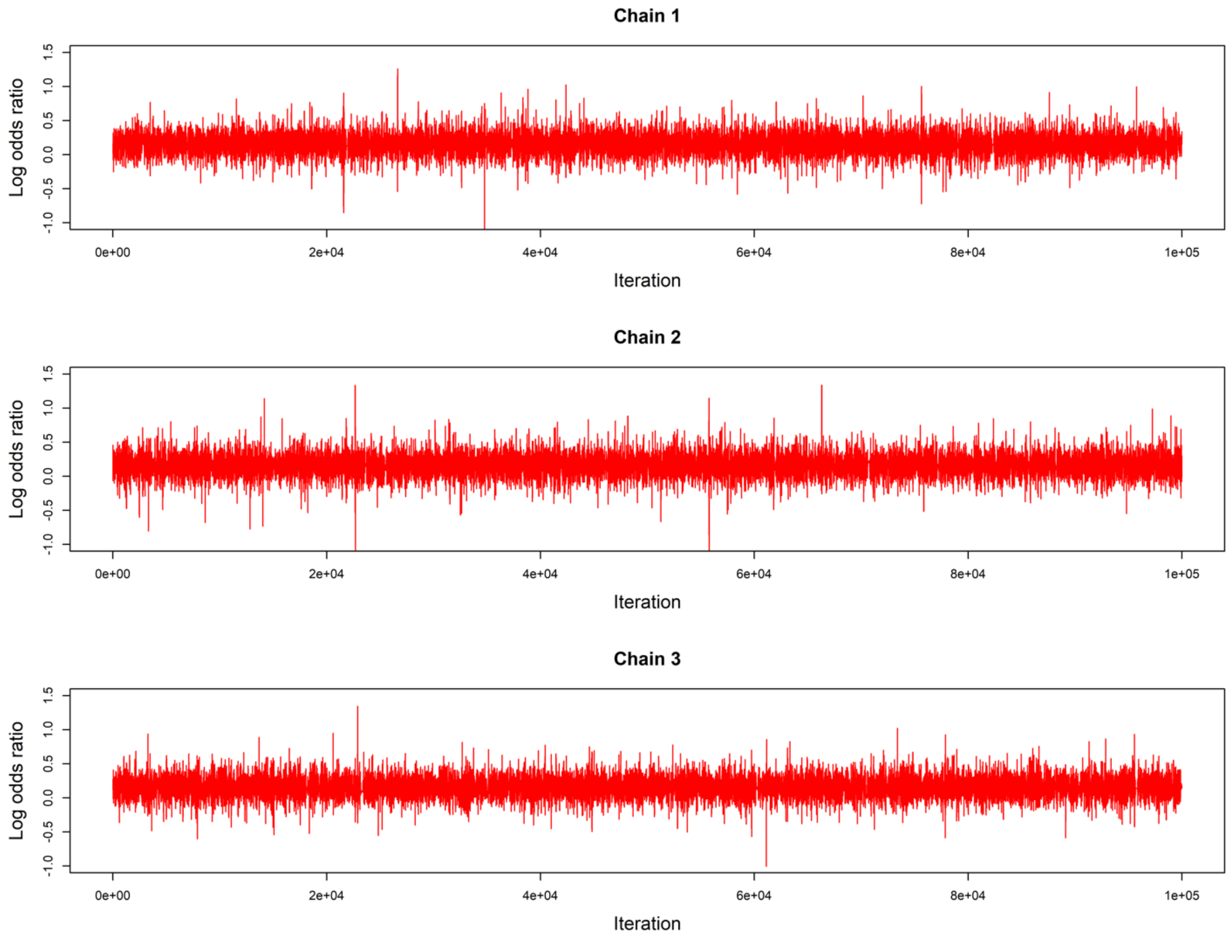

Figure S20. Trace plots of the overall log odds ratio based on the prior $U(0, 2)$ for τ in the meta-analysis on patient enrollment in clinical trials (Example 2).

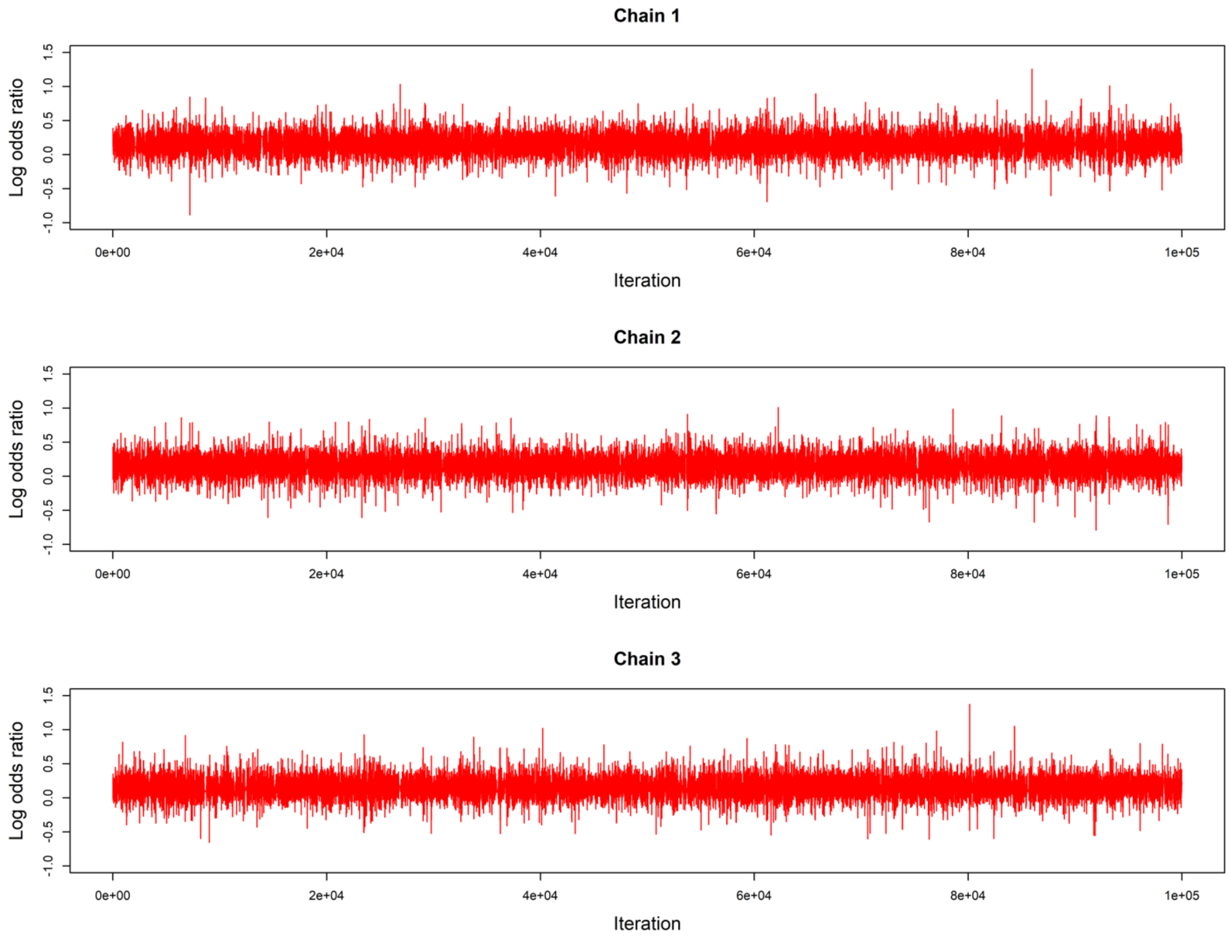

Figure S21. Trace plots of the overall log odds ratio based on the prior $U(0, 10)$ for τ in the meta-analysis on patient enrollment in clinical trials (Example 2).

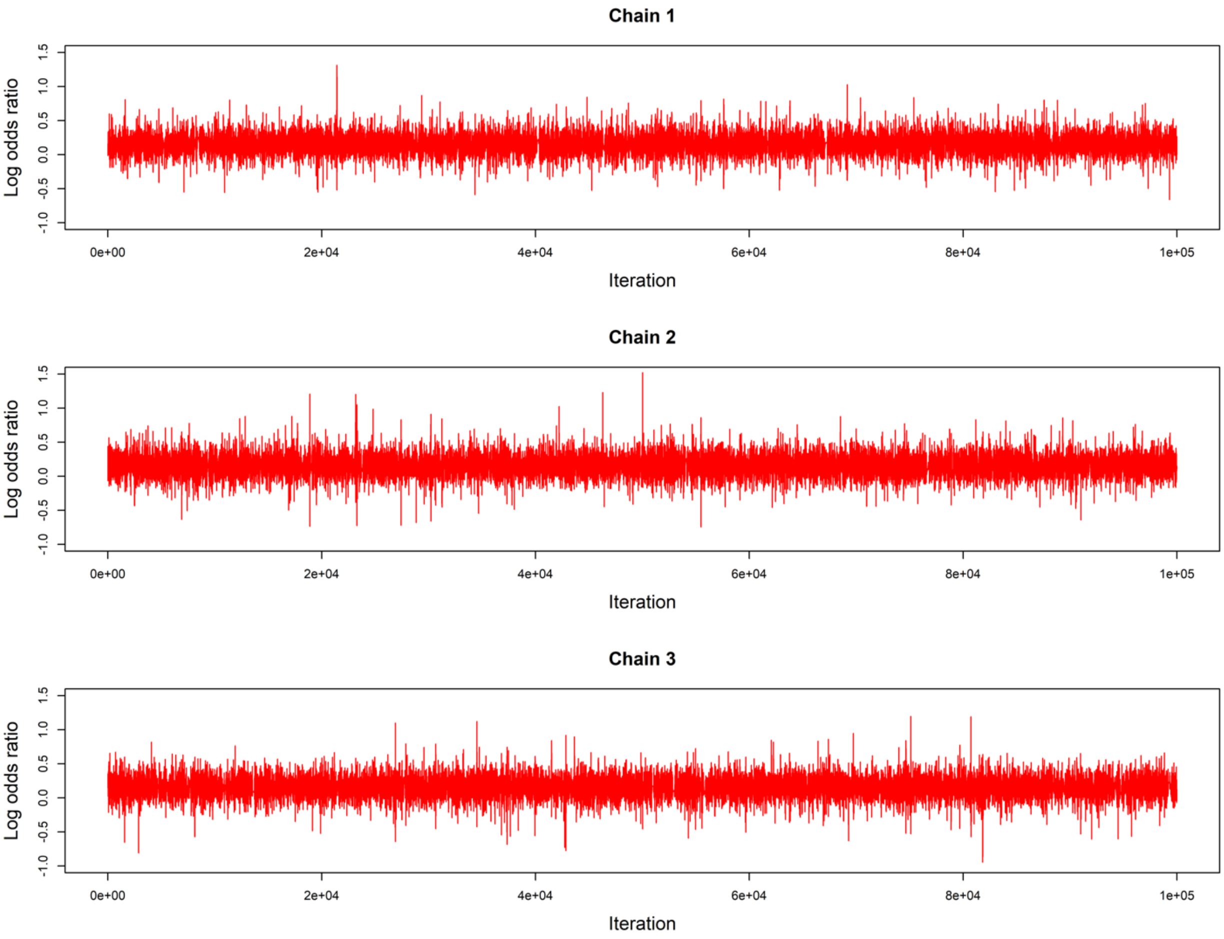

Figure S22. Trace plots of the overall log odds ratio based on the prior $U(0, 100)$ for τ in the meta-analysis on patient enrollment in clinical trials (Example 2).

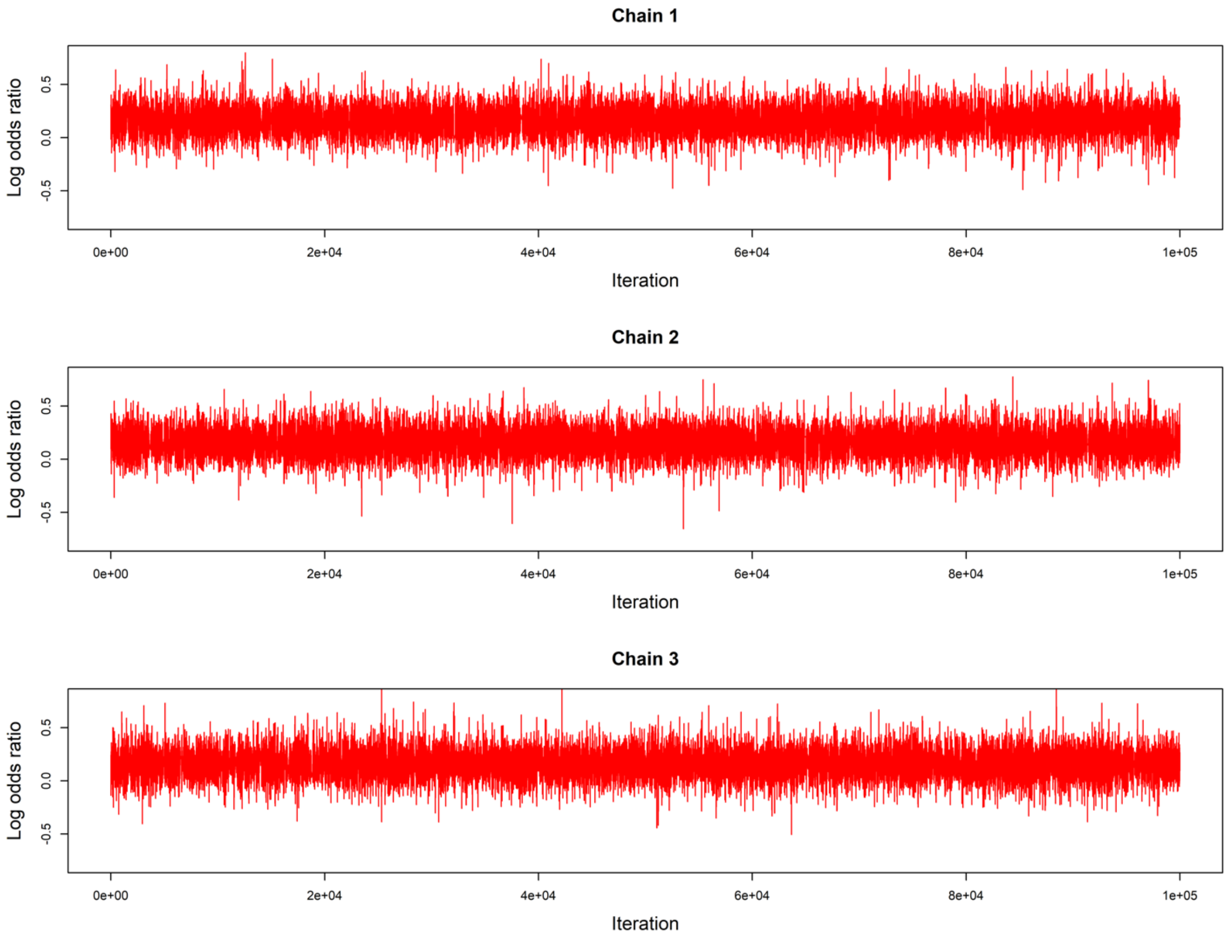

Figure S23. Trace plots of the overall log odds ratio based on the prior $HN(0, 0.1)$ for τ in the meta-analysis on patient enrollment in clinical trials (Example 2).

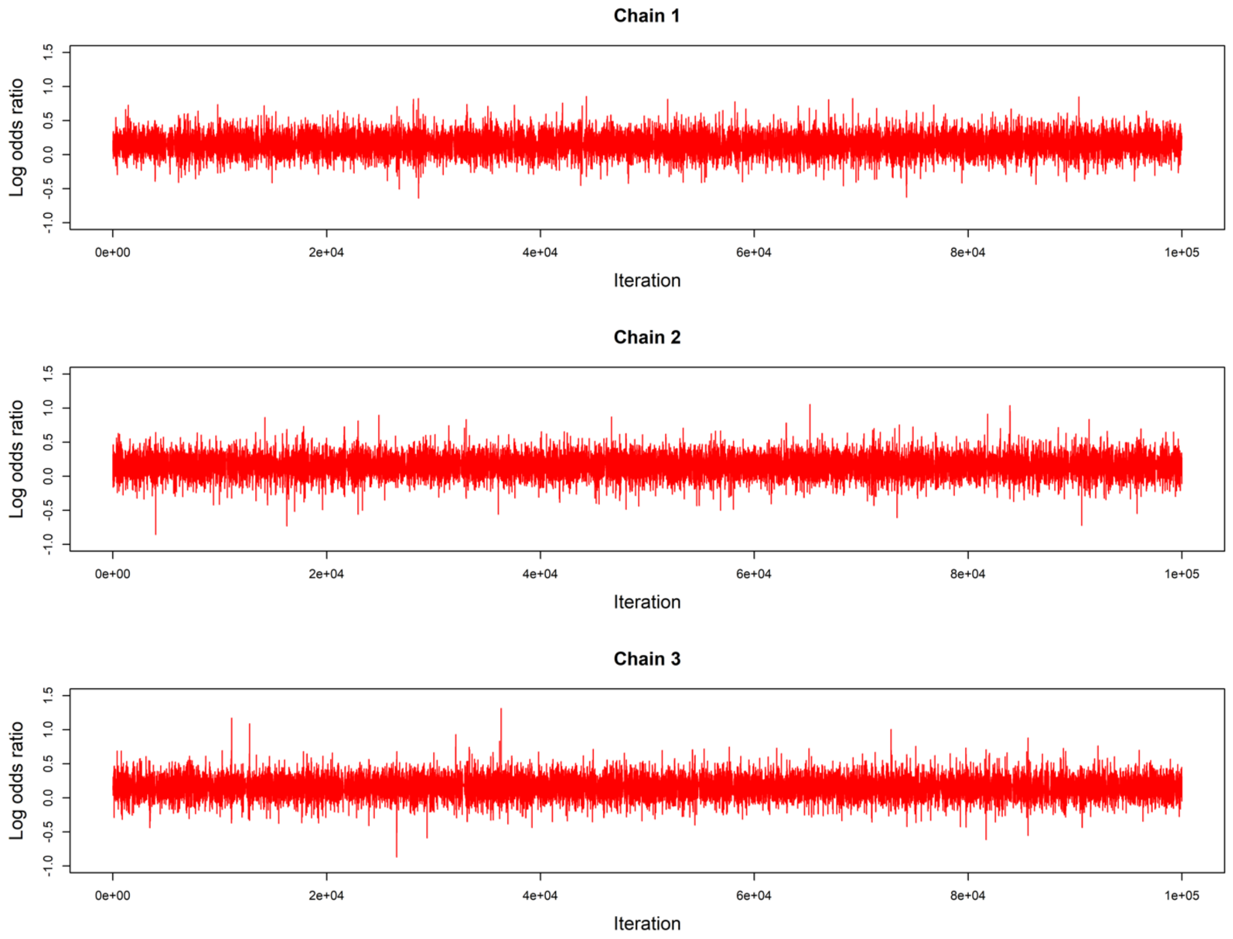

Figure S24. Trace plots of the overall log odds ratio based on the prior $HN(0, 1)$ for τ in the meta-analysis on patient enrollment in clinical trials (Example 2).

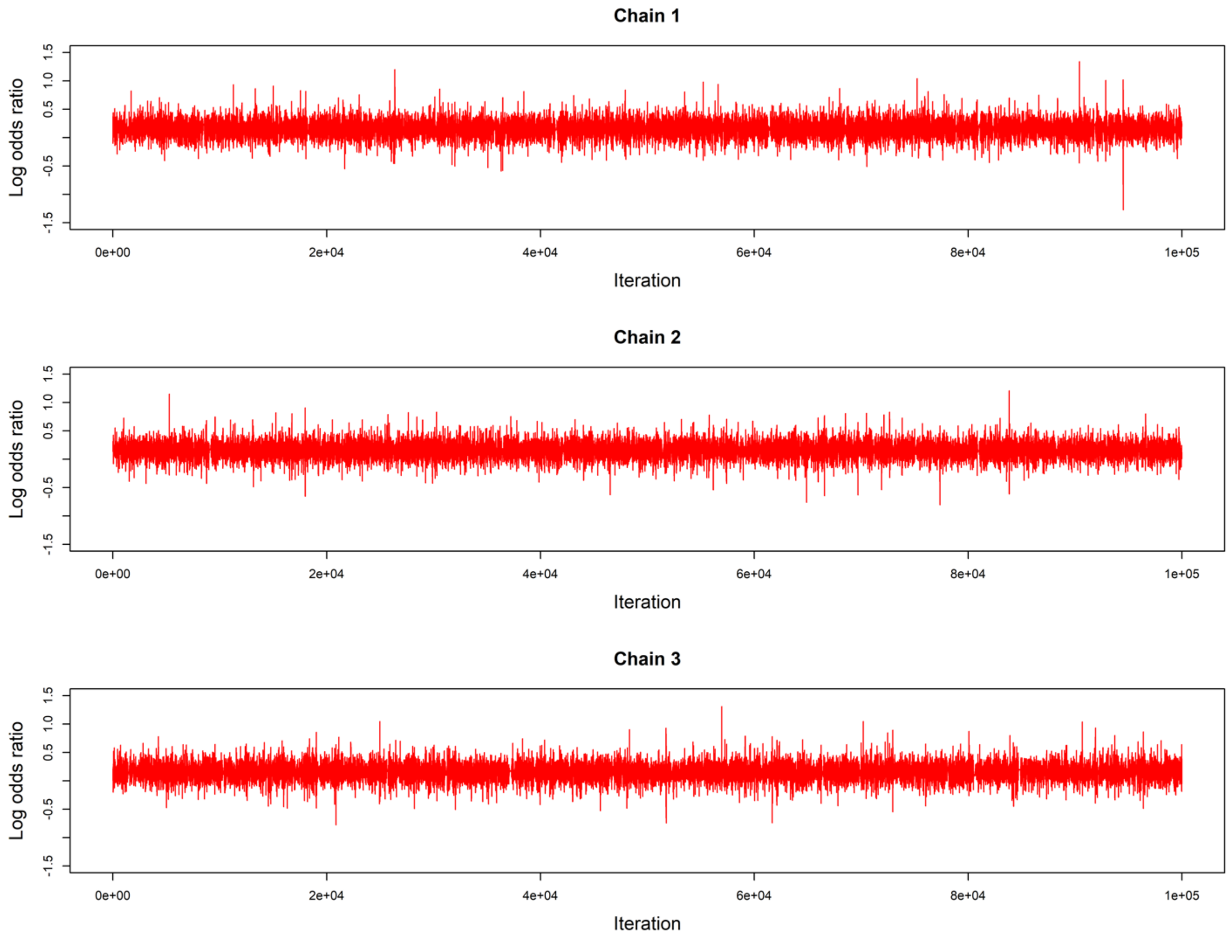

Figure S25. Trace plots of the overall log odds ratio based on the prior $HN(0, 2)$ for τ in the meta-analysis on patient enrollment in clinical trials (Example 2).

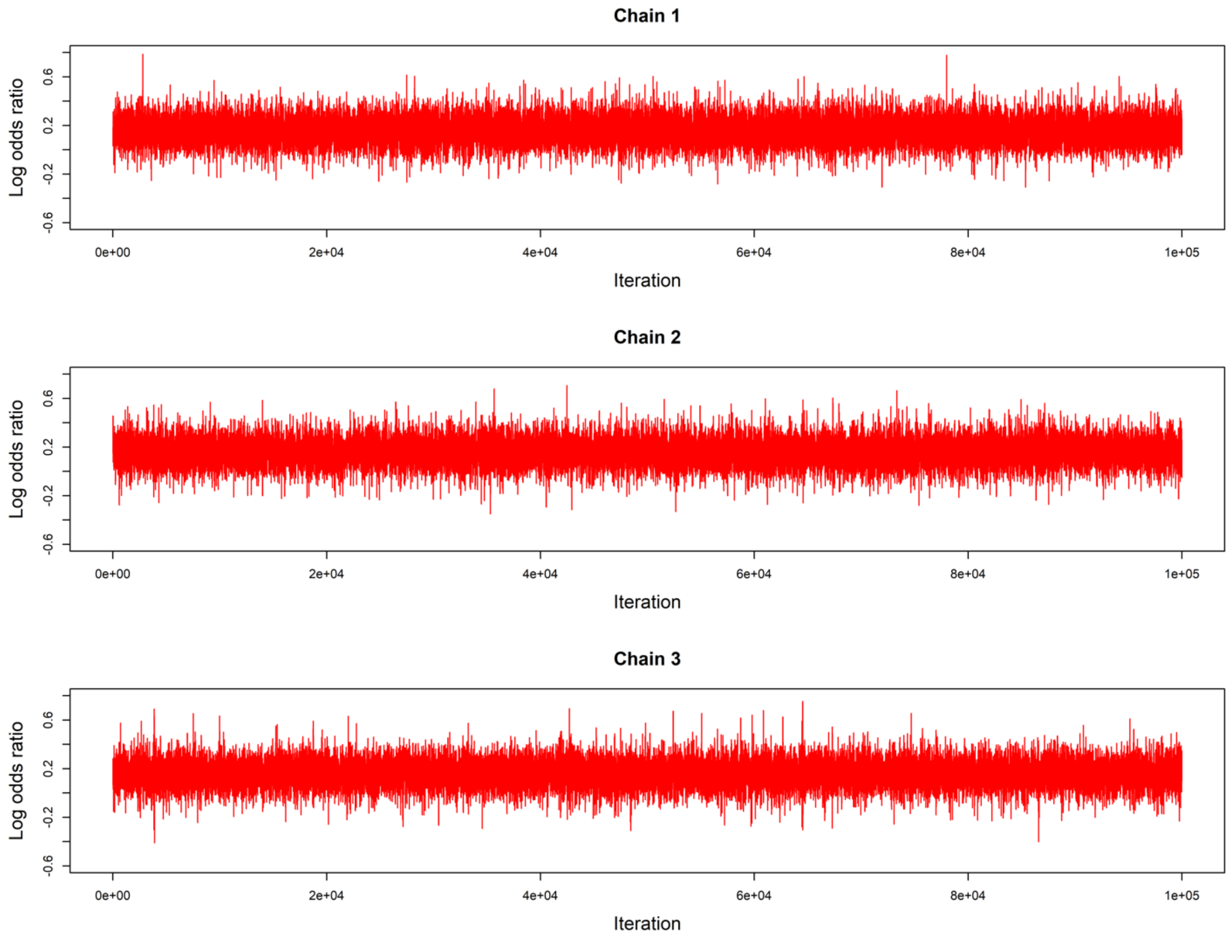

Figure S26. Trace plots of the overall log odds ratio based on the prior $LN(-3.93, 1.51^2)$ for τ^2 in the meta-analysis on patient enrollment in clinical trials (Example 2).

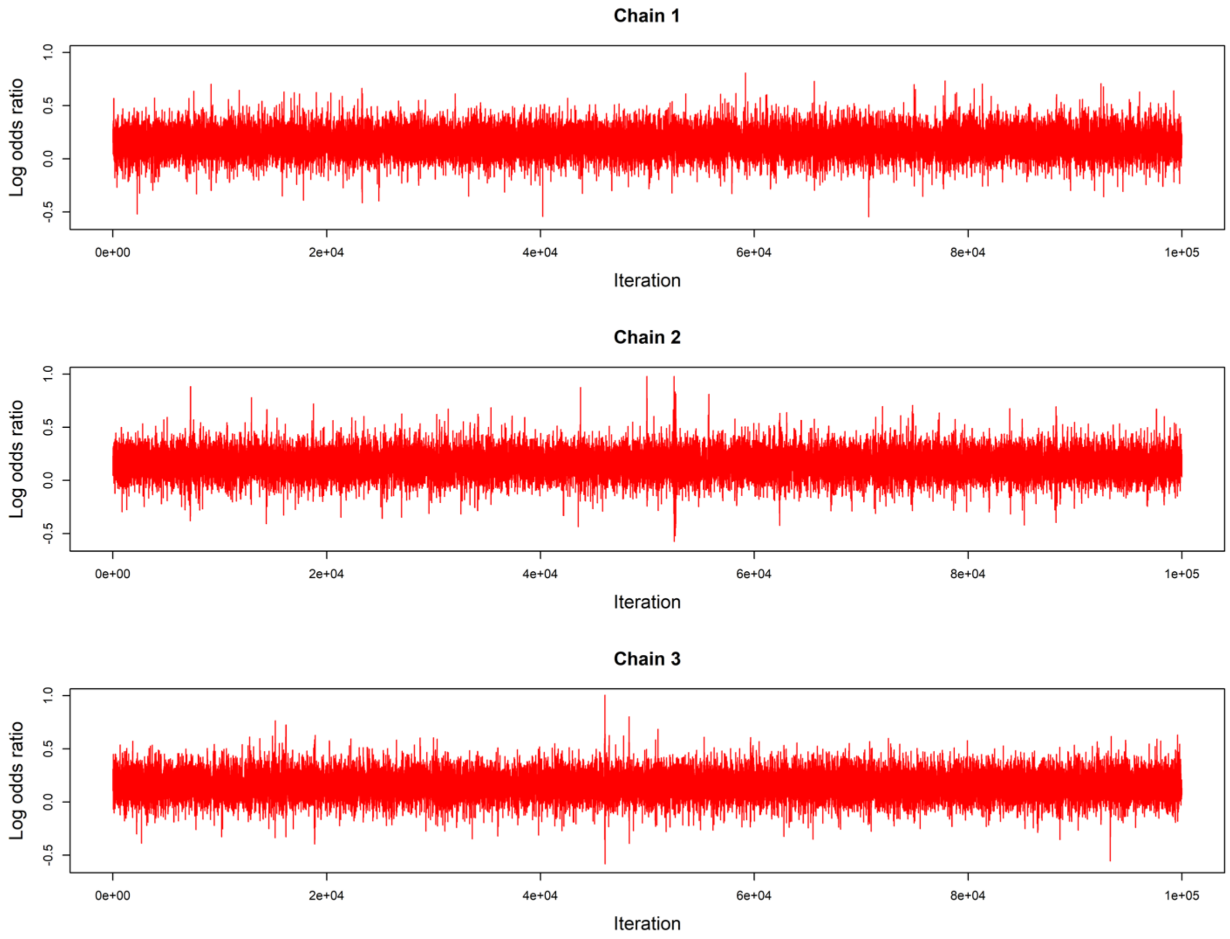

Figure S27. Trace plots of the overall log odds ratio based on the prior $LN(-2.89, 1.91^2)$ for τ^2 in the meta-analysis on patient enrollment in clinical trials (Example 2).

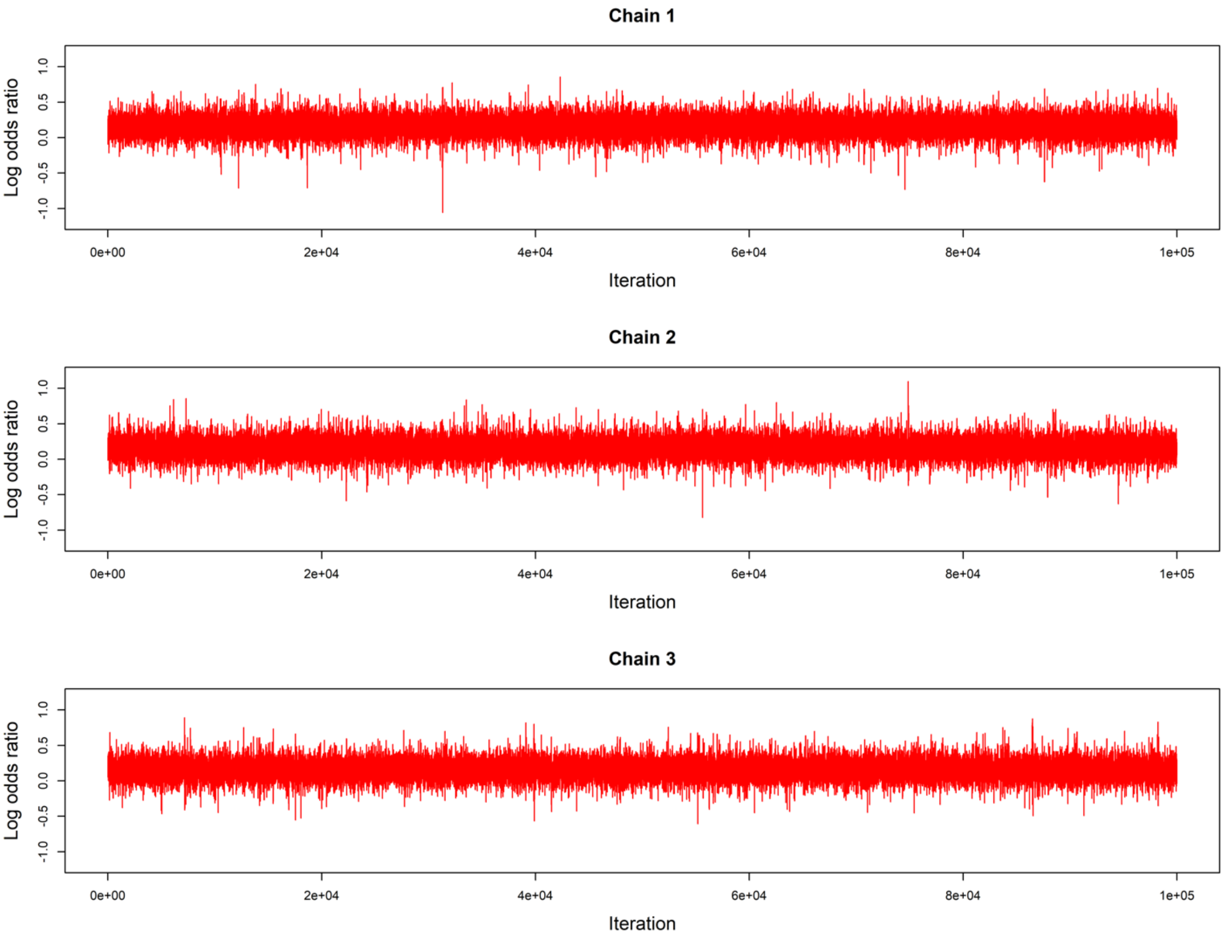

Figure S28. Trace plots of the overall log odds ratio based on the prior $LN(-2.01, 1.64^2)$ for τ^2 in the meta-analysis on patient enrollment in clinical trials (Example 2).

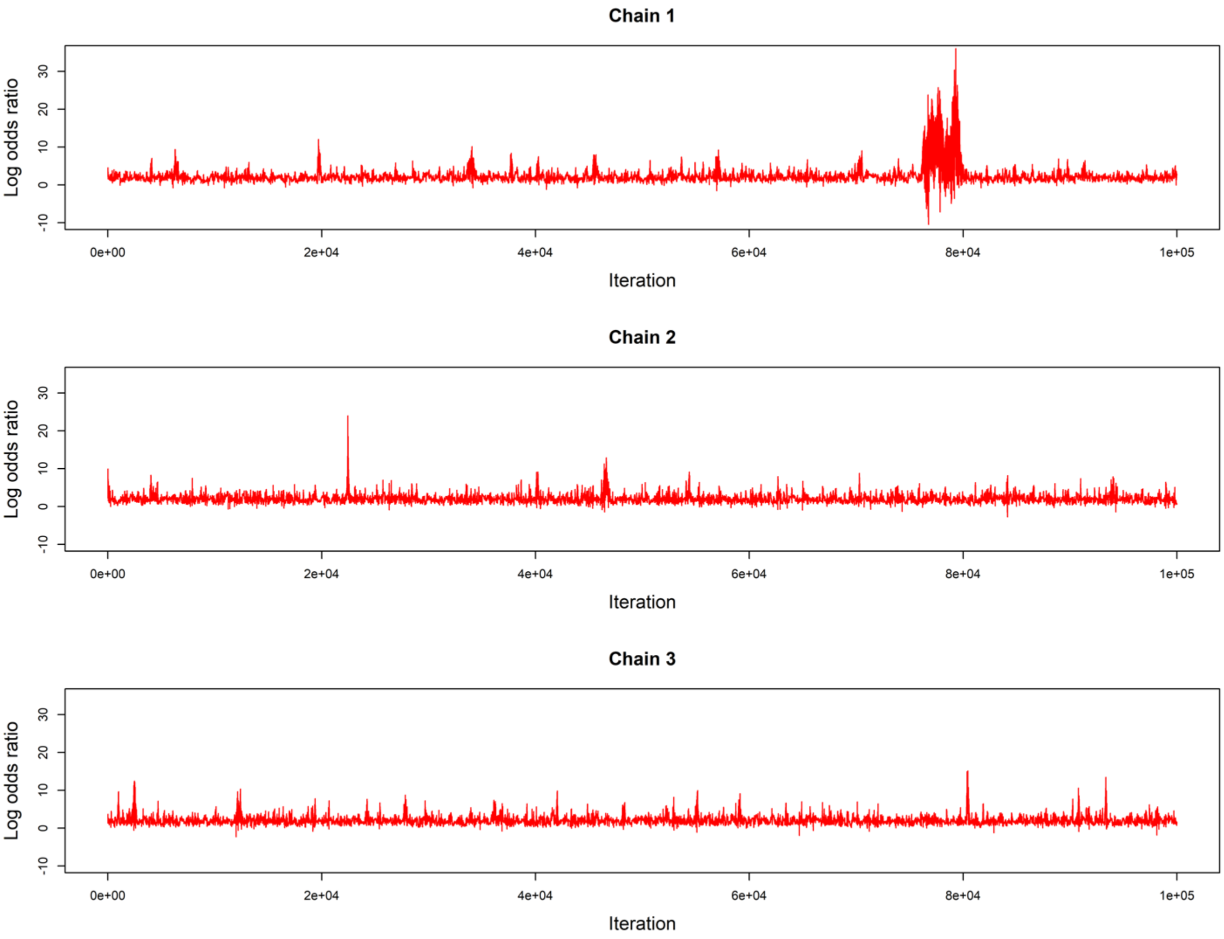

Figure S29. Trace plots of the overall log odds ratio based on the prior $IG(0.001, 0.001)$ for τ^2 in the meta-analysis on colitis (Example 3).

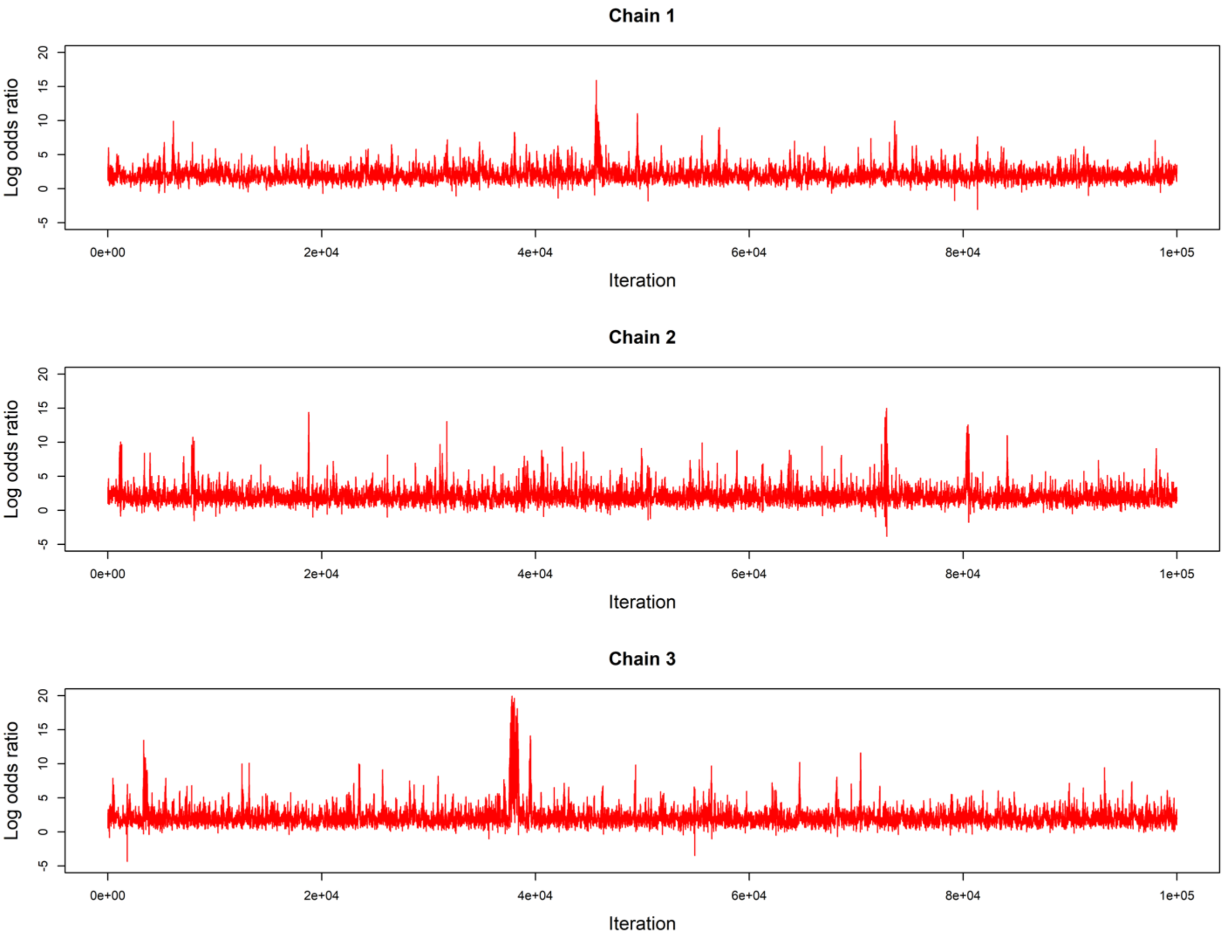

Figure S30. Trace plots of the overall log odds ratio based on the prior $IG(0.01, 0.01)$ for τ^2 in the meta-analysis on colitis (Example 3).

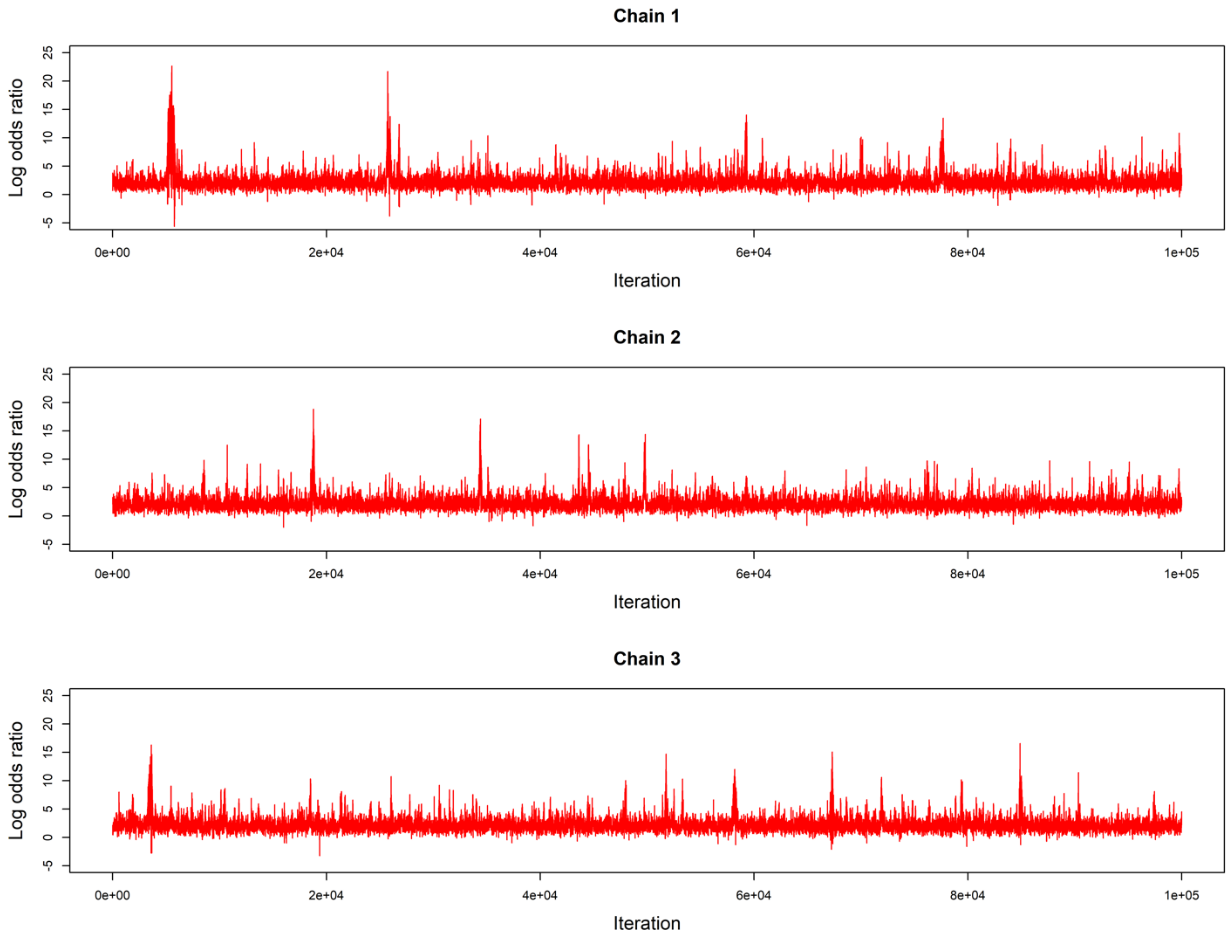

Figure S31. Trace plots of the overall log odds ratio based on the prior $IG(0.1, 0.1)$ for τ^2 in the meta-analysis on colitis (Example 3).

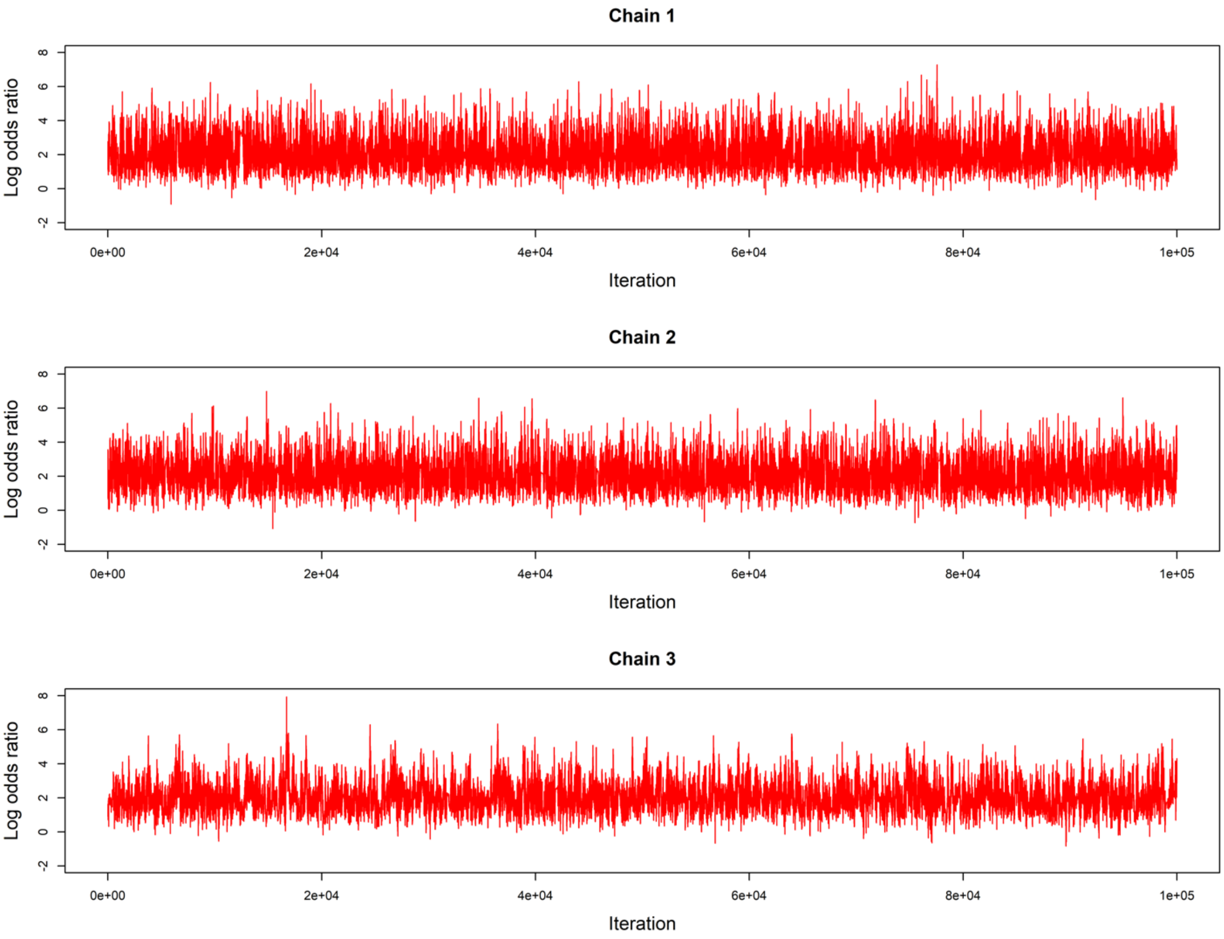

Figure S32. Trace plots of the overall log odds ratio based on the prior $U(0, 2)$ for τ in the meta-analysis on colitis (Example 3).

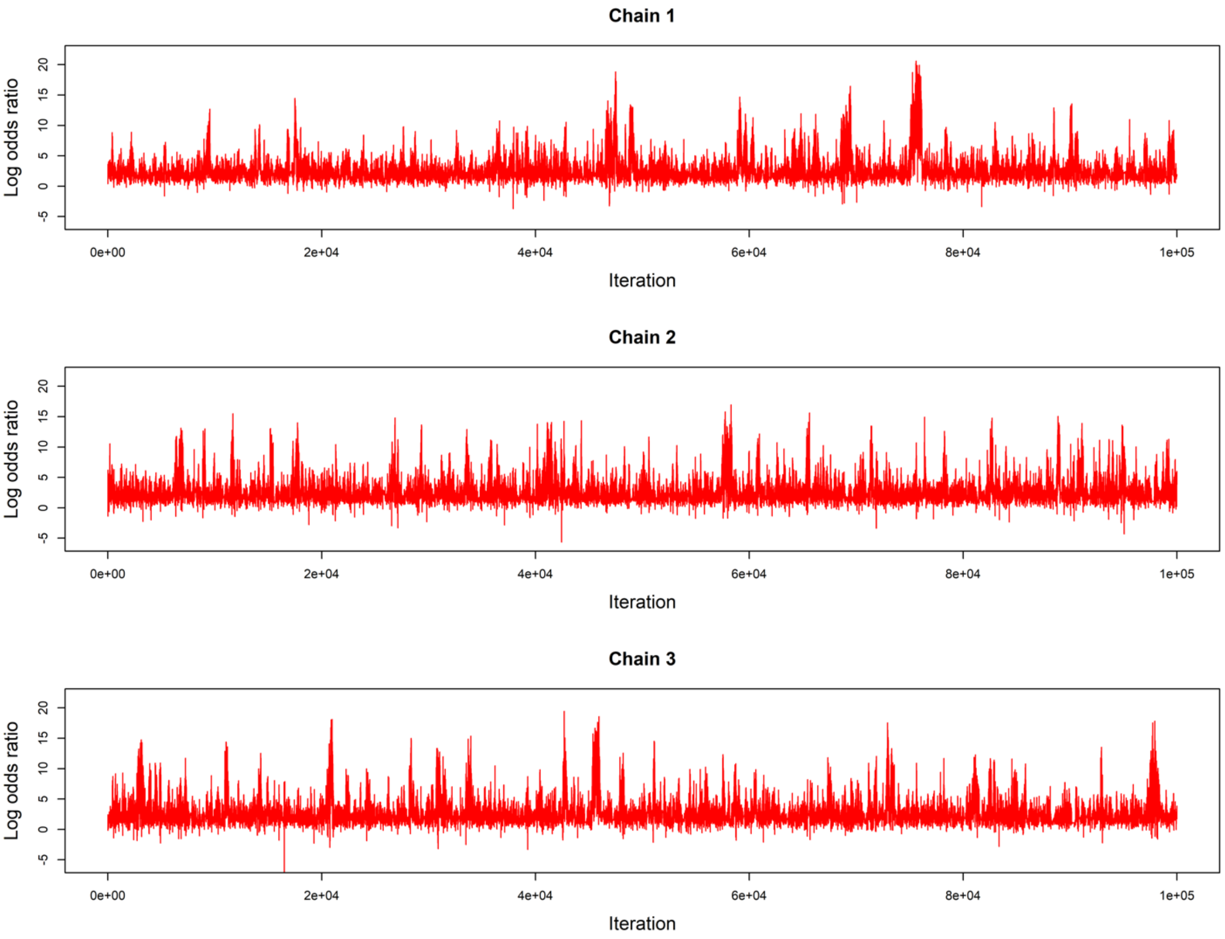

Figure S33. Trace plots of the overall log odds ratio based on the prior $U(0, 10)$ for τ in the meta-analysis on colitis (Example 3).

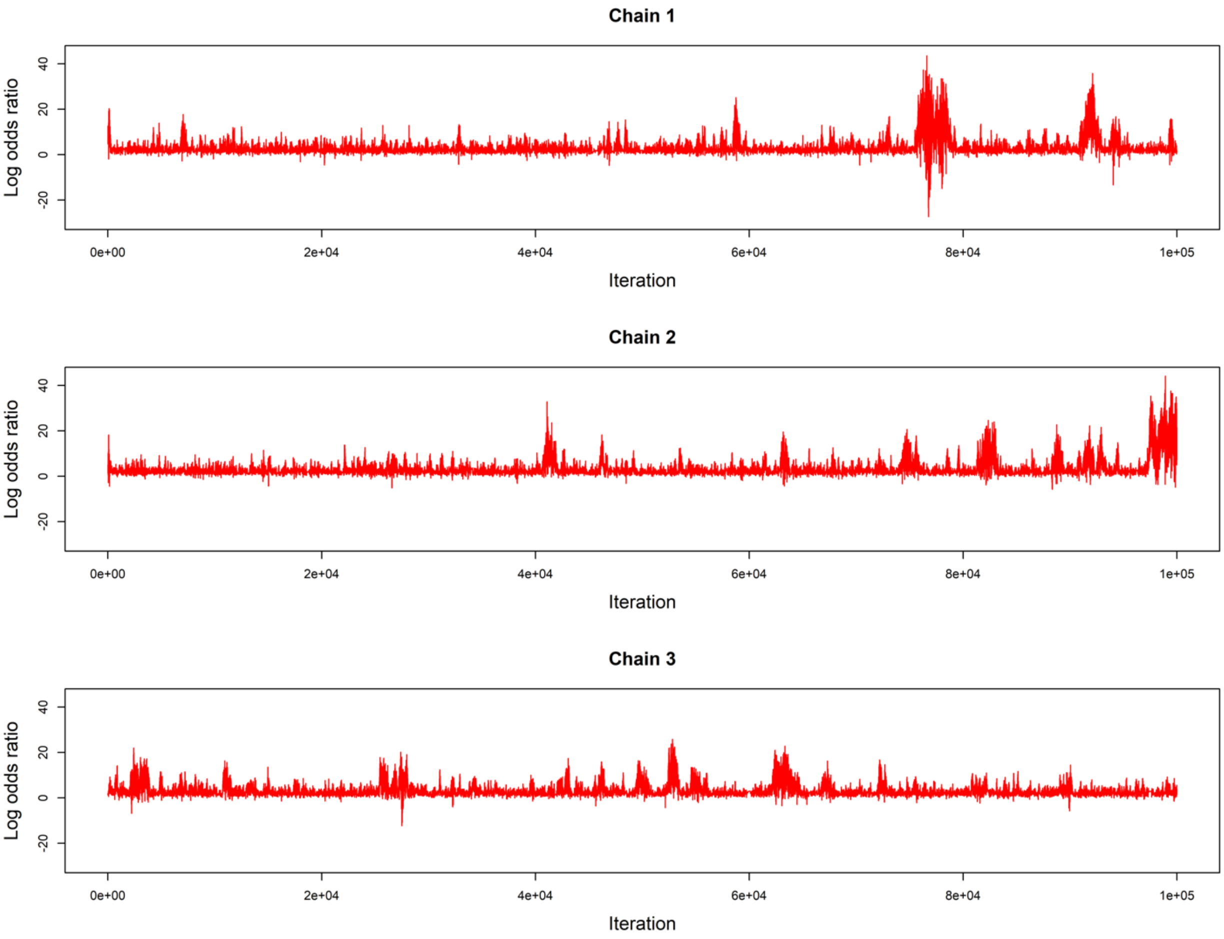

Figure S34. Trace plots of the overall log odds ratio based on the prior $U(0, 100)$ for τ in the meta-analysis on colitis (Example 3).

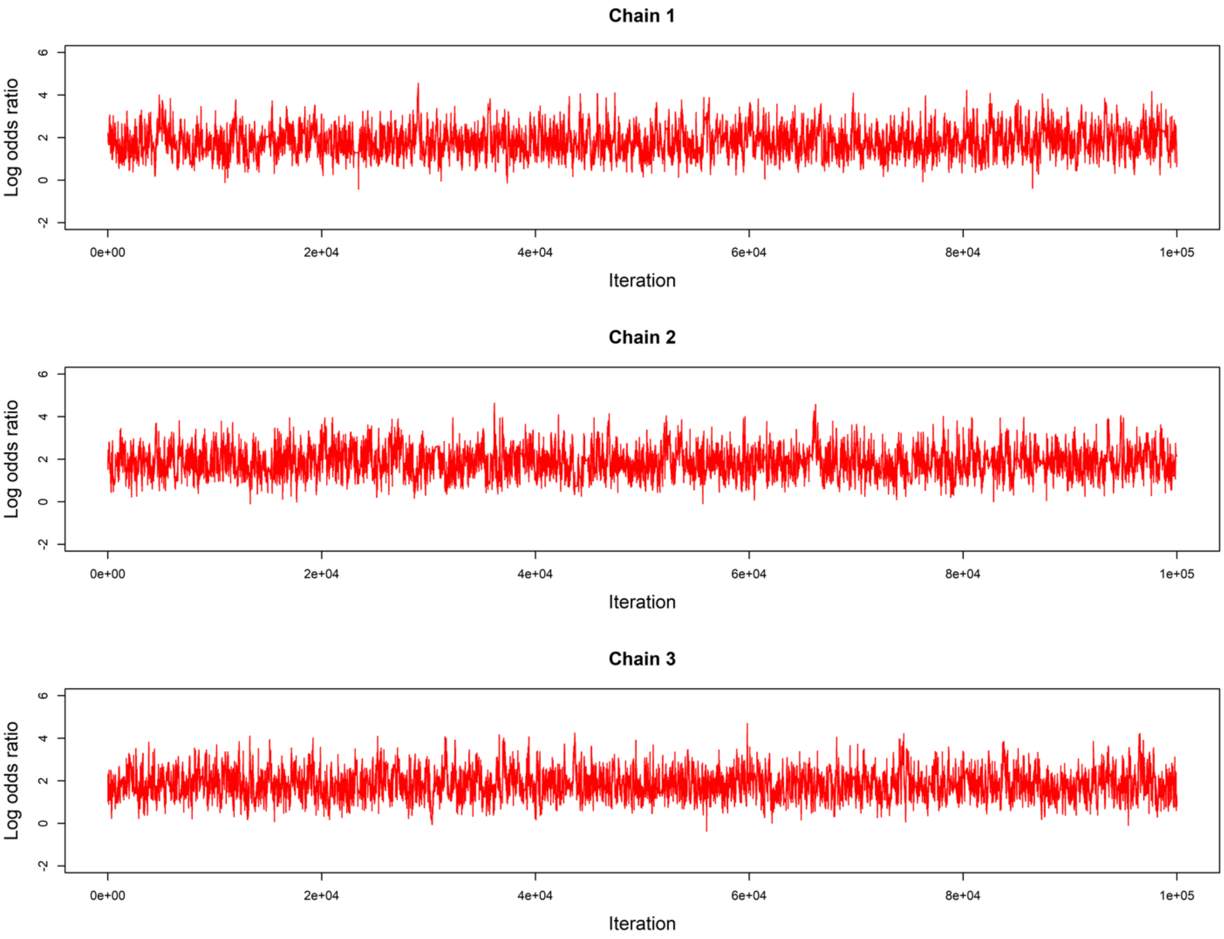

Figure S35. Trace plots of the overall log odds ratio based on the prior $HN(0, 0.1)$ for τ in the meta-analysis on colitis (Example 3).

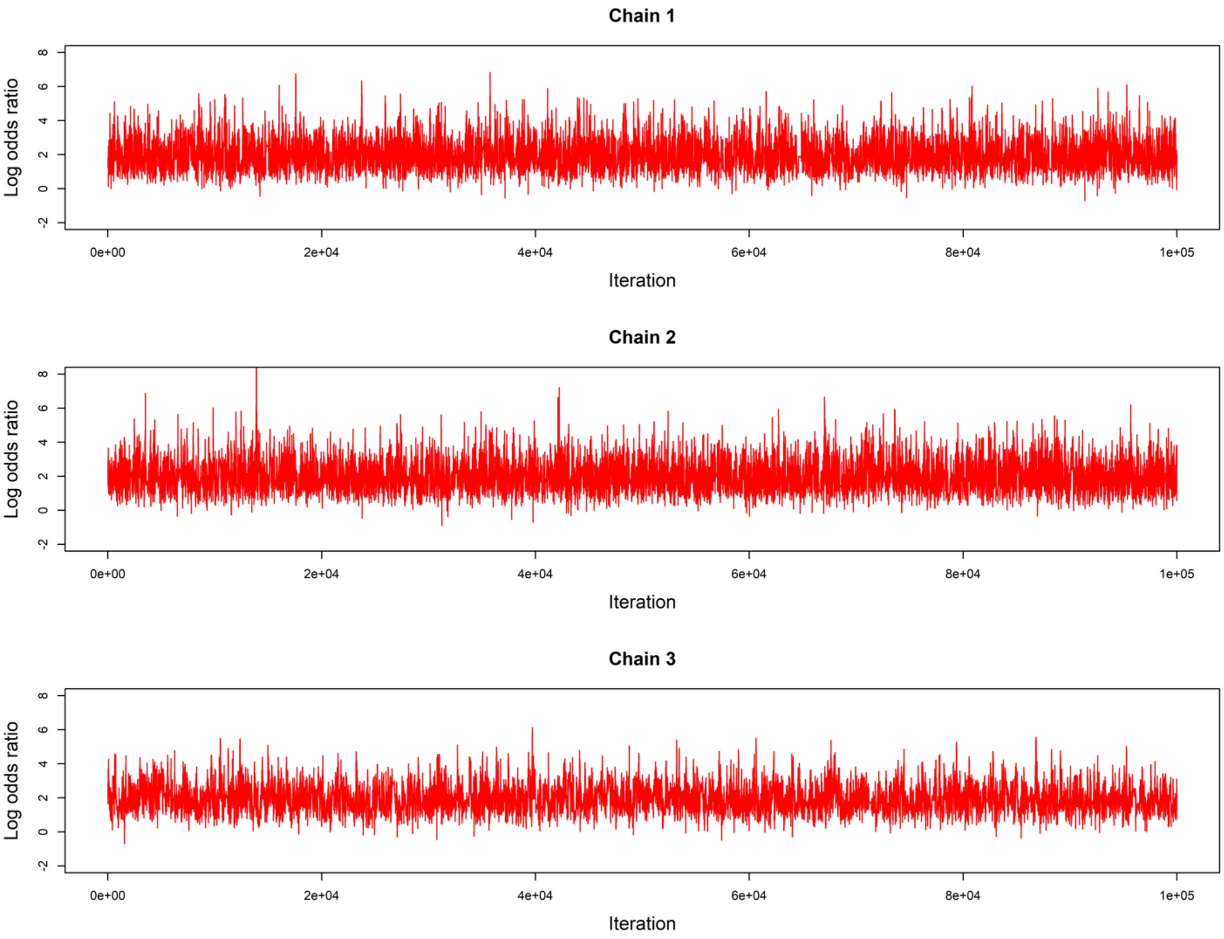

Figure S36. Trace plots of the overall log odds ratio based on the prior $HN(0, 1)$ for τ in the meta-analysis on colitis (Example 3).

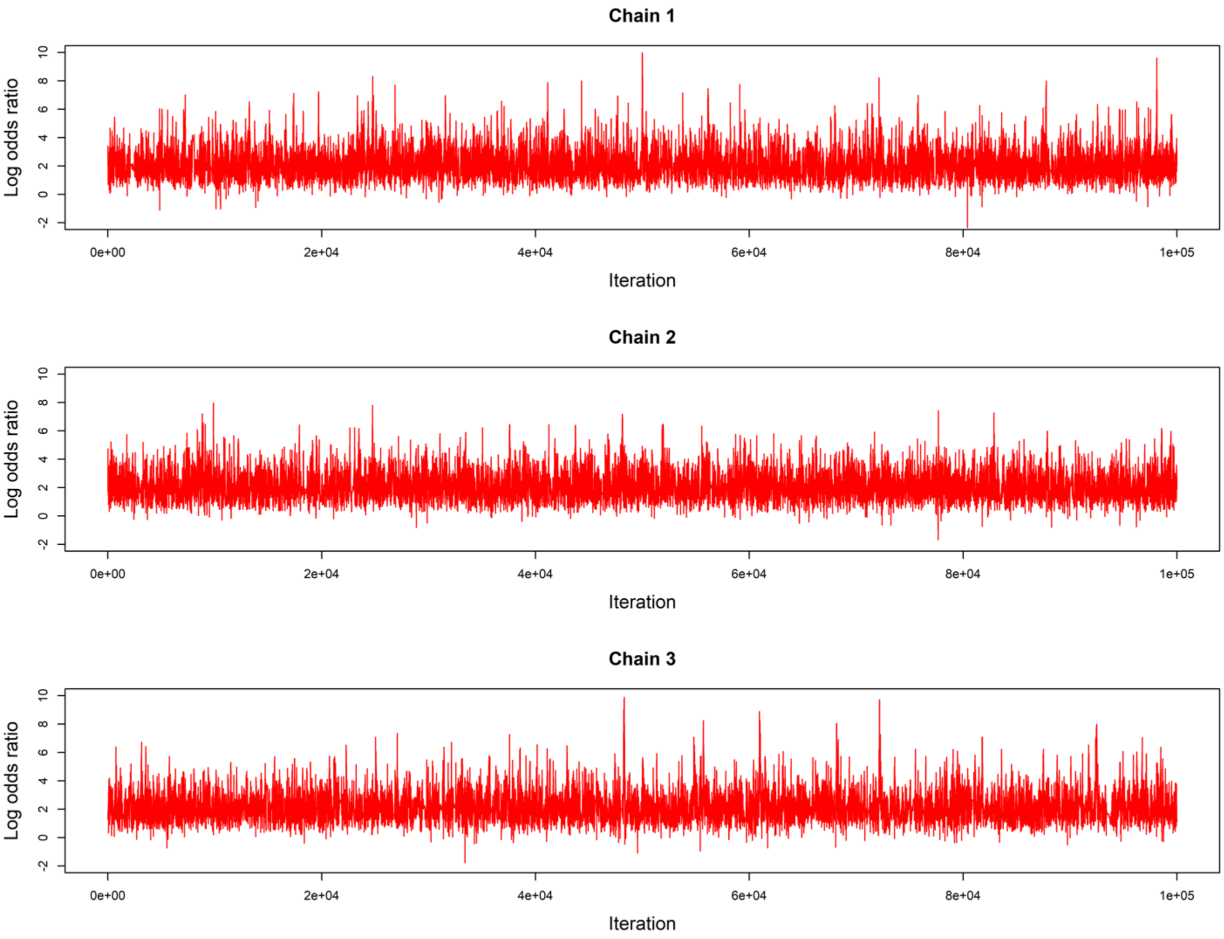

Figure S37. Trace plots of the overall log odds ratio based on the prior $HN(0, 2)$ for τ in the meta-analysis on colitis (Example 3).

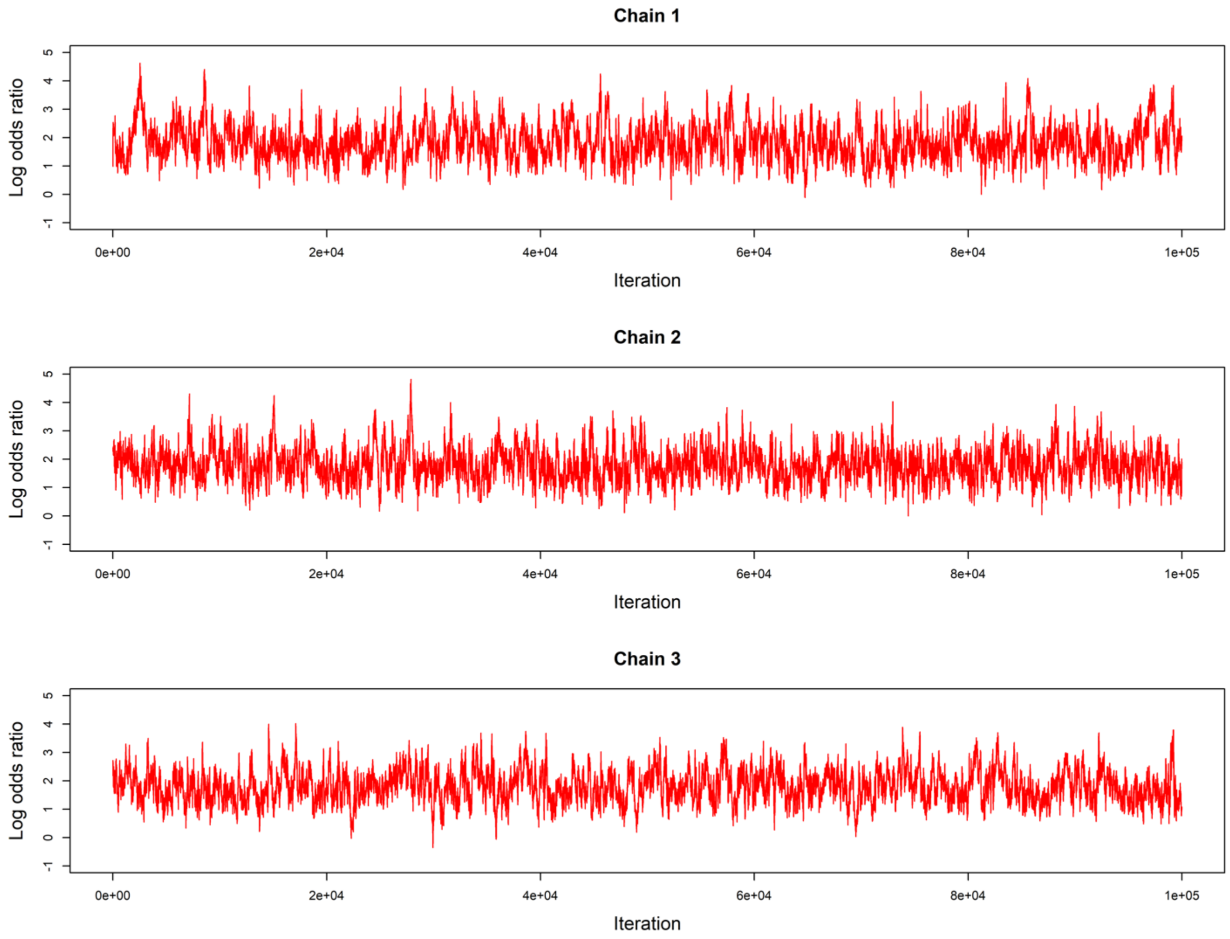

Figure S38. Trace plots of the overall log odds ratio based on the prior $LN(-4.06, 1.45^2)$ for τ^2 in the meta-analysis on colitis (Example 3).

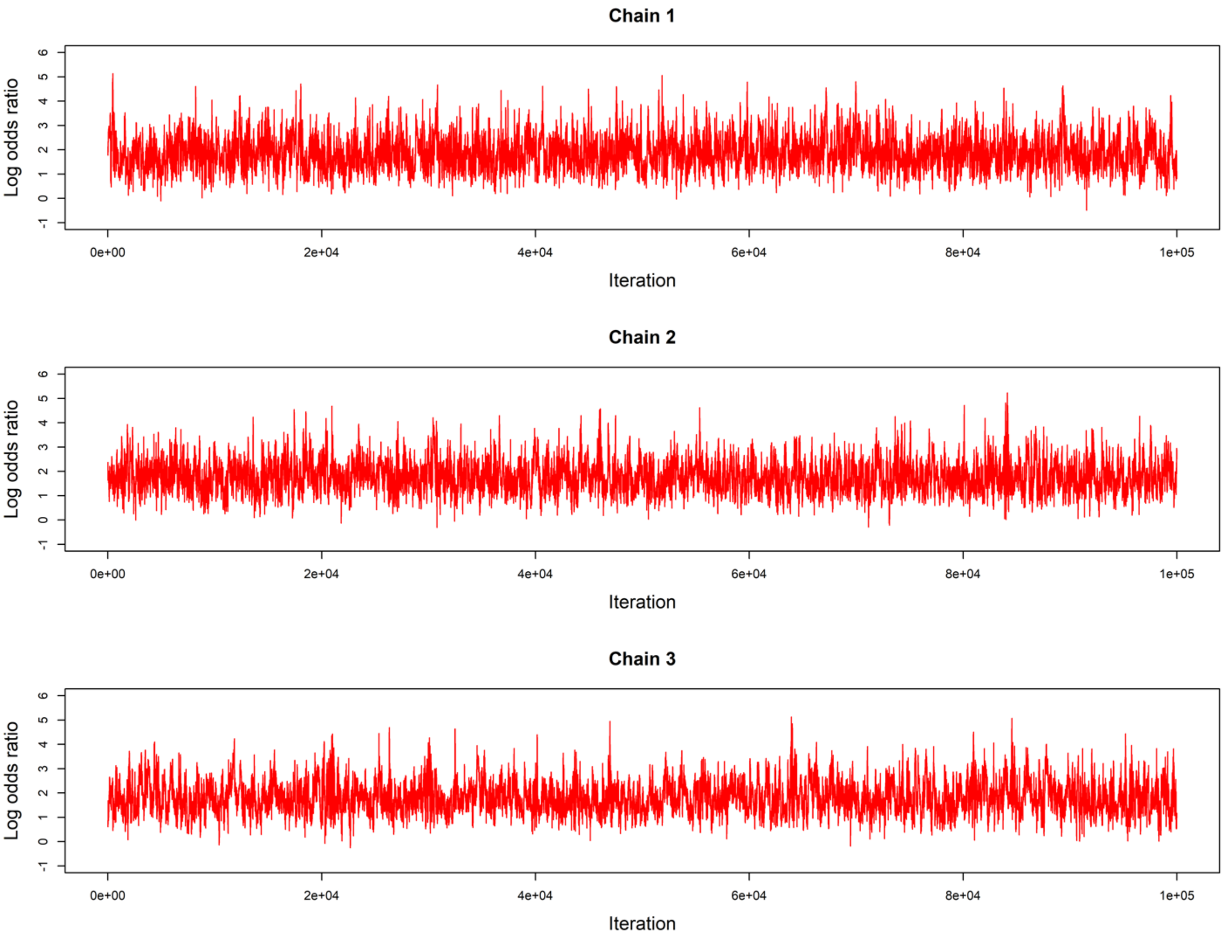

Figure S39. Trace plots of the overall log odds ratio based on the prior $LN(-3.02, 1.85^2)$ for τ^2 in the meta-analysis on colitis (Example 3).

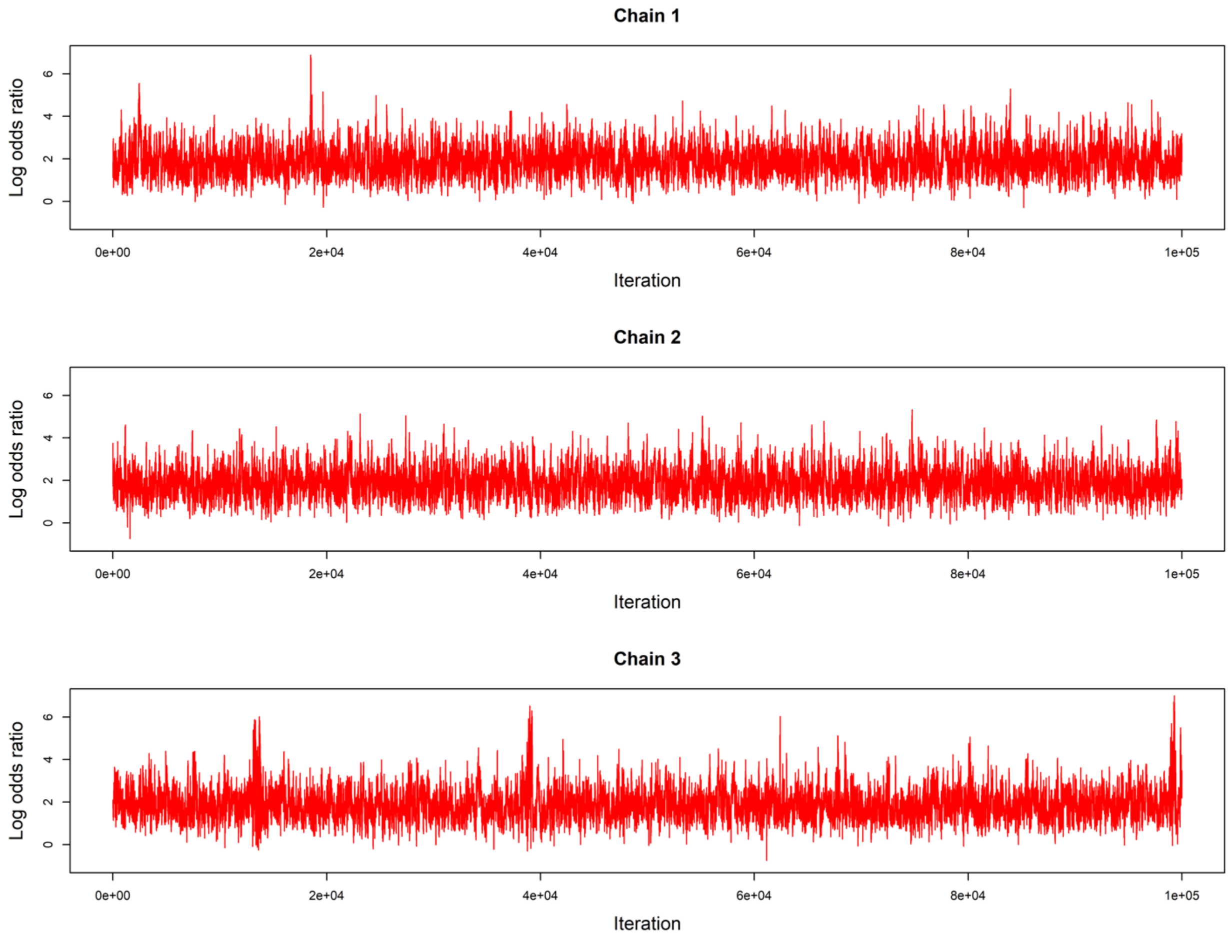

Figure S40. Trace plots of the overall log odds ratio based on the prior $LN(-2.13, 1.58^2)$ for τ^2 in the meta-analysis on colitis (Example 3).

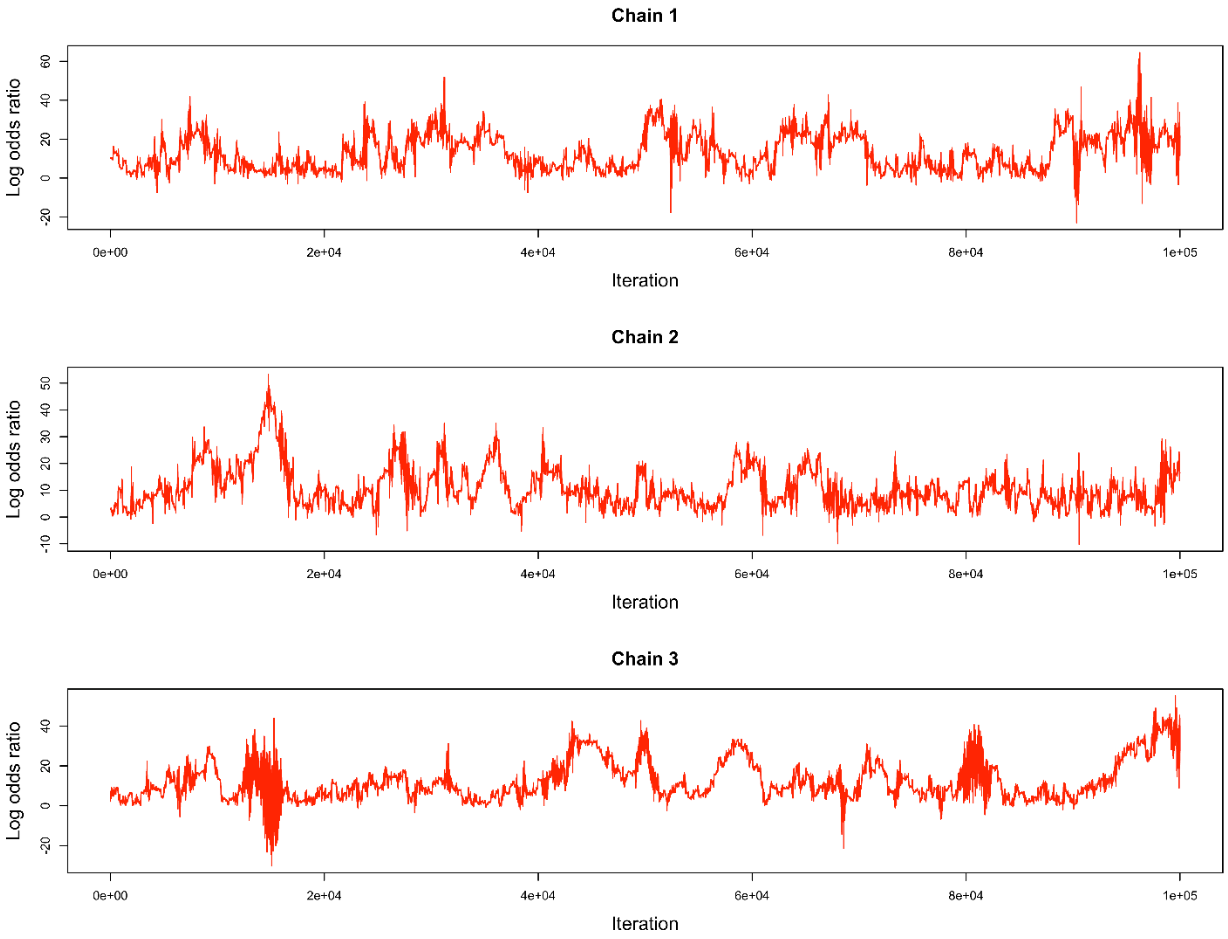

Figure S41. Trace plots of the overall log odds ratio based on the prior $IG(0.001, 0.001)$ for τ^2 in the meta-analysis on hepatitis (Example 4).

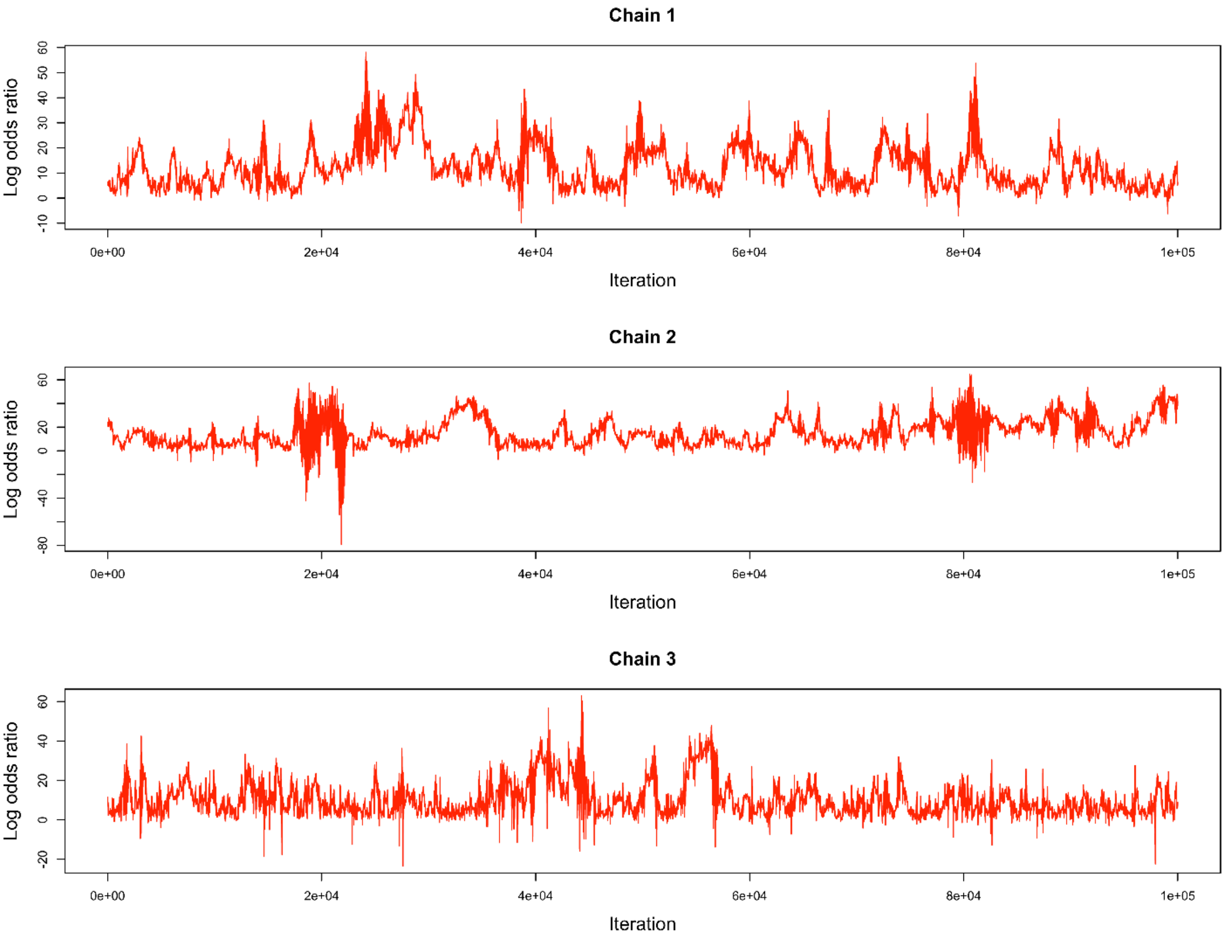

Figure S42. Trace plots of the overall log odds ratio based on the prior $IG(0.01, 0.01)$ for τ^2 in the meta-analysis on hepatitis (Example 4).

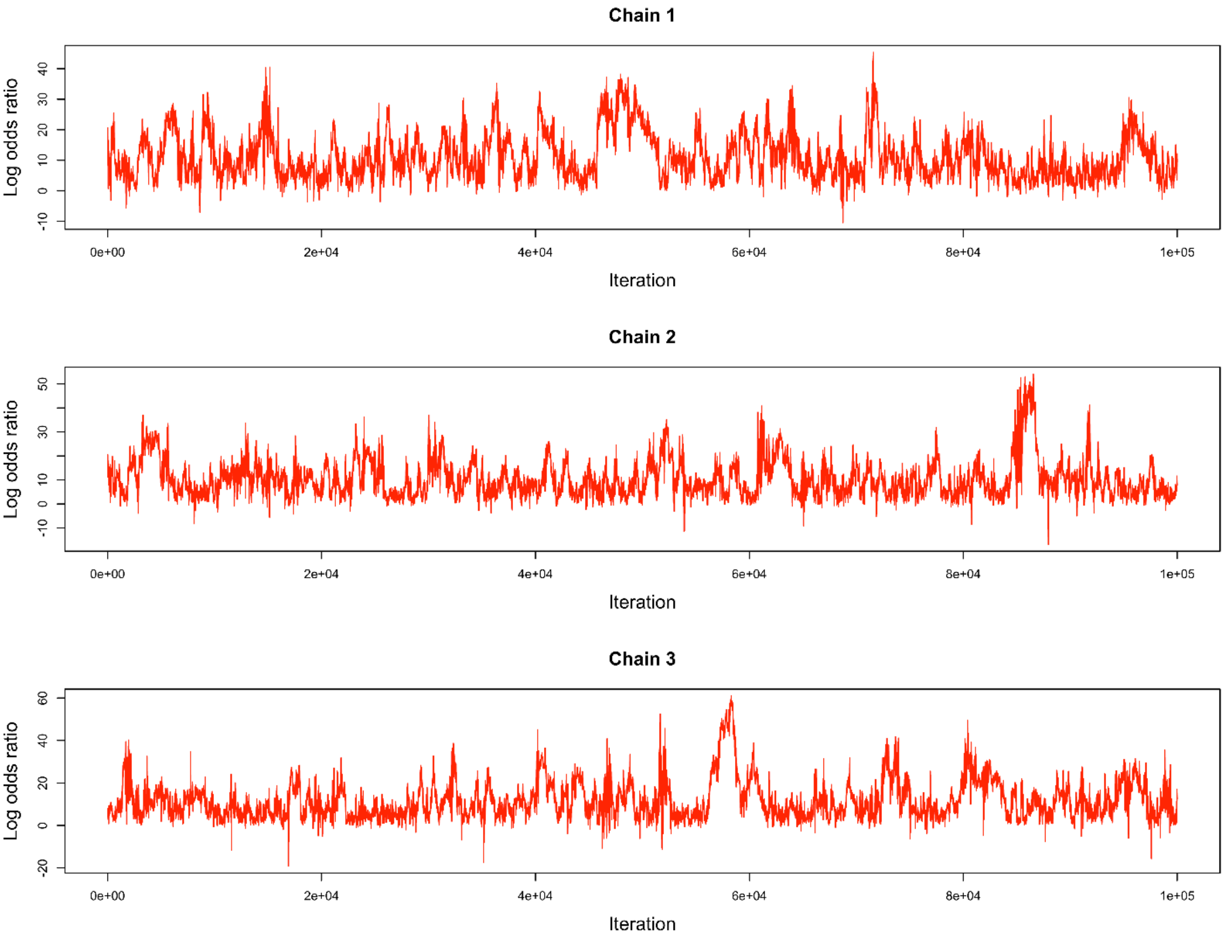

Figure S43. Trace plots of the overall log odds ratio based on the prior $IG(0.1, 0.1)$ for τ^2 in the meta-analysis on hepatitis (Example 4).

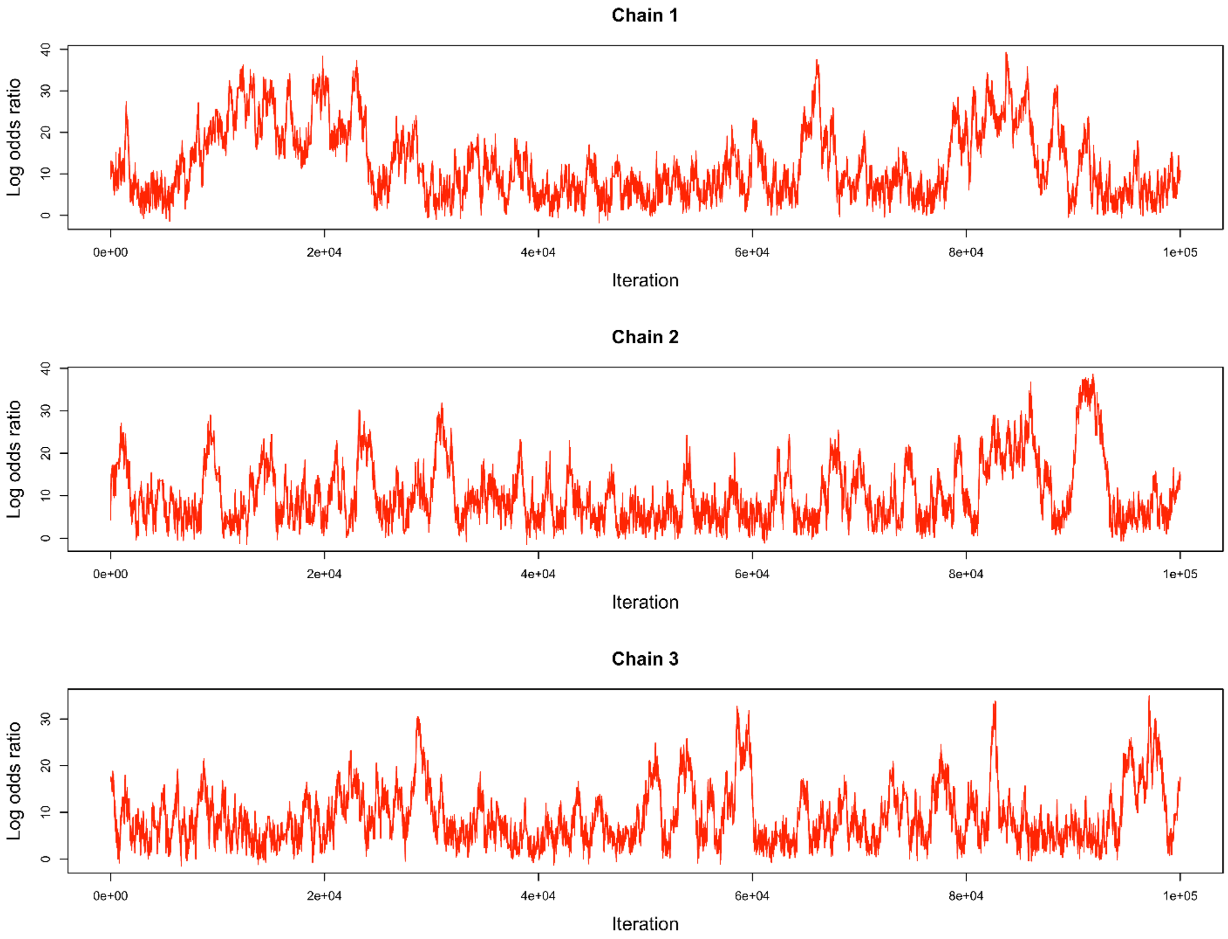

Figure S44. Trace plots of the overall log odds ratio based on the prior $U(0, 2)$ for τ in the meta-analysis on hepatitis (Example 4).

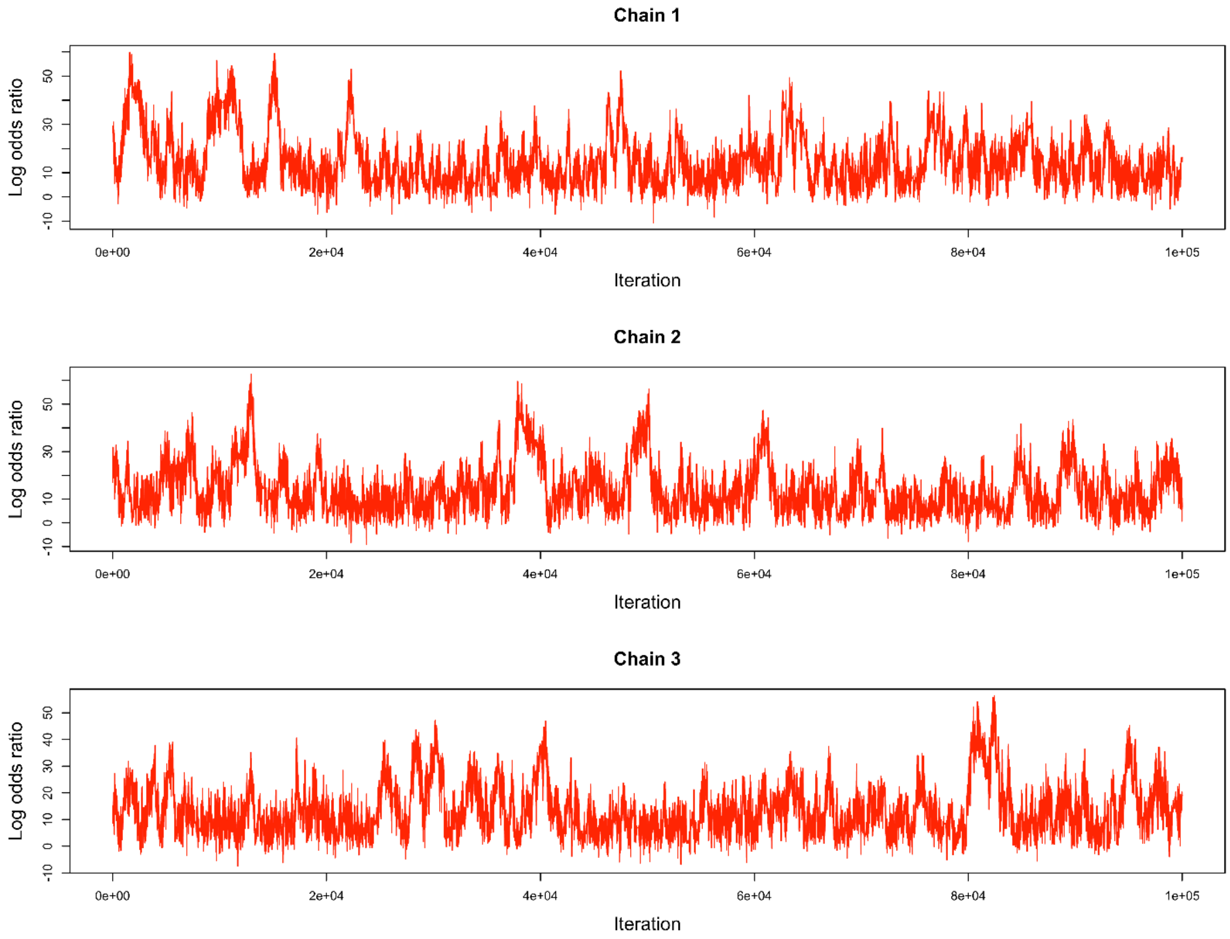

Figure S45. Trace plots of the overall log odds ratio based on the prior $U(0, 10)$ for τ in the meta-analysis on hepatitis (Example 4).

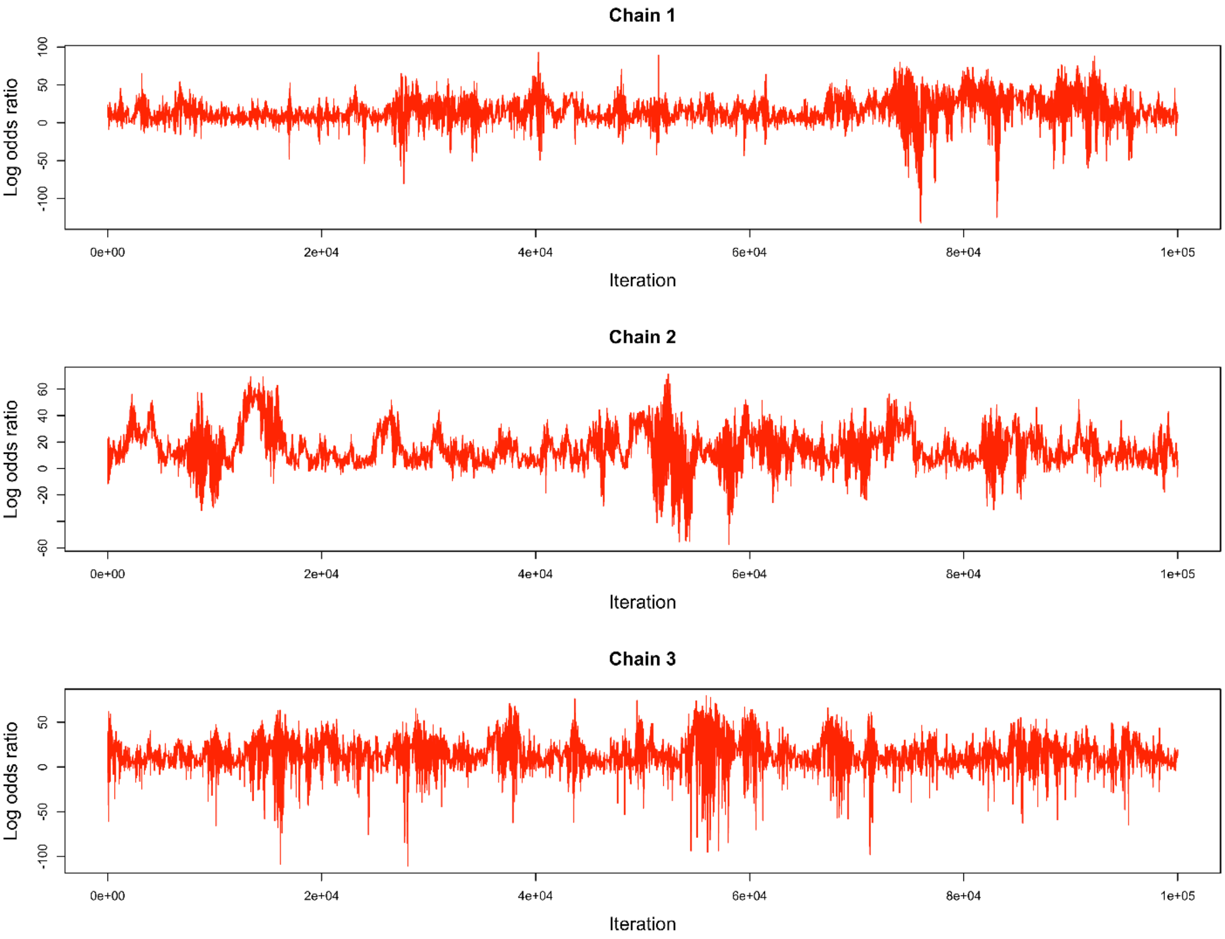

Figure S46. Trace plots of the overall log odds ratio based on the prior $U(0, 100)$ for τ in the meta-analysis on hepatitis (Example 4).

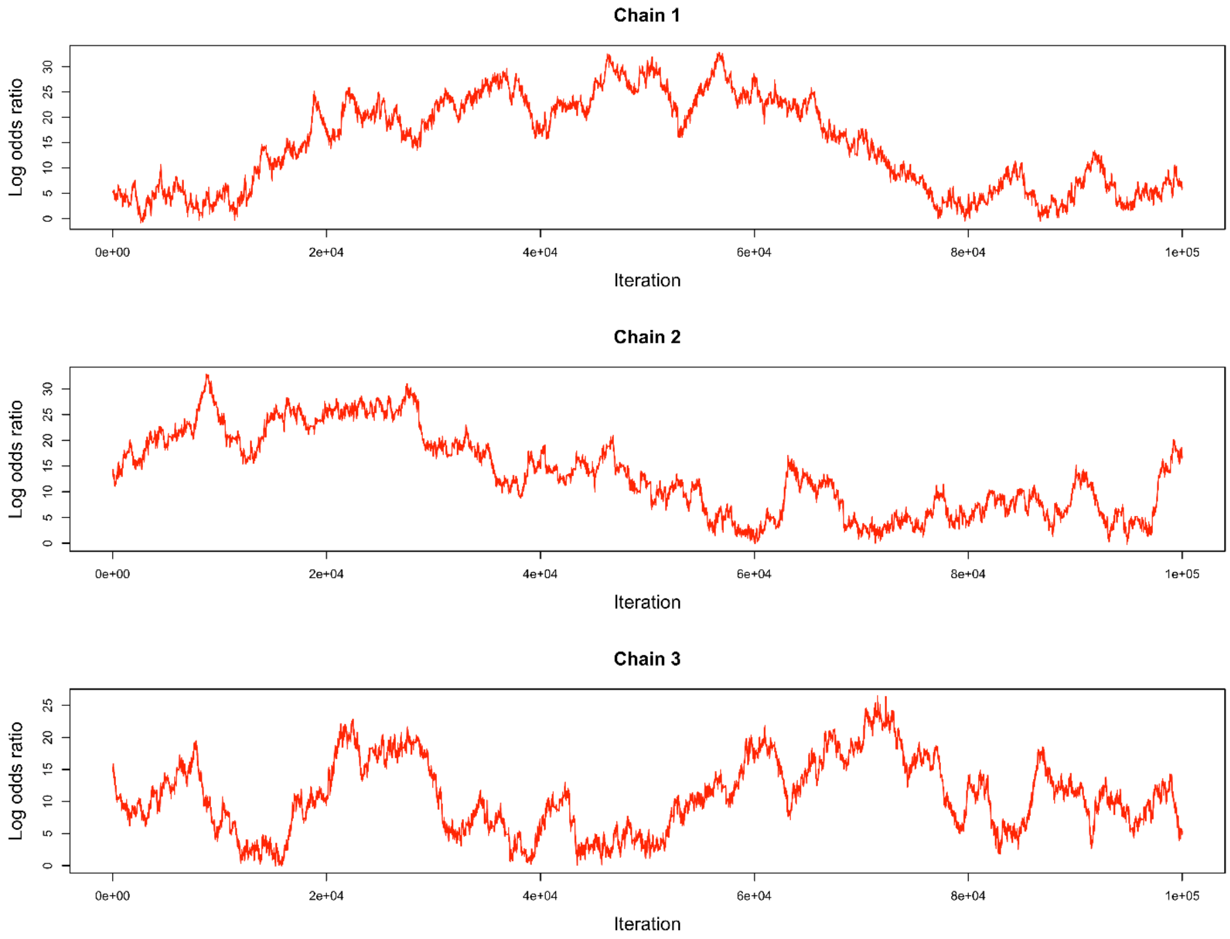

Figure S47. Trace plots of the overall log odds ratio based on the prior $HN(0, 0.1)$ for τ in the meta-analysis on hepatitis (Example 4).

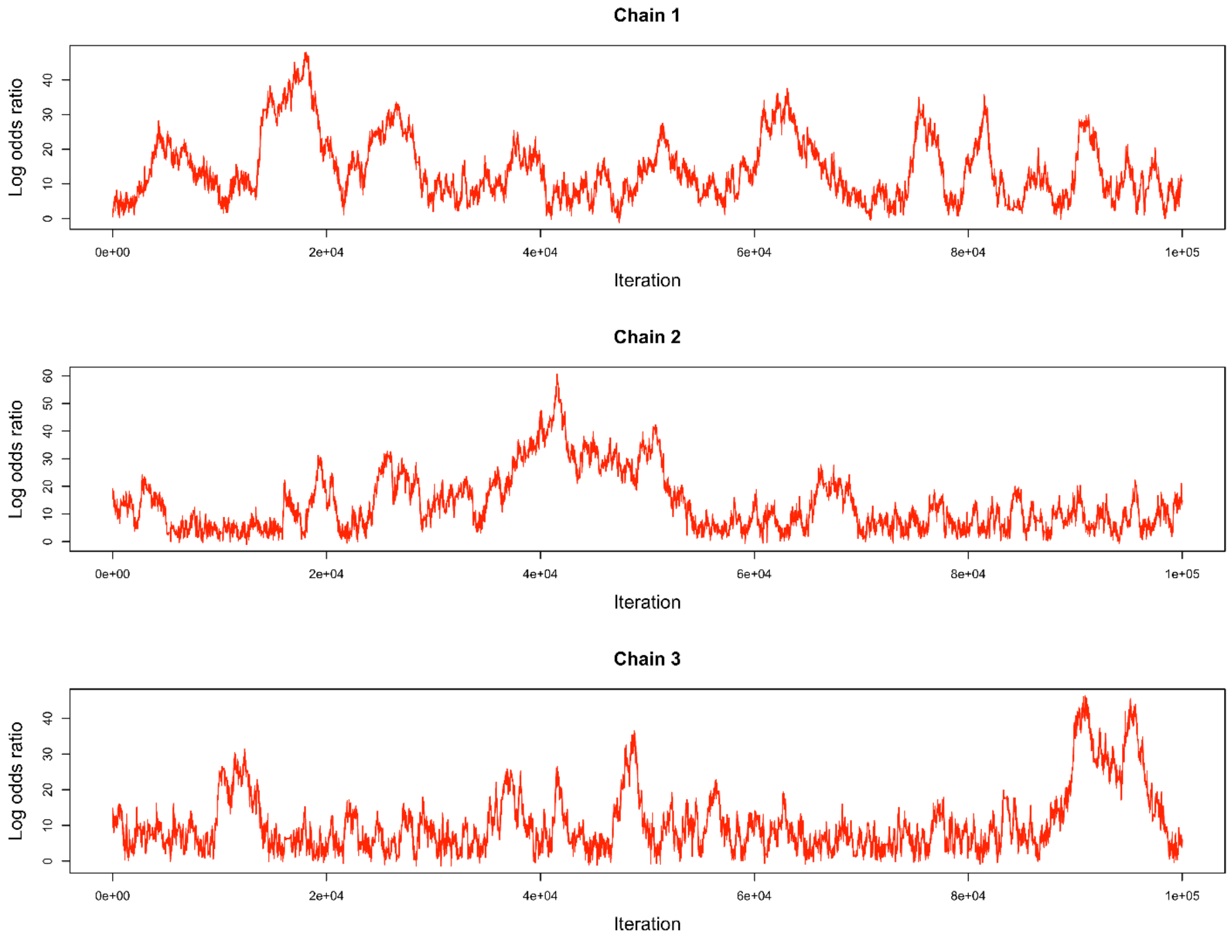

Figure S48. Trace plots of the overall log odds ratio based on the prior $HN(0, 1)$ for τ in the meta-analysis on hepatitis (Example 4).

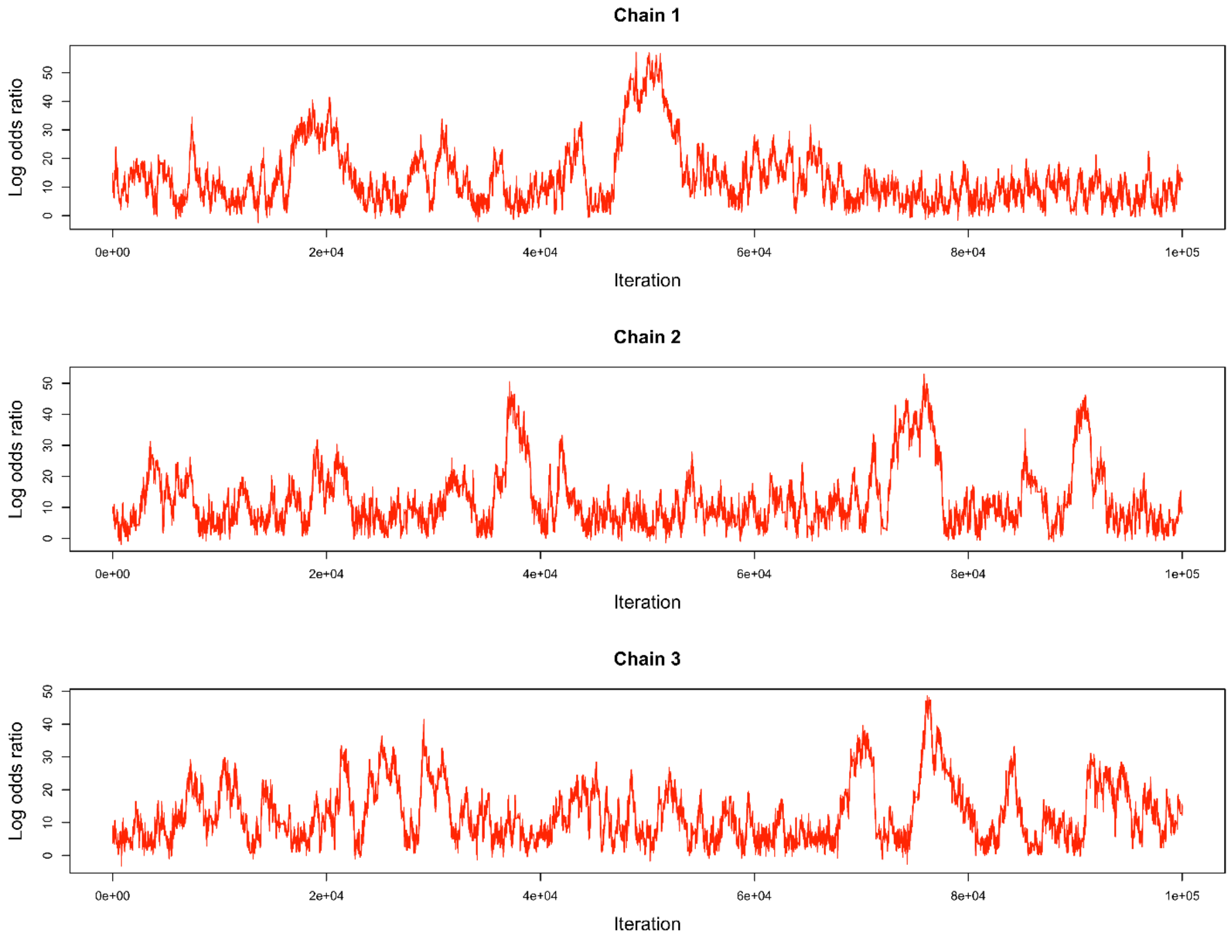

Figure S49. Trace plots of the overall log odds ratio based on the prior $HN(0, 2)$ for τ in the meta-analysis on hepatitis (Example 4).

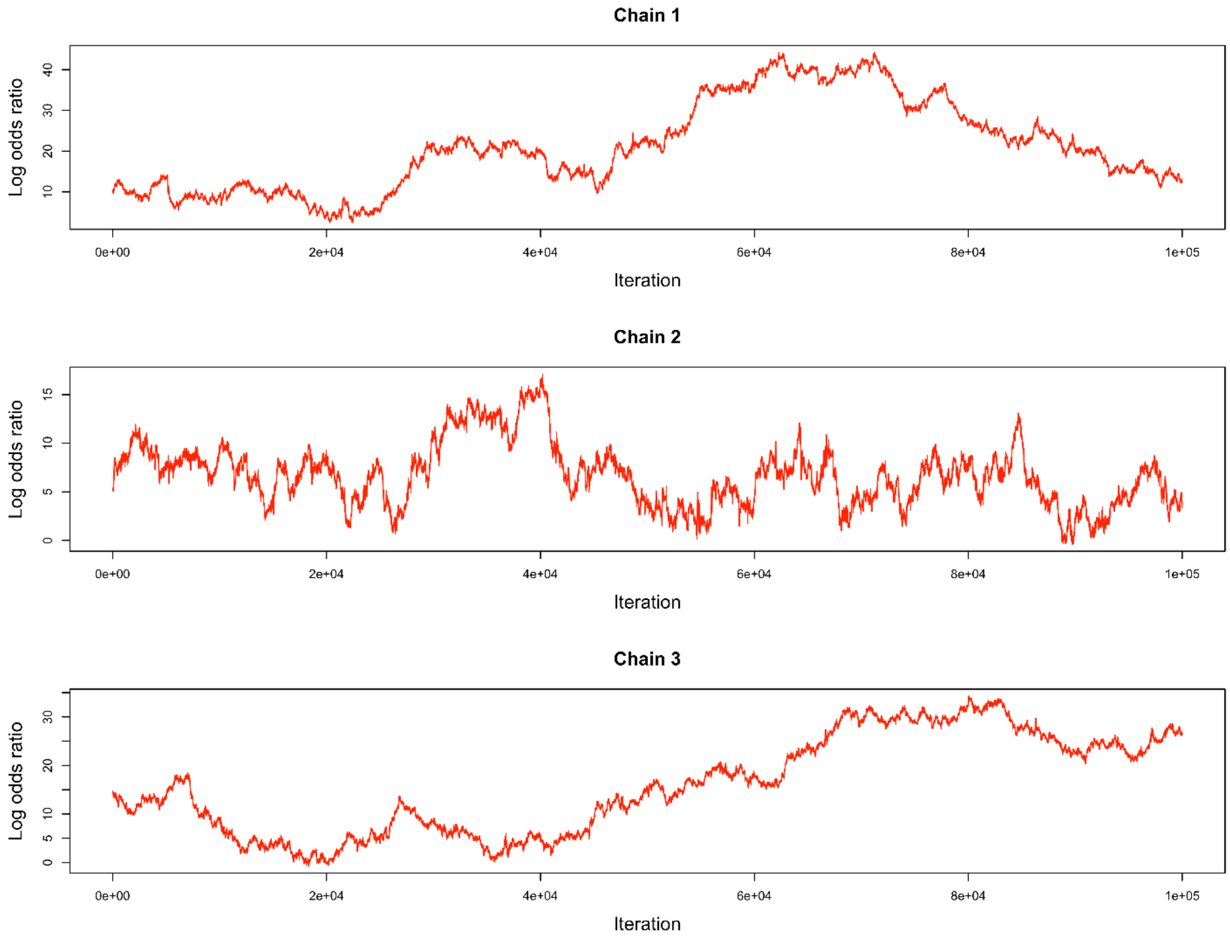

Figure S50. Trace plots of the overall log odds ratio based on the prior $LN(-4.06, 1.45^2)$ for τ^2 in the meta-analysis on hepatitis (Example 4).

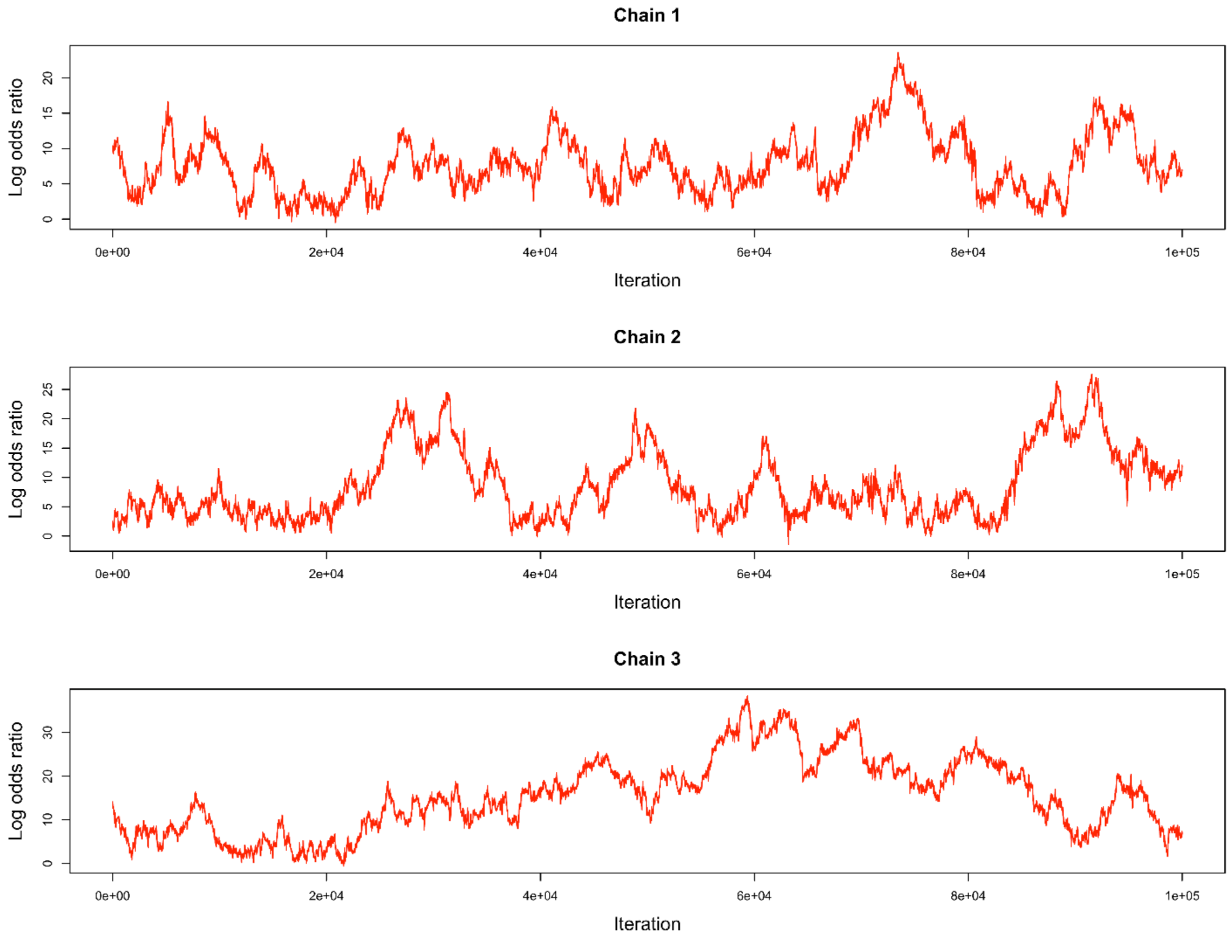

Figure S51. Trace plots of the overall log odds ratio based on the prior $LN(-3.02, 1.85^2)$ for τ^2 in the meta-analysis on hepatitis (Example 4).

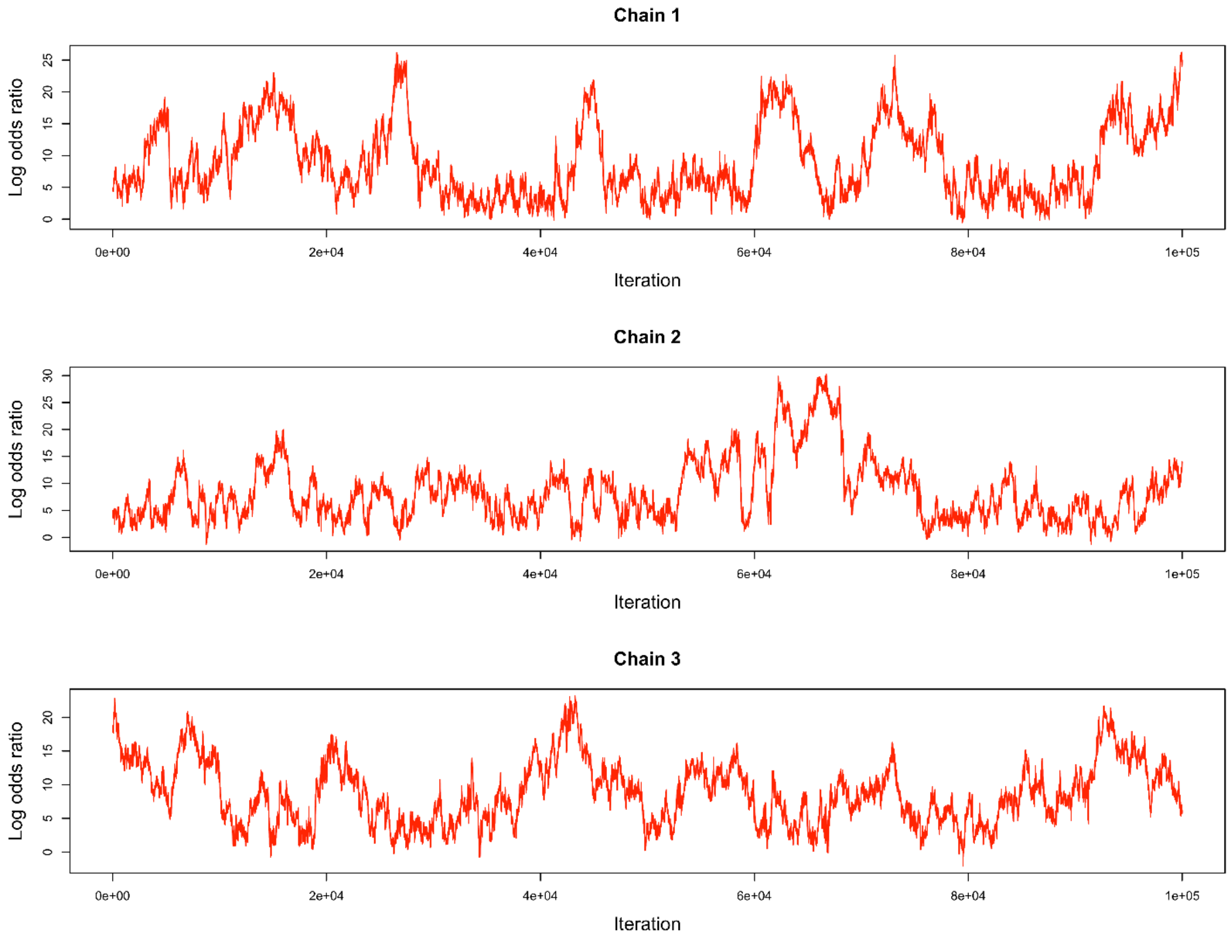

Figure S52. Trace plots of the overall log odds ratio based on the prior $LN(-2.13, 1.58^2)$ for τ^2 in the meta-analysis on hepatitis (Example 4).

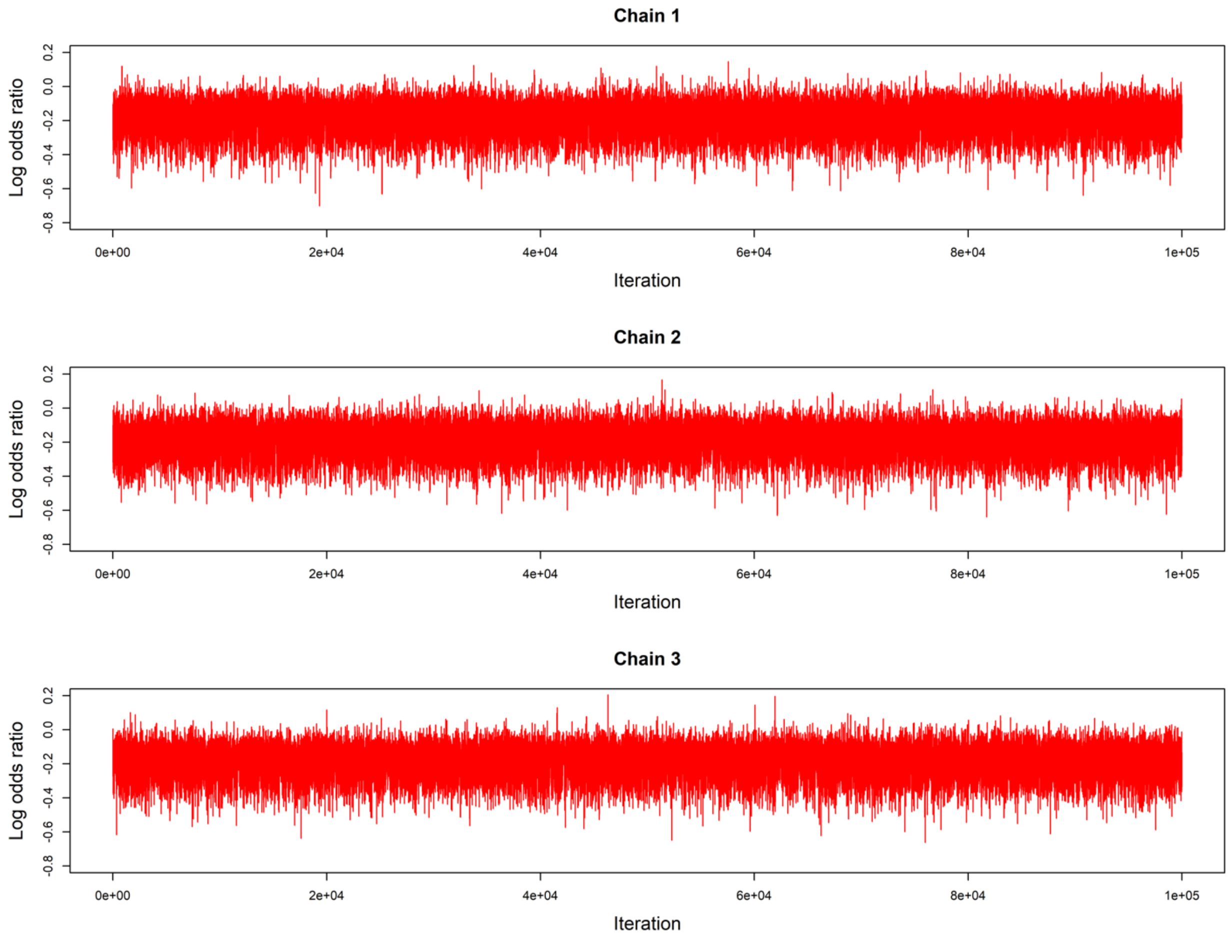

Figure S53. Trace plots of the overall log odds ratio based on the prior $IG(0.001, 0.001)$ for τ^2 in the meta-analysis on acute respiratory tract infection (Example 5).

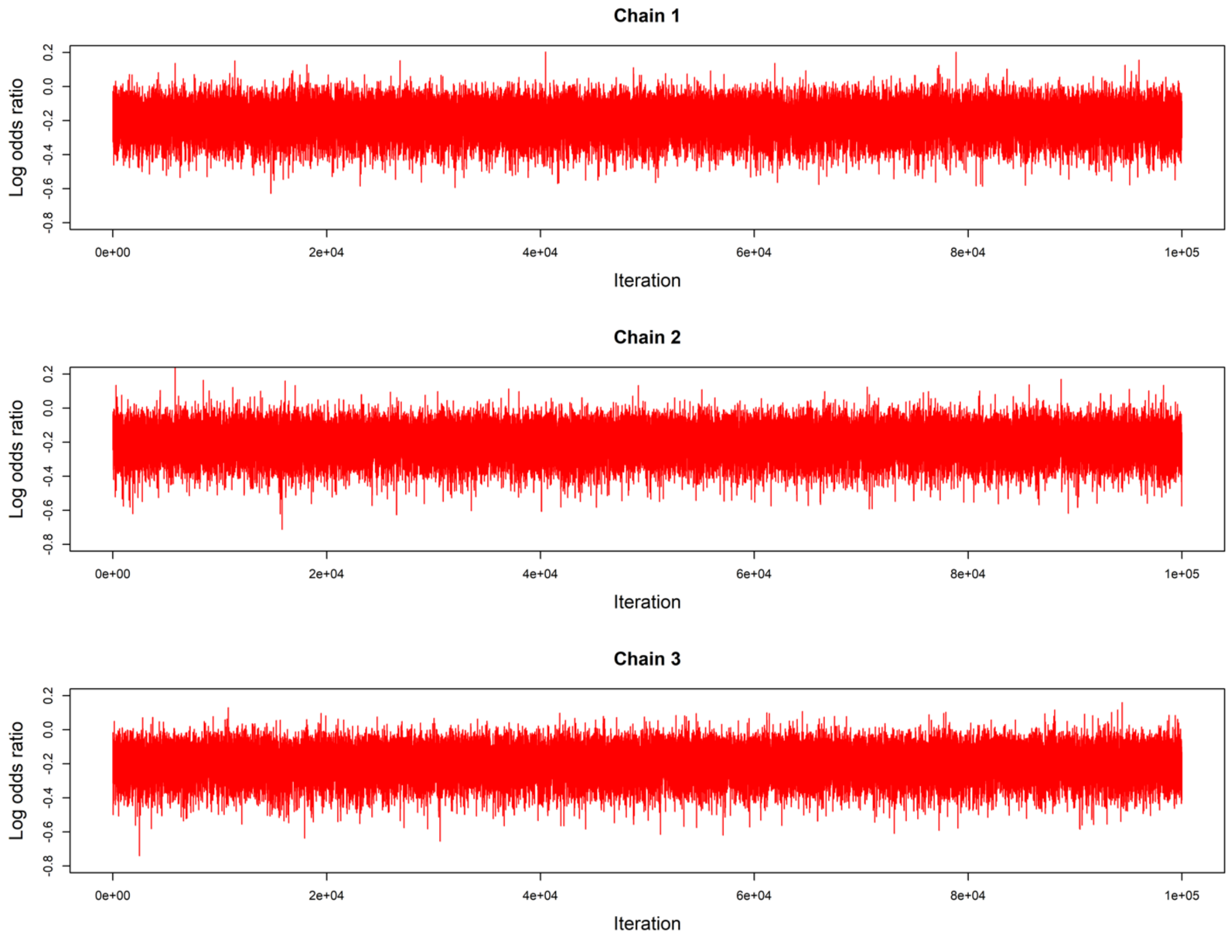

Figure S54. Trace plots of the overall log odds ratio based on the prior $IG(0.01, 0.01)$ for τ^2 in the meta-analysis on acute respiratory tract infection (Example 5).

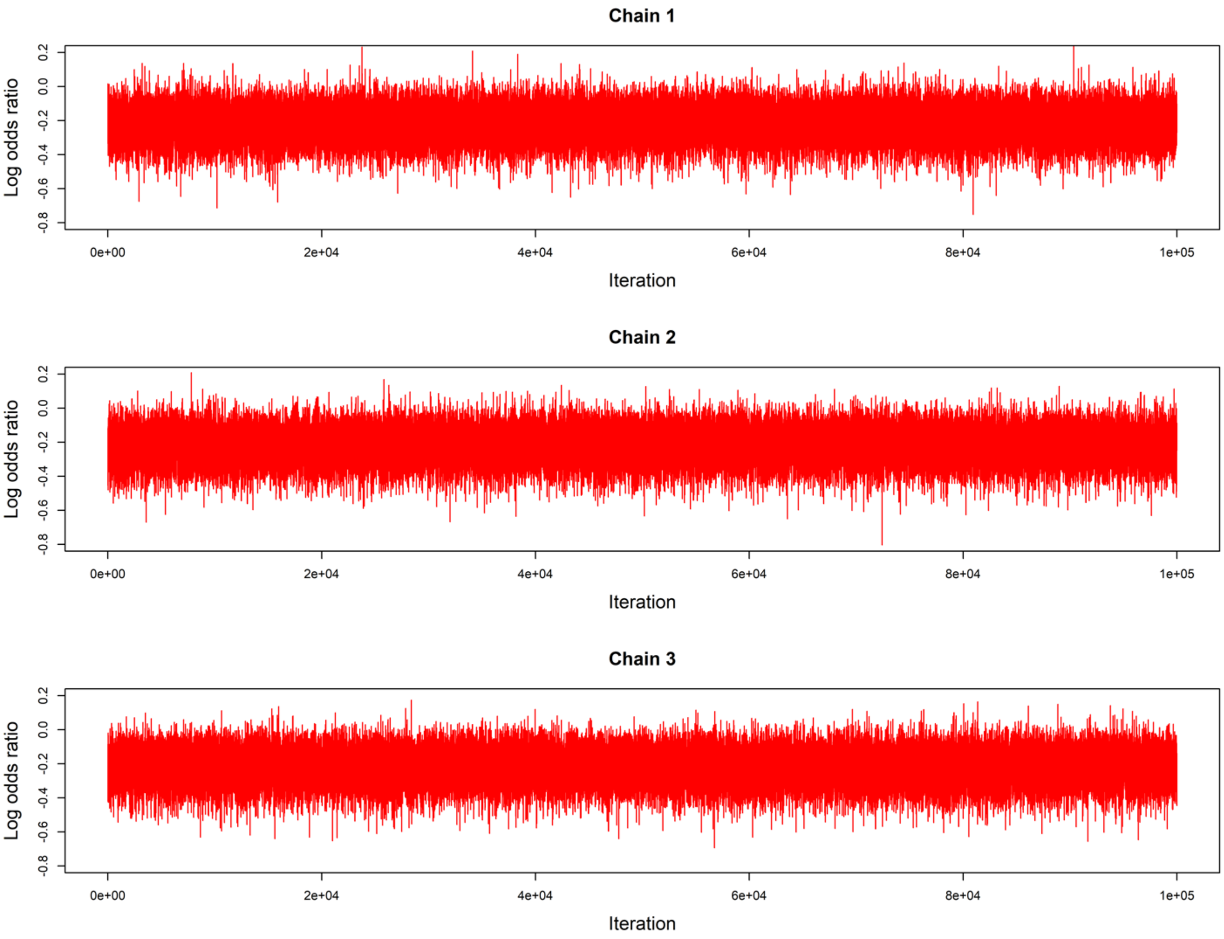

Figure S55. Trace plots of the overall log odds ratio based on the prior $IG(0.1, 0.1)$ for τ^2 in the meta-analysis on acute respiratory tract infection (Example 5).

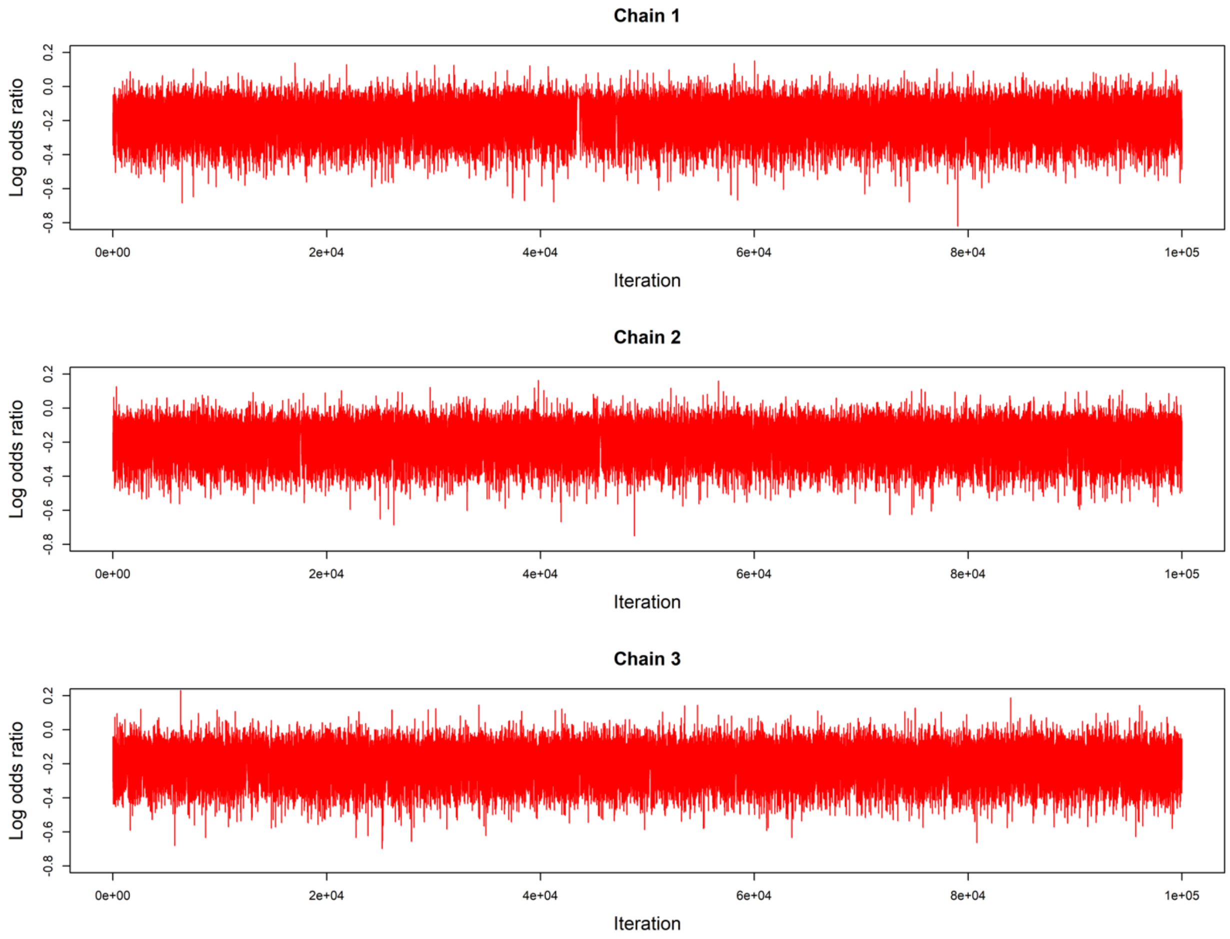

Figure S56. Trace plots of the overall log odds ratio based on the prior $U(0, 2)$ for τ in the meta-analysis on acute respiratory tract infection (Example 5).

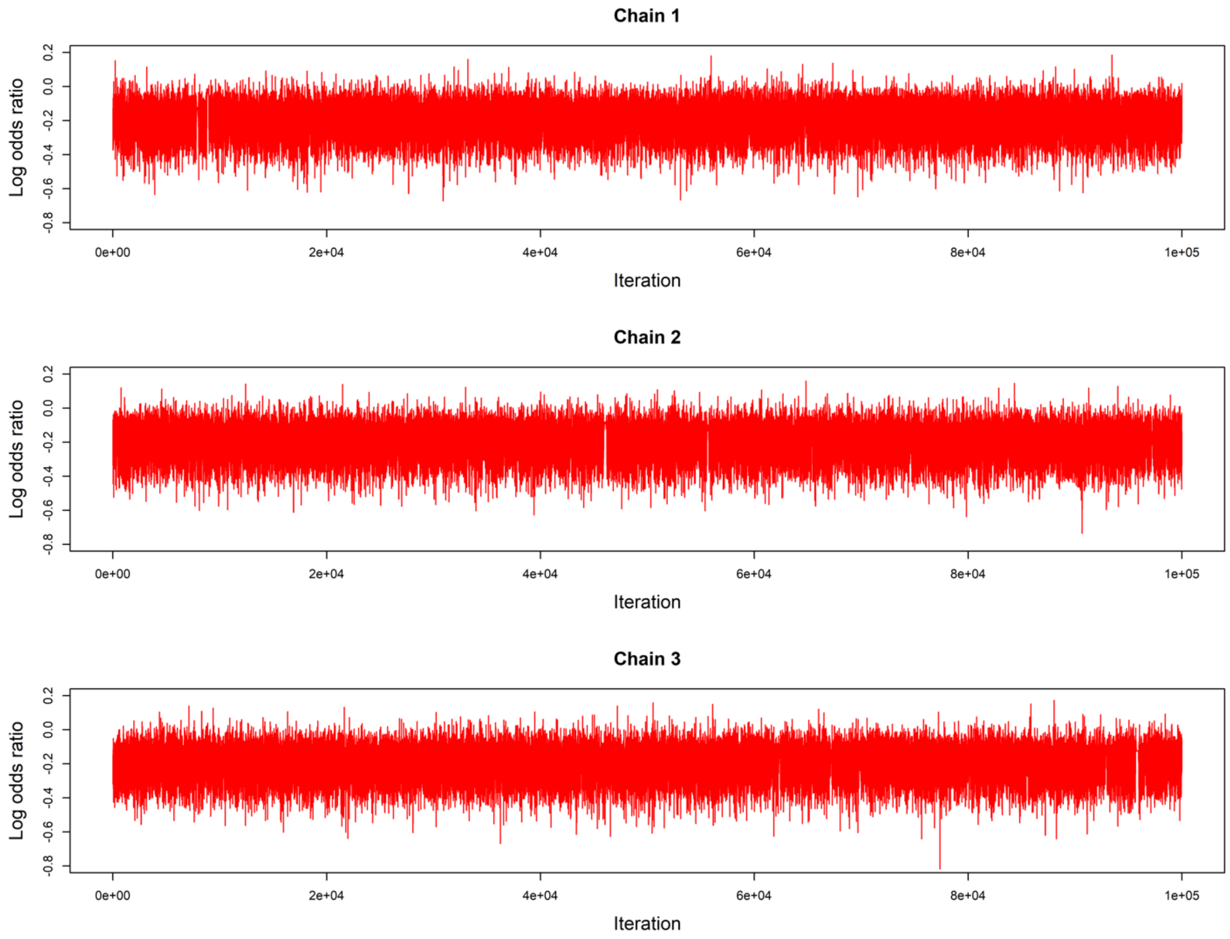

Figure S57. Trace plots of the overall log odds ratio based on the prior $U(0, 10)$ for τ in the meta-analysis on acute respiratory tract infection (Example 5).

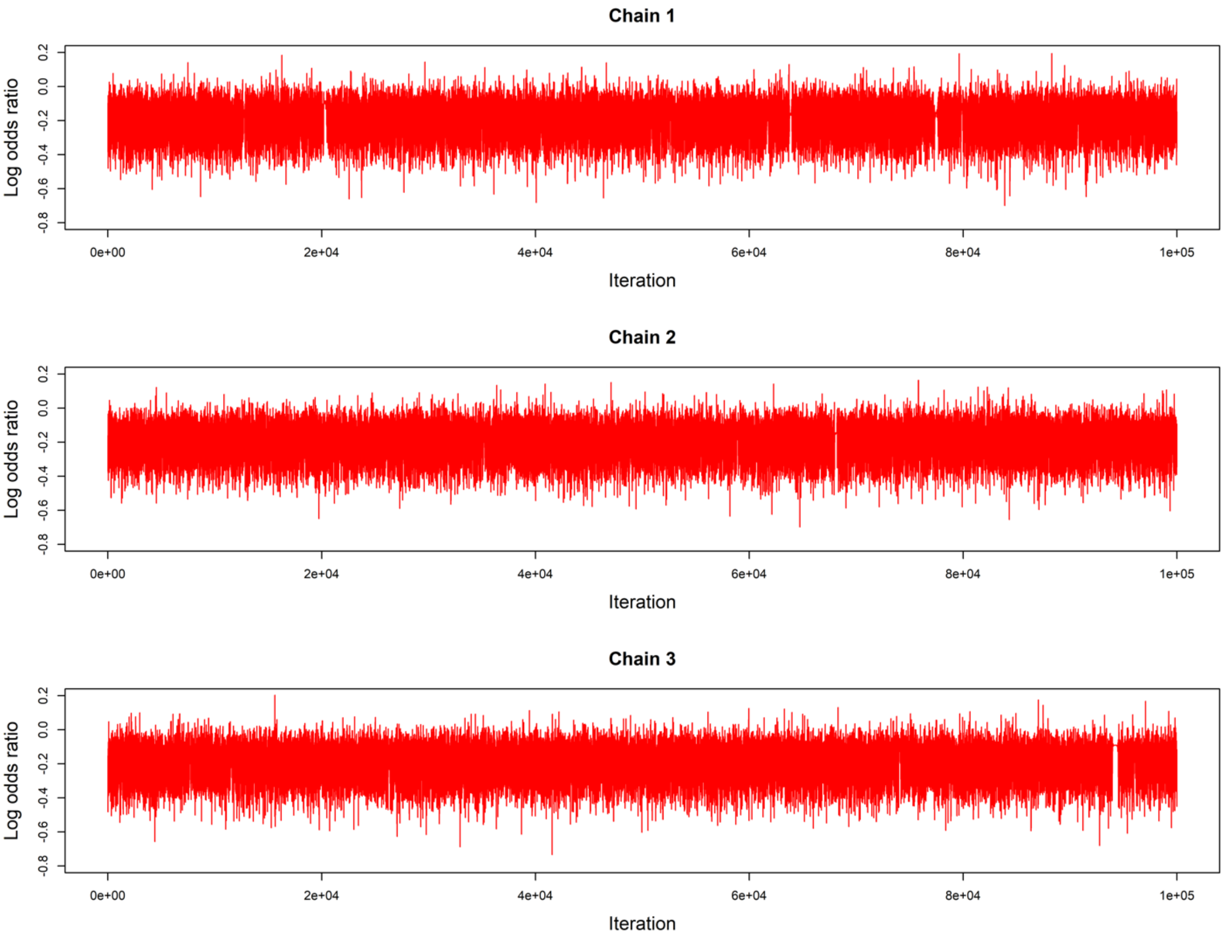

Figure S58. Trace plots of the overall log odds ratio based on the prior $U(0, 100)$ for τ in the meta-analysis on acute respiratory tract infection (Example 5).

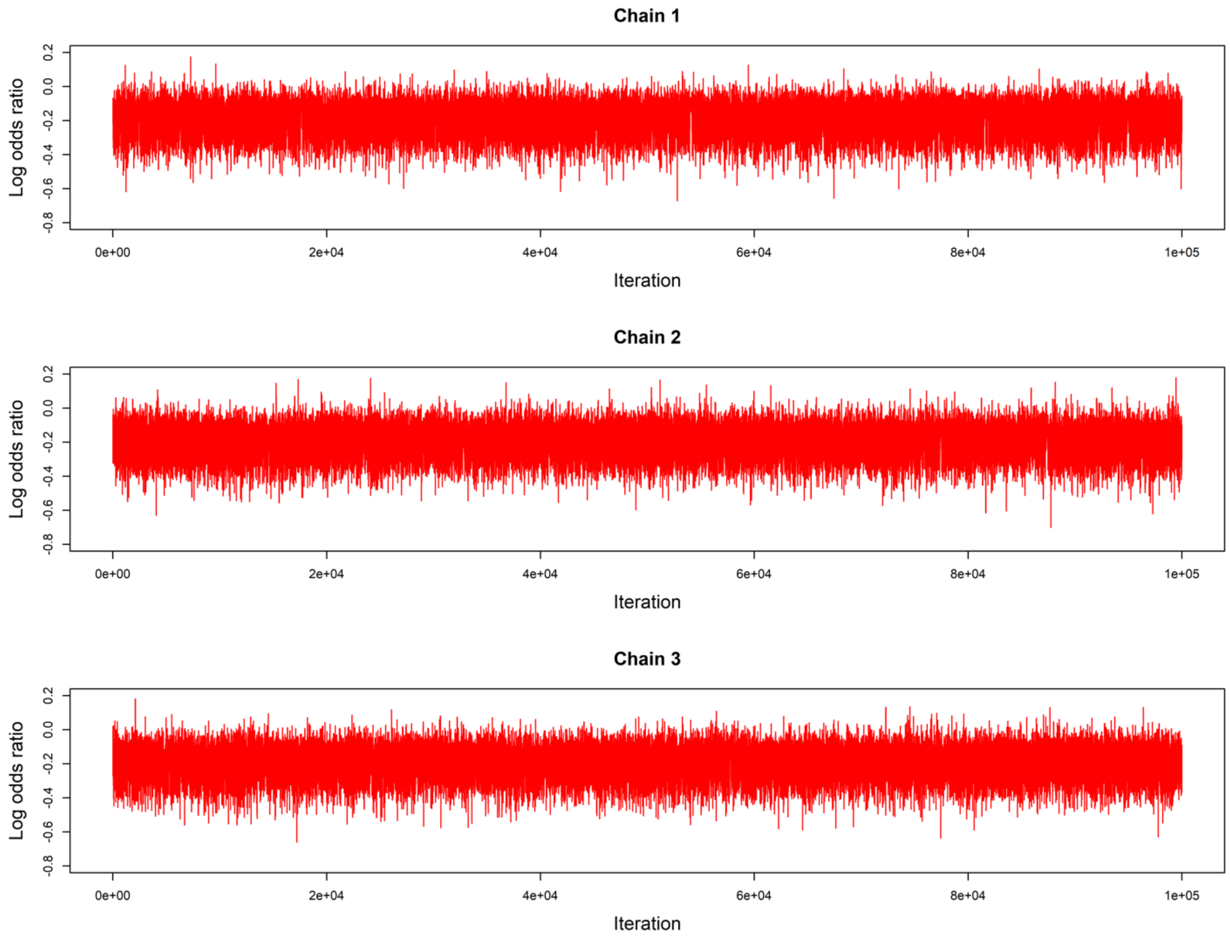

Figure S59. Trace plots of the overall log odds ratio based on the prior $HN(0, 0.1)$ for τ in the meta-analysis on acute respiratory tract infection (Example 5).

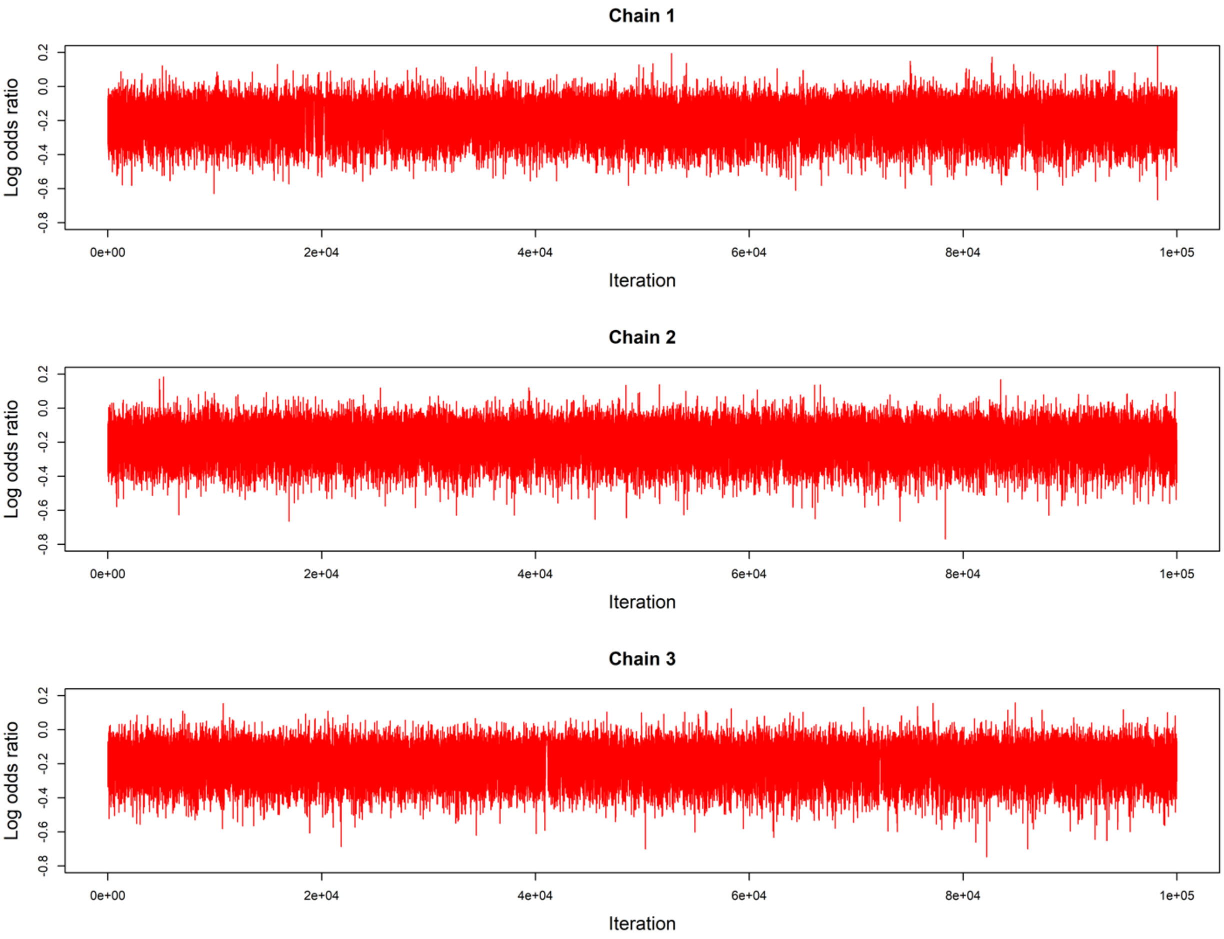

Figure S60. Trace plots of the overall log odds ratio based on the prior $HN(0, 1)$ for τ in the meta-analysis on acute respiratory tract infection (Example 5).

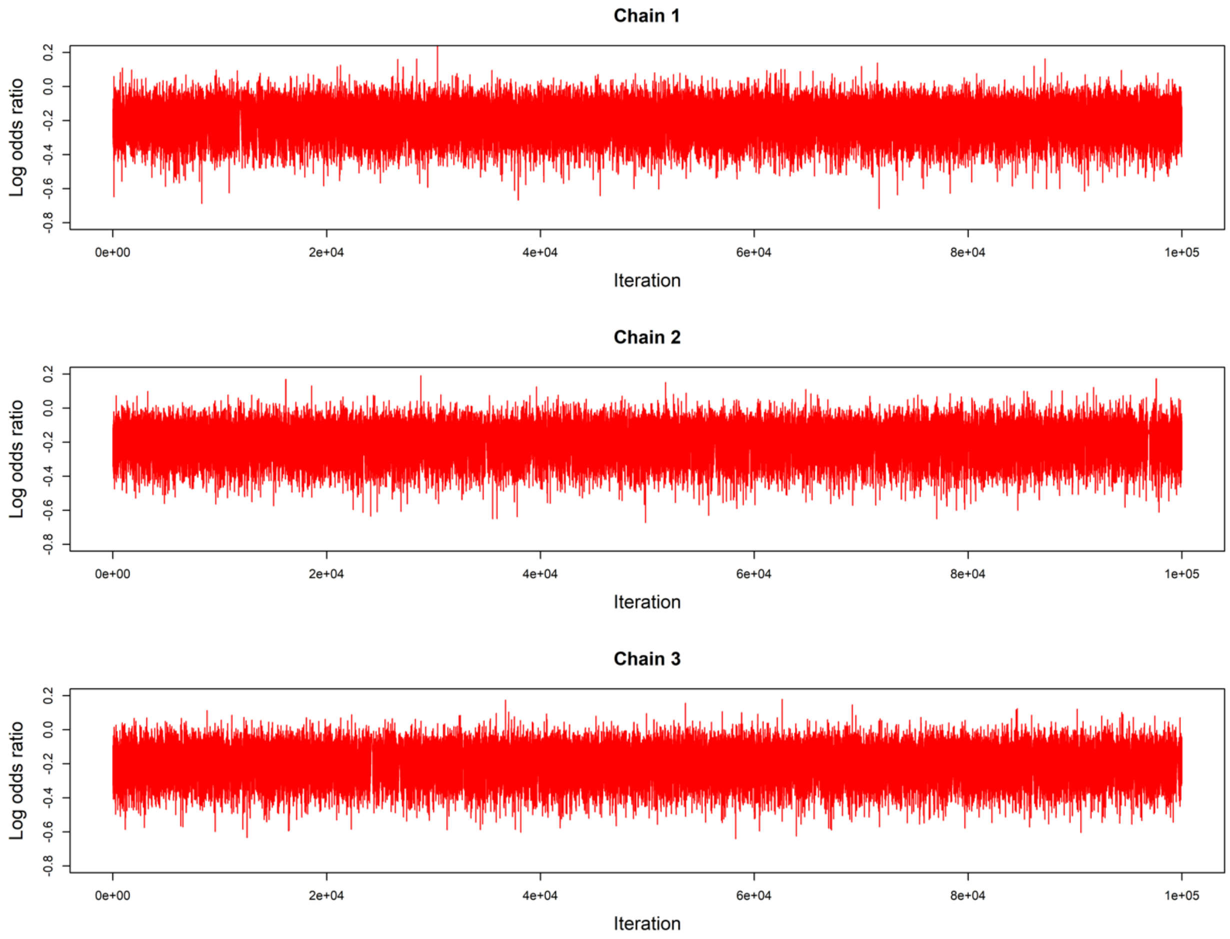

Figure S61. Trace plots of the overall log odds ratio based on the prior $HN(0, 2)$ for τ in the meta-analysis on acute respiratory tract infection (Example 5).

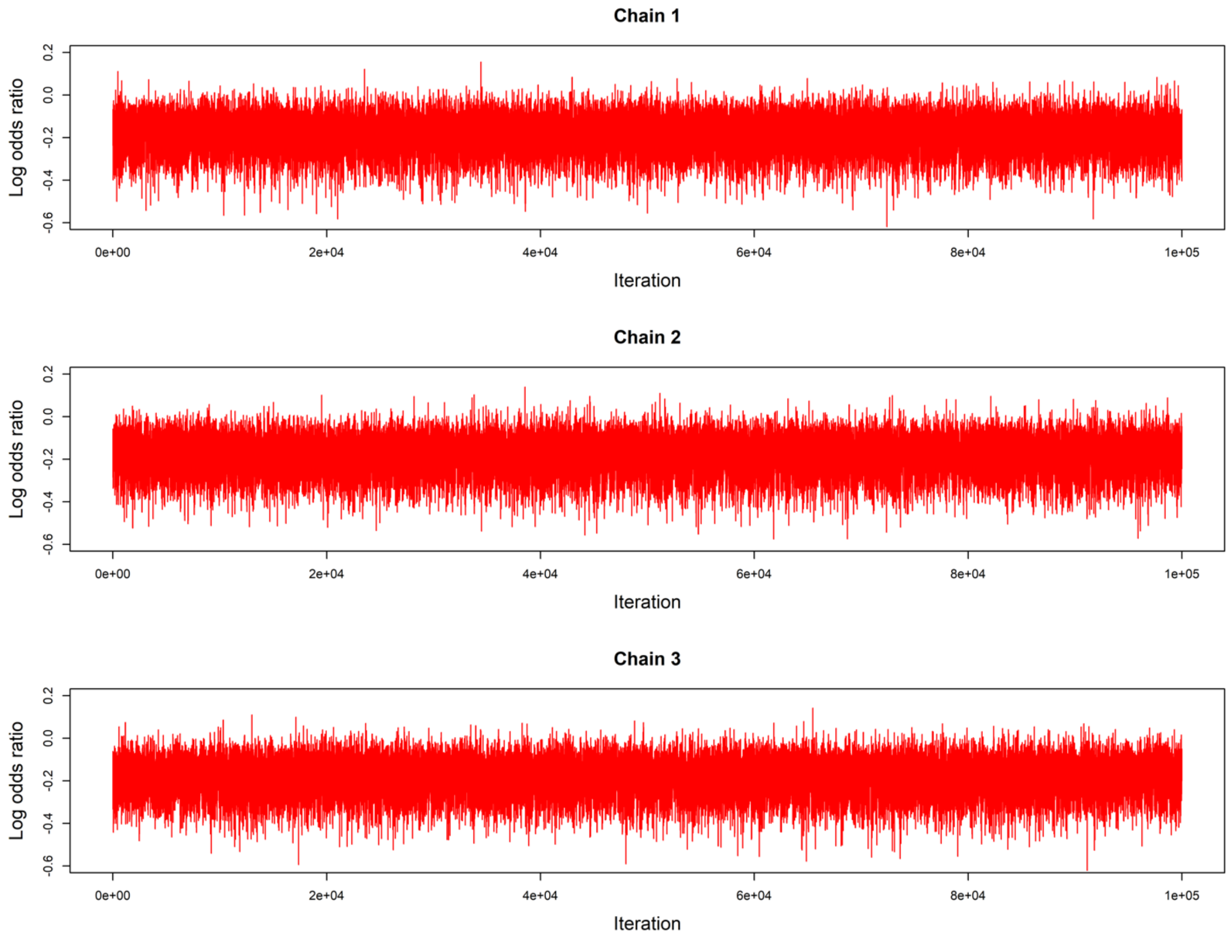

Figure S62. Trace plots of the overall log odds ratio based on the prior $LN(-4.06, 1.45^2)$ for τ^2 in the meta-analysis on acute respiratory tract infection (Example 5).

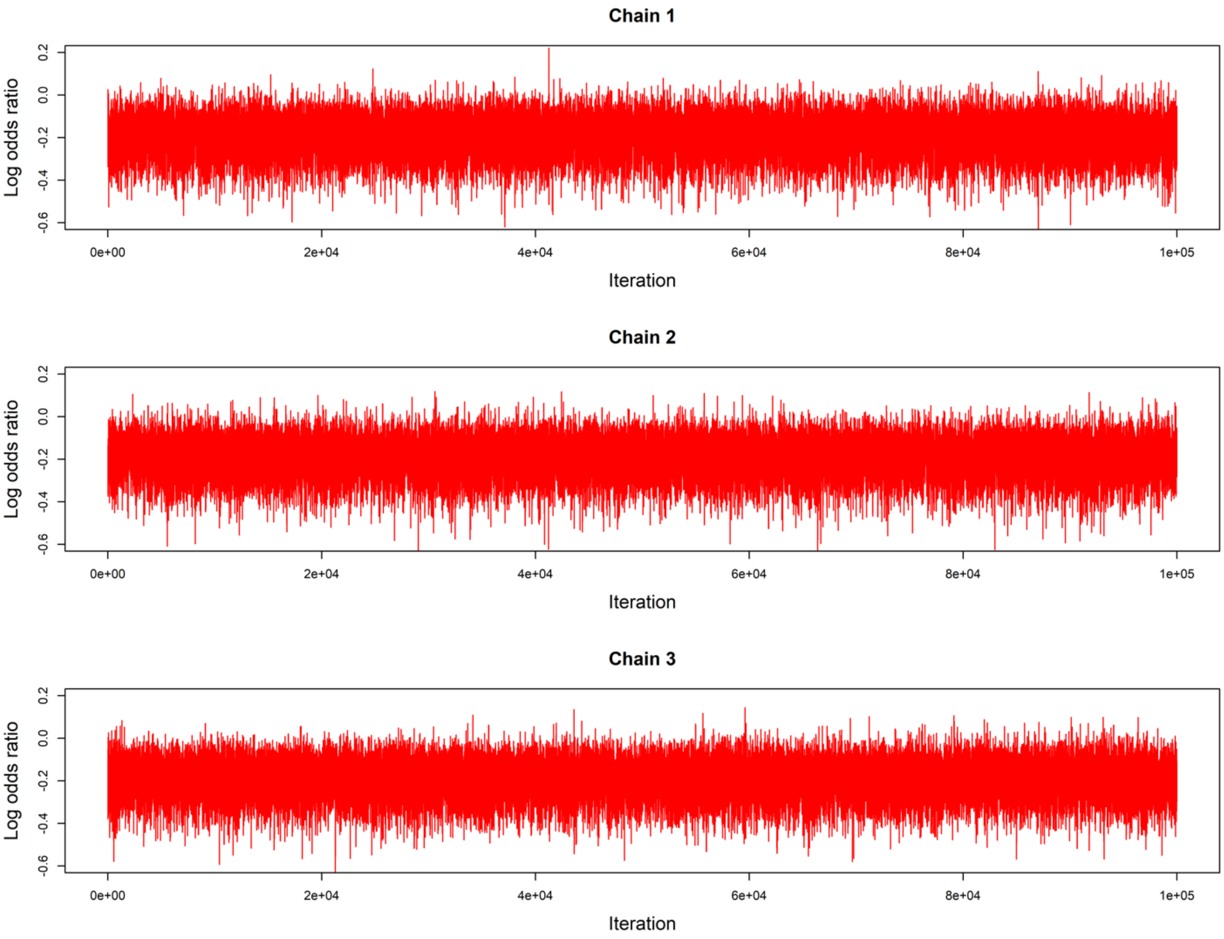

Figure S62. Trace plots of the overall log odds ratio based on the prior $LN(-3.02, 1.85^2)$ for τ^2 in the meta-analysis on acute respiratory tract infection (Example 5).

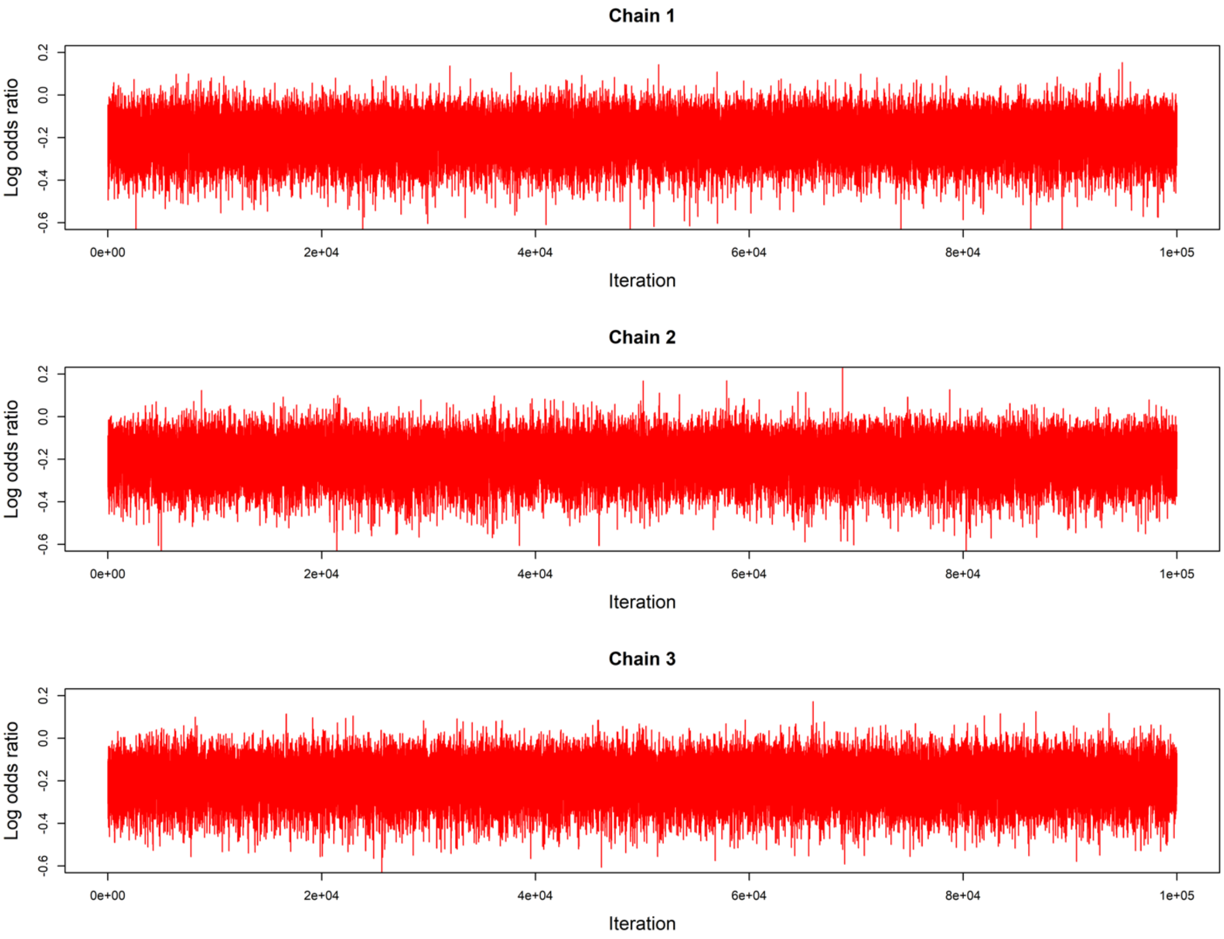

Figure S64. Trace plots of the overall log odds ratio based on the prior $LN(-2.13, 1.58^2)$ for τ^2 in the meta-analysis on acute respiratory tract infection (Example 5).

E. Posterior density plots

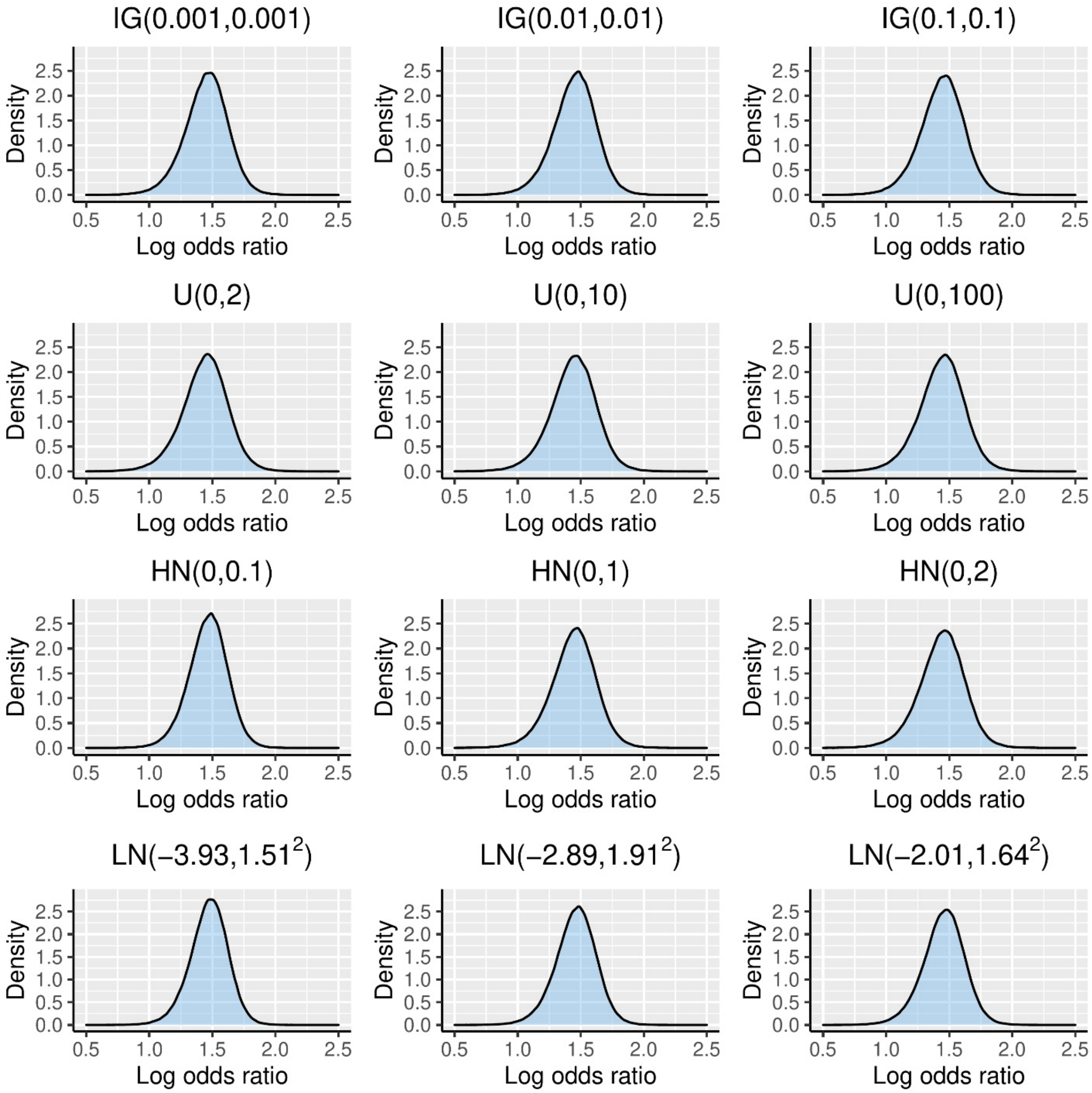

Figure S65. Posterior density plots of the overall log odds ratio based on various priors in the meta-analysis of stillbirth (Example 1).

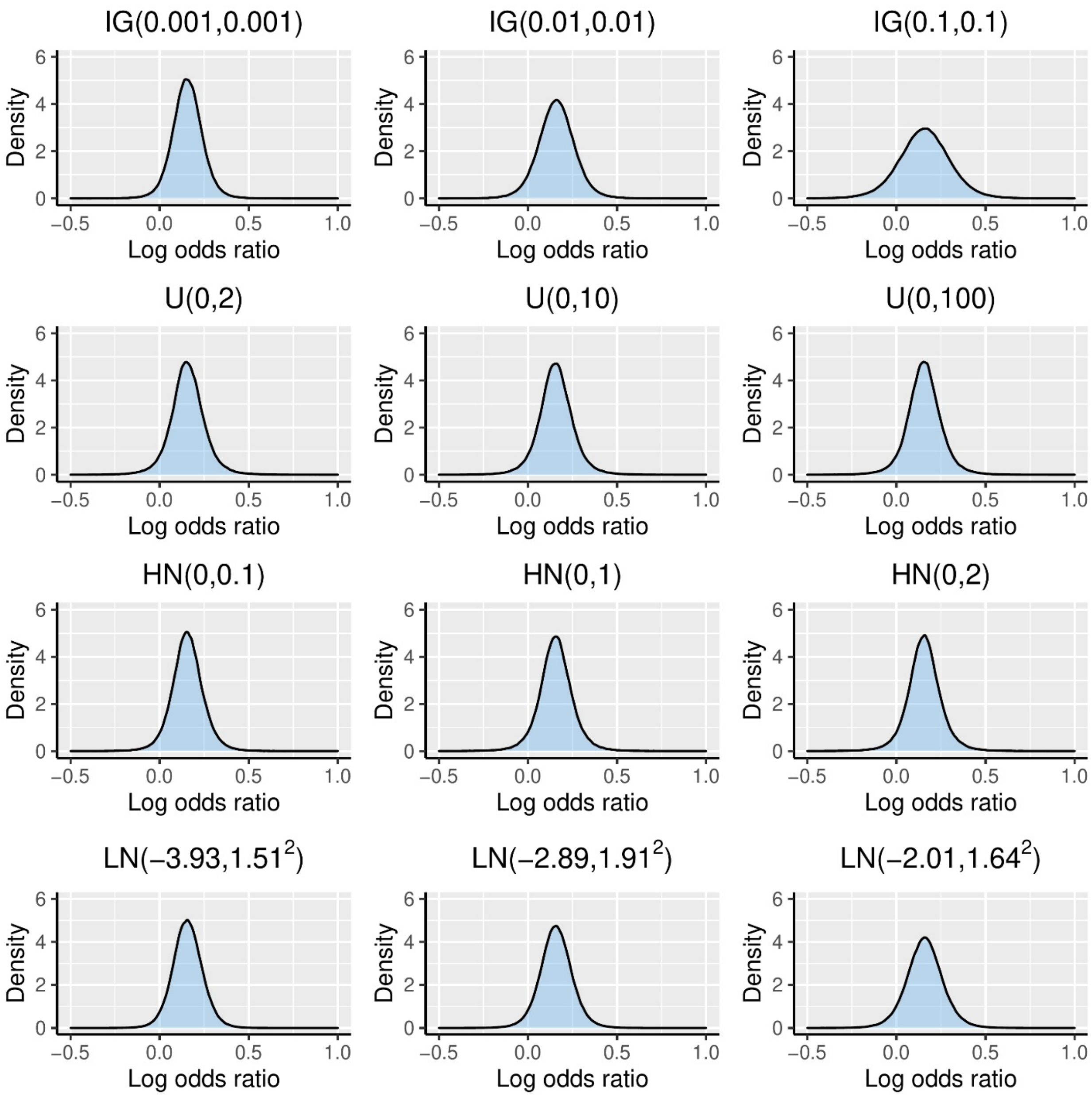

Figure S66. Posterior density plots of the overall log odds ratio based on various priors in the meta-analysis of patient enrollment in clinical trials (Example 2).

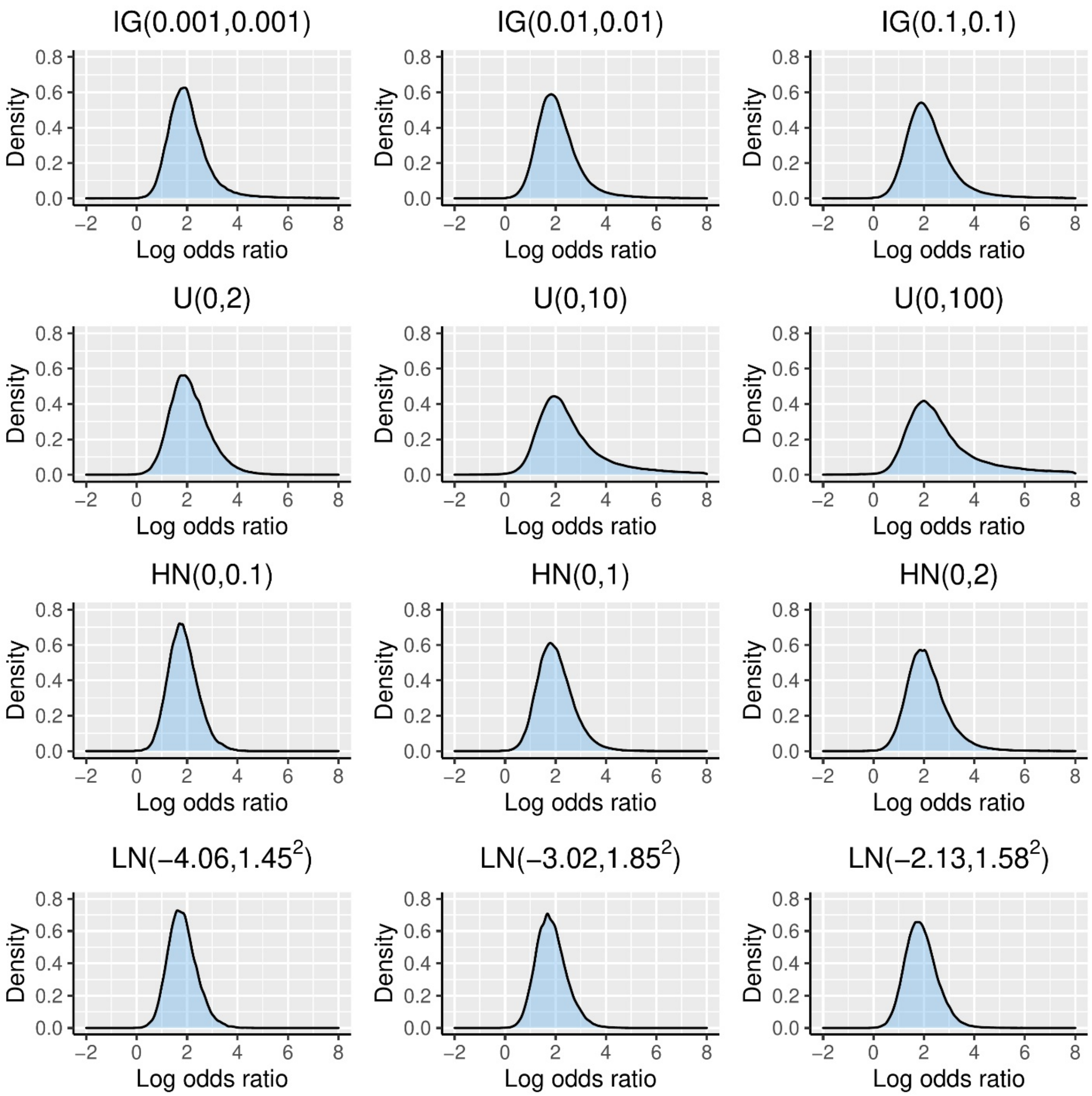

Figure S67. Posterior density plots of the overall log odds ratio based on various priors in the meta-analysis of colitis (Example 3).

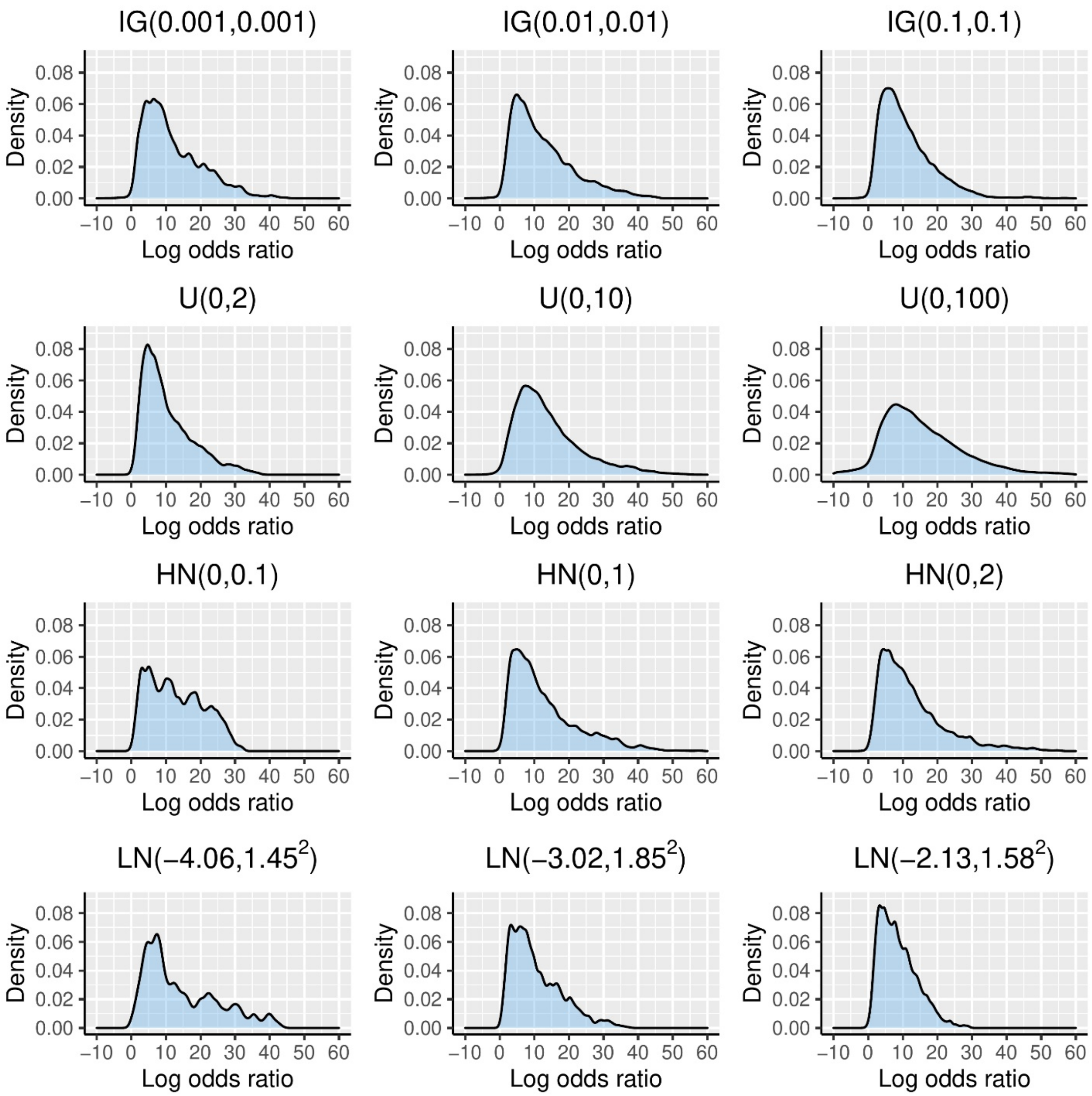

Figure S68. Posterior density plots of the overall log odds ratio based on various priors in the meta-analysis of hepatitis (Example 4).

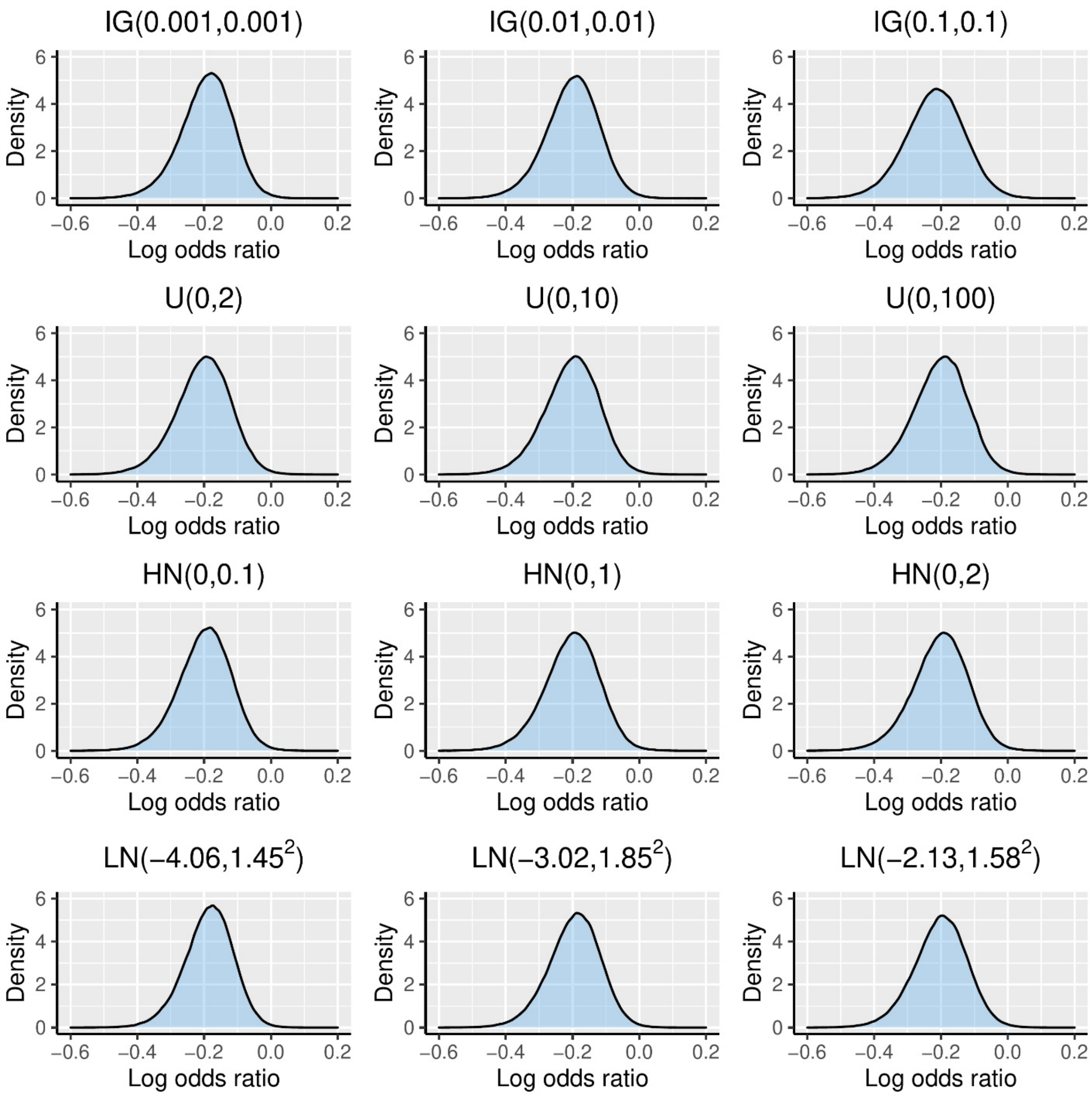

Figure S69. Posterior density plots of the overall log odds ratio based on various priors in the meta-analysis of acute respiratory tract infection (Example 5).

F. References

1. Higgins JPT. Commentary: heterogeneity in meta-analysis should be expected and appropriately quantified. *International Journal of Epidemiology.* 2008;37(5):1158-1160.
2. Veroniki AA, Jackson D, Viechtbauer W, et al. Methods to estimate the between-study variance and its uncertainty in meta-analysis. *Research Synthesis Methods.* 2016;7(1):55-79.
3. Langan D, Higgins JPT, Jackson D, et al. A comparison of heterogeneity variance estimators in simulated random-effects meta-analyses. *Research Synthesis Methods.* 2019;10(1):83-98.
4. Cornell JE, Mulrow CD, Localio R, et al. Random-effects meta-analysis of inconsistent effects: a time for change. *Annals of Internal Medicine.* 2014;160(4):267-270.
5. DerSimonian R, Laird N. Meta-analysis in clinical trials. *Controlled Clinical Trials.* 1986;7(3):177-188.
6. Normand S-LT. Meta-analysis: formulating, evaluating, combining, and reporting. *Statistics in Medicine.* 1999;18(3):321-359.
7. Harville DA. Maximum likelihood approaches to variance component estimation and to related problems. *Journal of the American Statistical Association.* 1977;72(358):320-338.
8. Viechtbauer W. Conducting meta-analyses in R with the metafor package. *Journal of Statistical Software.* 2010;36:3.
9. Chung Y, Rabe-Hesketh S, Choi I-H. Avoiding zero between-study variance estimates in random-effects meta-analysis. *Statistics in Medicine.* 2013;32(23):4071-4089.
10. Schwarzer G. meta: An R package for meta-analysis. *R News.* 2007;7(3):40-45.
11. Chang B-H, Hoaglin DC. Meta-analysis of odds ratios: current good practices. *Medical Care.* 2017;55(4):328-335.
12. Lamont K, Scott NW, Jones GT, Bhattacharya S. Risk of recurrent stillbirth: systematic review and meta-analysis. *BMJ.* 2015;350:h3080.
13. Higgins JPT, Thompson SG, Deeks JJ, Altman DG. Measuring inconsistency in meta-analyses. *BMJ.* 2003;327(7414):557-560.
14. Higgins JPT, Thomas J, Chandler J, et al. *Cochrane Handbook for Systematic Reviews of Interventions.* Chichester, UK: John Wiley & Sons; 2019.
15. Rücker G, Schwarzer G, Carpenter J, Olkin I. Why add anything to nothing? The arcsine difference as a measure of treatment effect in meta-analysis with zero cells. *Statistics in Medicine.* 2009;28(5):721-738.
16. Xie M-G, Kolassa J, Liu D, Liu R, Liu S. Does an observed zero-total-event study contain information for inference of odds ratio in meta-analysis? *Statistics and Its Interface.* 2018;11(2):327-337.
17. Ren Y, Lin L, Lian Q, Zou H, Chu H. Real-world performance of meta-analysis methods for rare events using the Cochrane Database of Systematic Reviews. *Journal of General Internal Medicine.* 2019;34(6):960-968.

18. Jackson D, Law M, Stijnen T, Viechtbauer W, White IR. A comparison of seven random-effects models for meta-analyses that estimate the summary odds ratio. *Statistics in Medicine.* 2018;37(7):1059-1085.
